# The burden of liver cirrhosis and underlying etiologies: results from the global burden of disease study 2017

**DOI:** 10.18632/aging.104127

**Published:** 2021-01-12

**Authors:** Mimi Zhai, Jianhai Long, Sushun Liu, Chun Liu, Li Li, Leping Yang, Yamin Li, Bo Shu

**Affiliations:** 1 Xiangya Nursing School, Central South University, Changsha, Hunan 410013, China; 2 Department of Respiratory, Beijing Tiantan Hospital, Capital Medicine University, Beijing 100050, China; 3 Department of General Surgery, The Second Xiangya Hospital, Central South University, Changsha, Hunan 410011, China; 4 Clinical Nursing Teaching and Research Section, The Second Xiangya Hospital, Central South University, Changsha, Hunan 410011, China

**Keywords:** liver cirrhosis, etiology, global burden of disease study

## Abstract

Background: To evaluate the pattern and prevalence trends of liver cirrhosis caused by specific etiologies.

Results: Globally, the number of prevalent cases increased 74.53% from 1990 to 2017. The ASR increased 0.75 per year. The most pronounced increases were found in middle-high and high socio-demographic index (SDI) regions, especially in the Caribbean and Latin America. Among the etiologies, non-alcoholic steatohepatitis (NASH) related liver cirrhosis accounted for 59.46% of the cases. The ASR increased 1.74 per year, and the increase was observed in all 5 SDI regions. In addition, the ASR of liver cirrhosis caused by alcohol also increased in both sexes and all SDI regions. In contrast, the ASR of liver cirrhosis caused by hepatitis B virus (HBV) and hepatitis C virus (HCV) decreased, especially in middle and low-middle SDI regions.

Conclusions: Though the number of people suffering from HBV and HCV decreases, liver cirrhosis is still a major threat to health. Additionally, the number of people with cirrhosis caused by alcohol and NASH continues to grow. Thus, more targeted and specific strategies should be established based on etiology and prevalence trends of liver cirrhosis.

Methods: We collected data based on Global Burden of Disease (GBD) 2017 study. The age standardized prevalence rate (ASR) and estimated annual percentage changes (EAPC) were used to estimate the trends in prevalence by population, etiologies and regions.

## INTRODUCTION

Liver cirrhosis is a major health issue that afflicted more than 160 million people in 2017 worldwide [1]. Previous studies have documented that the incidence of liver cirrhosis varies, and chronic viral hepatitis is the most common cause, especially hepatitis B virus (HBV) and hepatitis C virus (HCV) [2, 3]. The highest incidence of liver cirrhosis is found in East Asia. In contrast, the incidence in Southern Latin America is the lowest, with a value of 12.1% [2]. Moreover, newly diagnosed cases continue to increase globally in the last few decades, although many public health initiatives have been implemented.

The major causative agents of liver cirrhosis include HBV, HCV, alcohol use, non-alcoholic steatohepatitis (NASH) and other causes [4]. The etiologies of liver cirrhosis in each region or country varies bases on the different risk factors. For instance, HBV is the main cause of liver cirrhosis in China [5]. In Mexico, Japan and the United States, liver cirrhosis is mainly caused by HCV. Moreover, the number of patients with cirrhosis caused by alcohol and NASH is continuously increasing. Thus, more targeted prevention strategies should be implemented based on the trends in liver cirrhosis.

The Global Burden of Disease (GBD) study provides estimates of the liver cirrhosis burden in 195 countries and territories. By using the latest GBD 2017 study data, researchers can extensively investigate the trends and landscape of liver cirrhosis throughout the world. Bosetti et al. studied the worldwide mortality of cirrhosis in 2002, and de Carvalho et al. described the burden of liver cirrhosis in Brazil in 2017 [2, 4]. Additionally, a recent study mainly demonstrated the trend of liver cirrhosis mortality worldwide. It indicated that hepatitis was the main causes of cirrhosis, but the impact caused by hepatitis might be attenuated and overtaken by NASH in future [6]. However, rare studies have investigated the landscape of liver cirrhosis worldwide via the newest GBD data. In addition, none study has analyzed the relationship between sociological indicators and cirrhosis. In this study, we used GBD data to evaluate detailed information on liver cirrhosis. We also investigated the burden of liver cirrhosis by using the prevalence of various etiologies and overall liver cirrhosis prevalence.

## RESULTS

### Global liver cirrhosis burden

**Figure 1:**
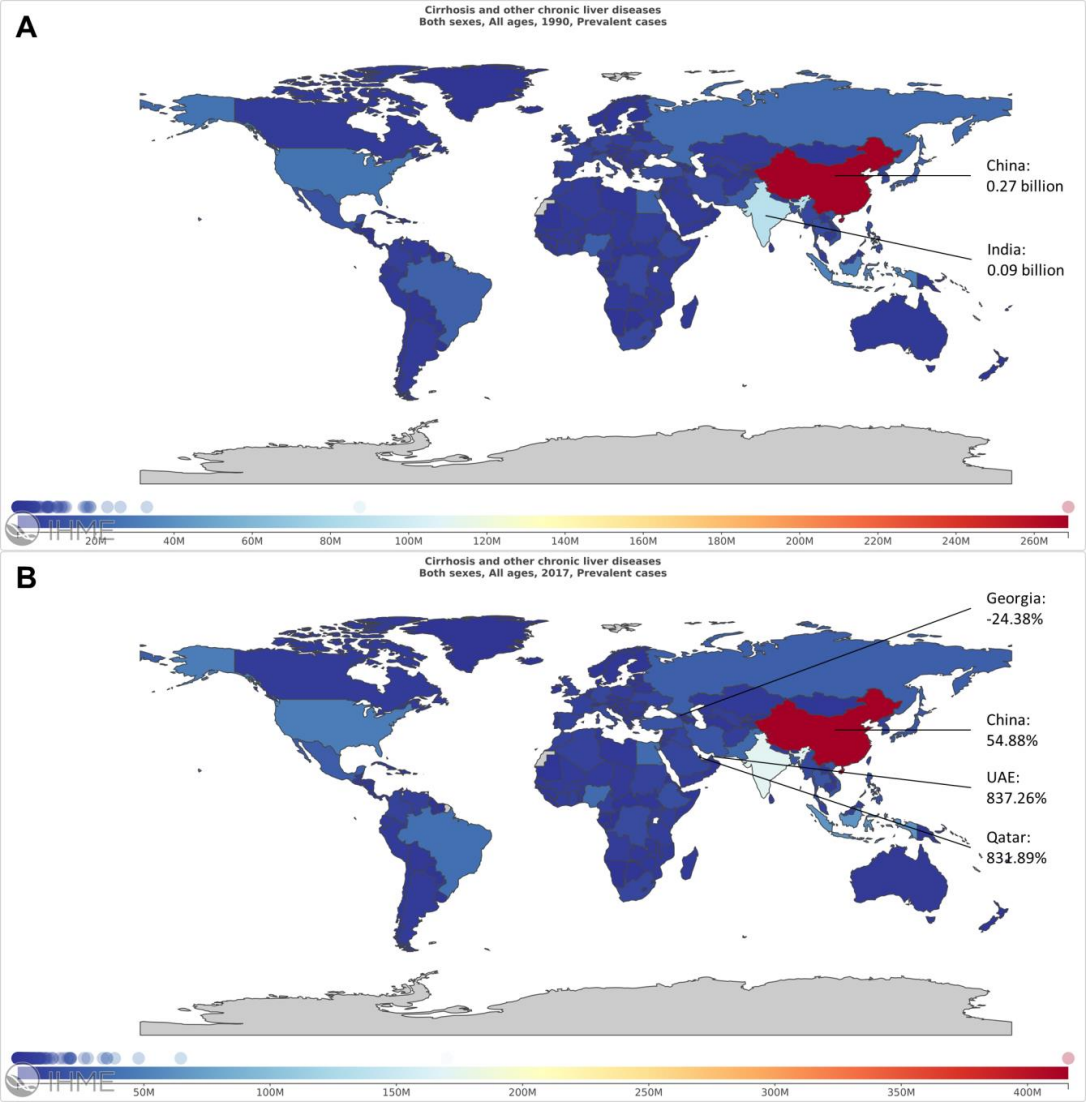
**The worldwide prevalence cases of liver cirrhosis in 195 countries and territories.** (**A**) The worldwide prevalence cases of liver cirrhosis in 1990. (**B**) The worldwide prevalence cases of liver cirrhosis in 2017.

**Figure 2:**
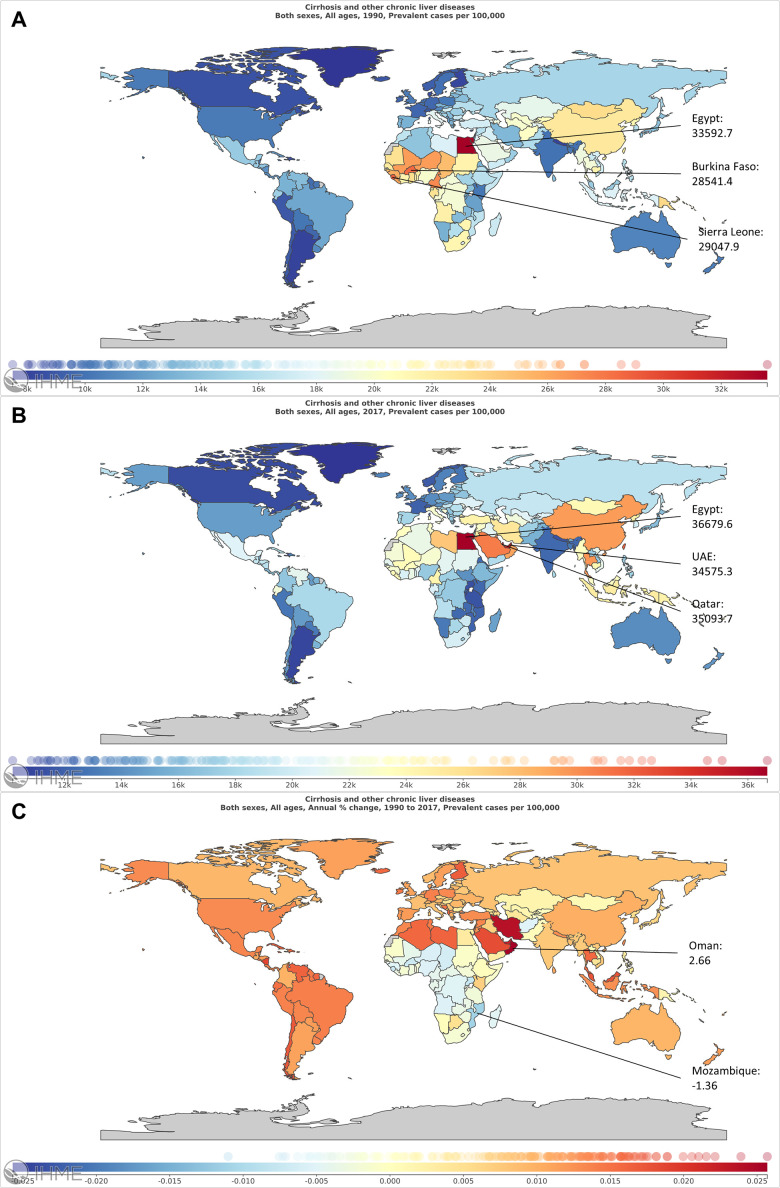
**The global burden of liver cirrhosis in 195 countries and territories.** (**A**) The ASR of liver cirrhosis in 1990. (**B**) The ASR of liver cirrhosis in 2017. (**C**) The EAPC of liver cirrhosis from 1990 to 2017.

Globally, the prevalent cases increased 74.53%. Although, China had the largest number of patients in 1990 (0.27 billion) and in 2017 (0.42 billion) (Figure 1A, 1B and Supplementary Table 6), the growth rate was only 54.88%. The United Arab Emirates (UAE) had the highest growth rate with a value of 837.26% (Figure 1B and Supplementary Table 6). The worldwide ASR was 19640.0 per 100000 in 2017 (Figure 2A, 2B and Supplementary Table 7). Among all the countries, the highest ASR was found in Egypt, followed by Qatar and United Arab Emirates (Figure 2A, 2B). The worldwide ASR increase 0.75 per year (95% CI 0.73-0.77) (Figure 2C and Supplementary Table 7), and the fastest increase was found in Oman, followed by Iran, Saint Vincent and the Grenadines (Figure 2C). Contrarily, 40 of the 195 countries and territories demonstrated a decrease trend in ASR, and the fastest reduction was observed in Mozambique (Figure 2C).

**Figure 3:**
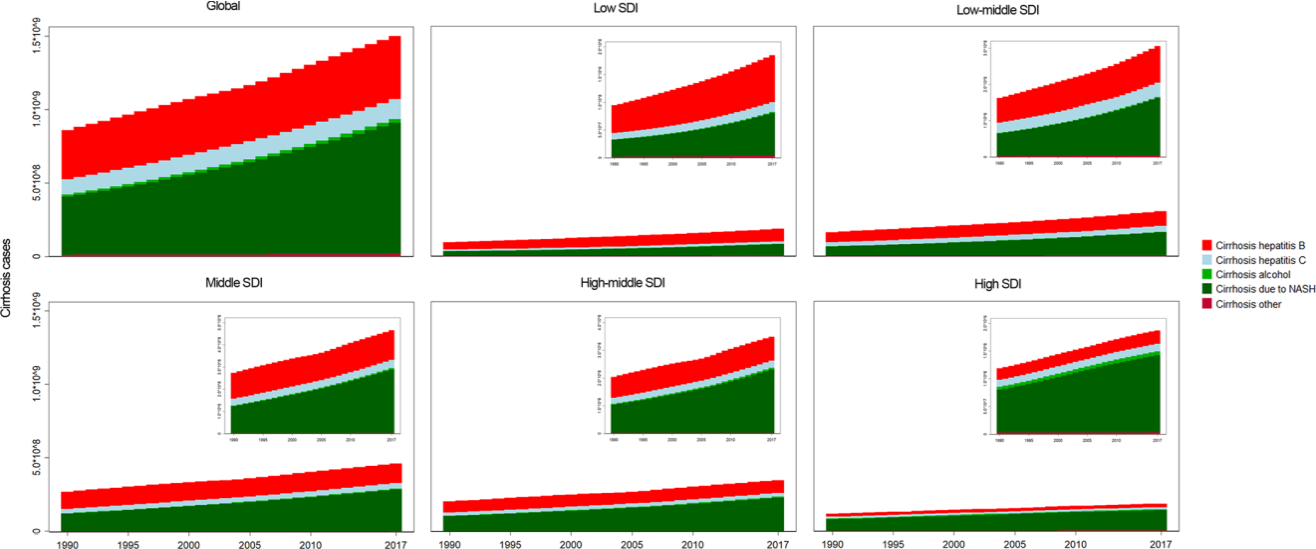
**The analysis of liver cirrhosis and its etiologies.** The liver cirrhosis cases caused by different etiologies, by SDI regions.

**Figure 4:**
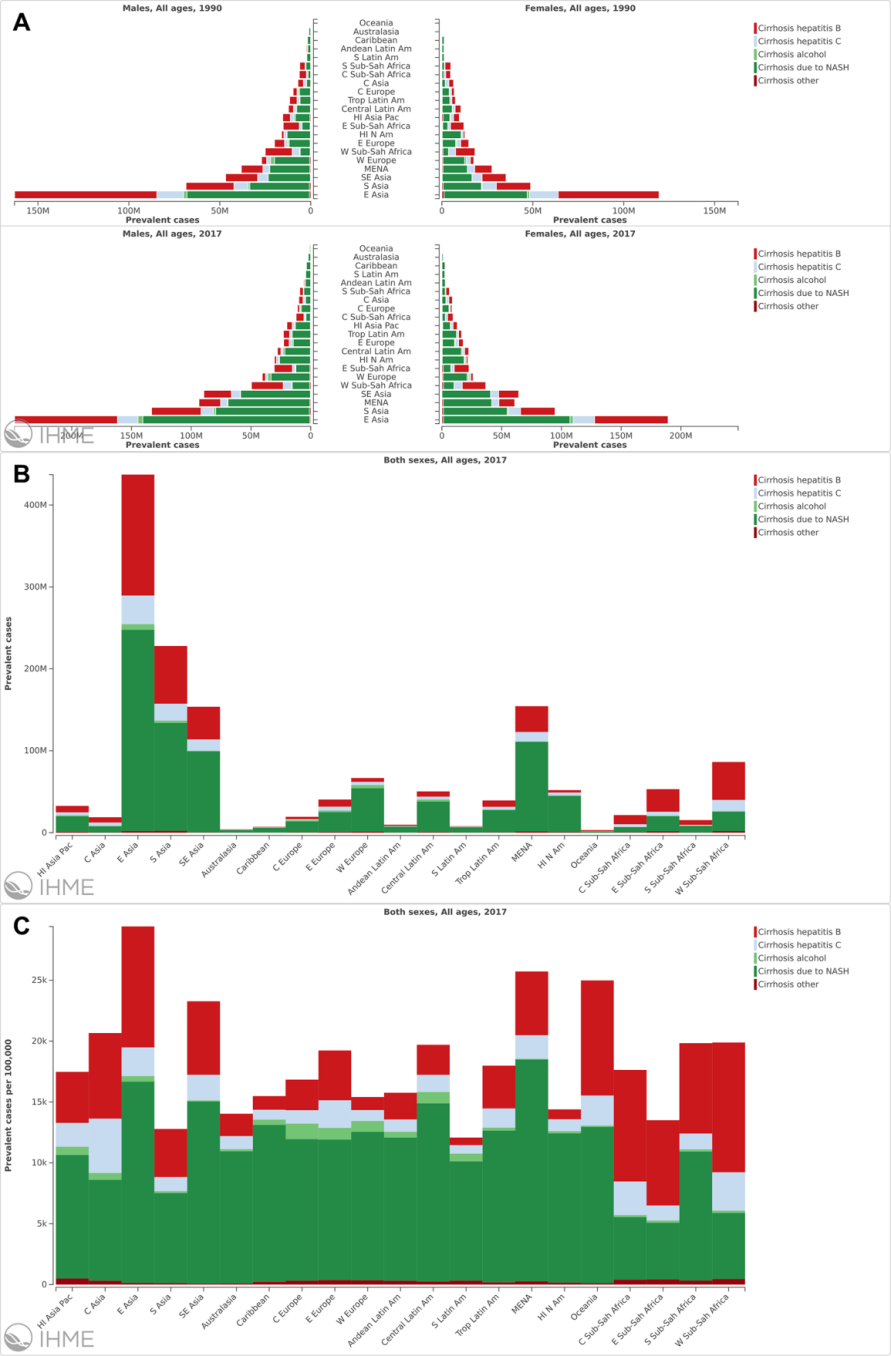
**The analysis of liver cirrhosis at a regional level.** (**A**) The prevalence cases of liver cirrhosis caused by different etiologies in different region and sex. (**B**) The prevalence cases of liver cirrhosis in different regions. (**C**) The ASR of liver cirrhosis in different regions.

The prevalence cases increased in all 5 SDI regions, especially in the middle-high SDI regions (Figure 3 and Supplementary Table 7). For geographical regions, the prevalent cases increased in all 21 regions (Figure 4A). Country with the largest number of liver cirrhosis cases was East Asia, followed by South Asia and Southeast Asia (Figure 4B). The highest ASR in 2017 was also found in East Asia (Figure 4C and Supplementary Table 7). Additionally, the fastest increase in ASR was found in the Caribbean (Supplementary Table 7). In contrast, the fastest decrease in ASR was found in Western Sub-Saharan Africa, with an EAPC of -0.63 (95% CI -0.72--0.55) (Supplementary Table 7).

**Figure 5:**
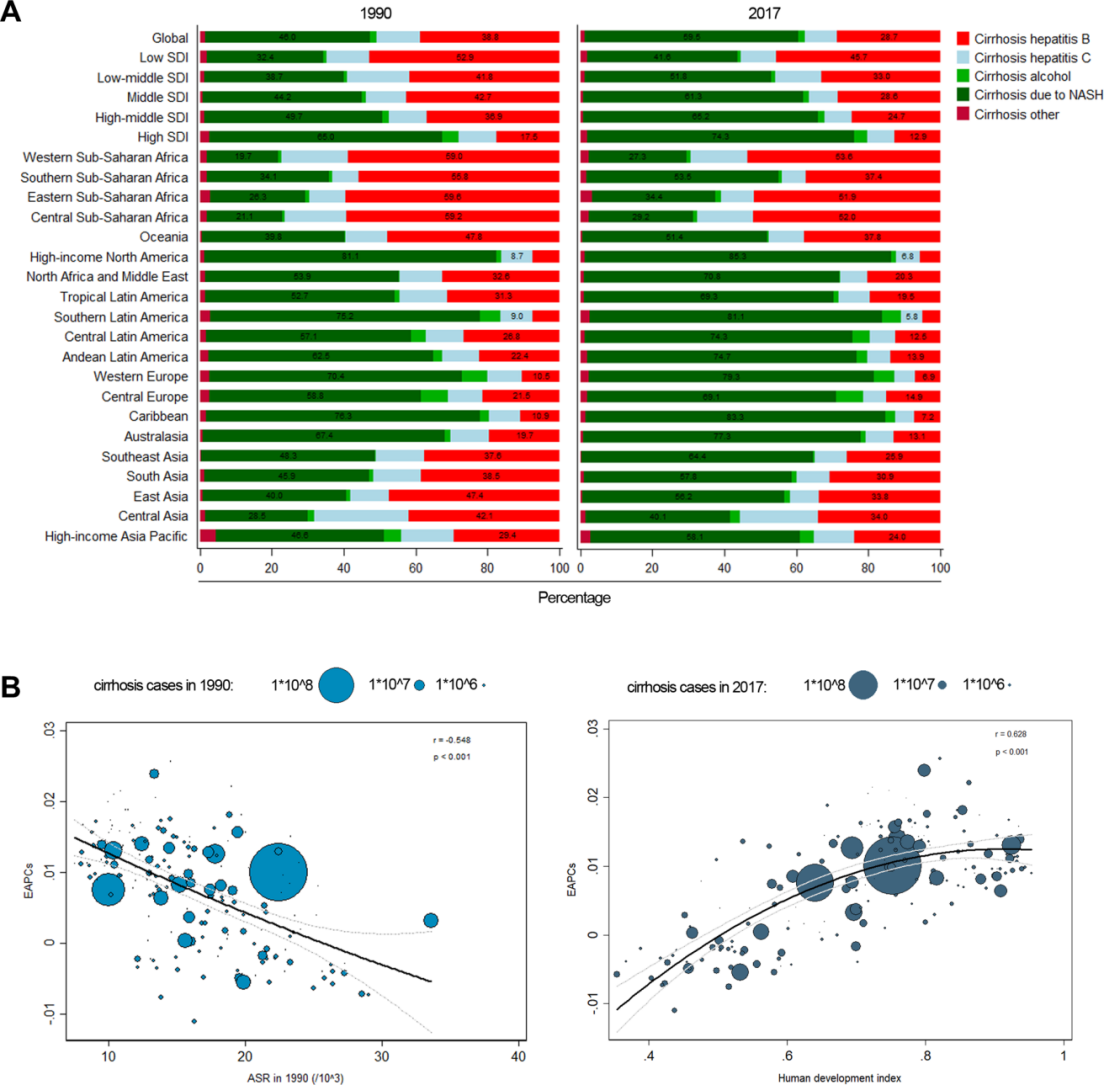
(**A**) Composition ratio of each etiology in prevalence cases of liver cirrhosis in 1990 and 2017. (**B**) The correlation between EAPC and ASR in 1990, HDI in 2017.

For etiologies of liver cirrhosis, NASH was the main etiology with 0.89 billion prevalent patients (59.46% of liver cirrhosis cases). A total of 0.43 billion prevalent cases were infected with HBV (28.72%) (Figure 5A and Supplementary Table 7). Additionally, the fastest increase in ASR was also found in patients with NASH (EAPC=1.74 95% CI 1.73-1.75). Contrarily, The ASR decreased in patients infected with HBV and HCV from 1990 to 2017 (Supplementary Table 7).

### The influential factors for EAPC

A significant relationship was studied between EAPC and ASR, HDI, respectively. The ASR of liver cirrhosis could be considered the disease reservoir at baseline. Additionally, the HDI could be considered the level of available medical resources. In or study, the EAPC was negatively correlated with the ASR in 1990 (r=-0.548, *p<*0.001) (Figure 5B). In contrast, the EAPC was positively correlated with the HDI in 2017 (r=0.628, *p<*0.001) (Figure 5B). The results demonstrated that countries with higher HDI have suffered a more rapid increase in ASR of liver cirrhosis.

### Liver cirrhosis due to NASH

Globally, approximately 59.46% of patients with liver cirrhosis had NASH in 2017 (Figure 5A and Supplementary Table 7). The prevalent cases increased 125.61% from 1990 to 2017 (Supplementary Table 1). The country with the largest number of NASH-induced liver cirrhosis patients was China, and its growth rate was 117.89% (Supplementary Figure 1A, 1B and Supplementary Table 6). The highest ASR was observed in Qatar in 1990 and in UAE in 2017 (Supplementary Figure 2A, 2B). The ASR increased 1.74 per year (95% CI 1.73-1.75) (Supplementary Table 1). The fastest increase in ASR was found in Oman (Supplementary Figure 2C). Among all the countries, 193 of them demonstrated an increase trend in ASR; Afghanistan and Nigeria did not. The ASR also increased in all 5 SDI regions, especially in the middle and middle-high SDI regions (Supplementary Table 1). For the geographical regions, the prevalent cases and the ASR both increased in all 21 regions (Supplementary Figure 2C and Supplementary Table 1). In addition, the fastest increase in ASR was observed in Western Sub-Saharan Africa (Supplementary Table 1).

### Liver cirrhosis due to hepatitis B

The prevalent cases of liver cirrhosis caused by hepatitis B increased 29.16% during the period (Supplementary Table 2). Globally, approximately 28.72% of patients with prevalent liver cirrhosis had HBV in 2017 (Figure 5A). China had the largest number of patients in the world (Supplementary Figure 3A, 3B). Qatar had the largest increase with a growth rate of 568.30% (Supplementary Figure 3B and Supplementary Table 6). The highest ASR was found in Sierra Leone (Supplementary Figure 4A, 4B). Additionally, the ASR of liver cirrhosis caused by HBV displayed a minor decrease with an EAPC of -0.39 (95% CI -0.46--0.34) (Supplementary Table 2). The highest EAPC was found in Taiwan. In contrast, the lowest EAPC was observed in Mexico. The ASR decreased in all 5 SDI regions, especially in the Middle SDI regions (Supplementary Table 2). For the geographical regions, the ASR also decreased in all 21 regions (Supplementary Figure 4C and Supplementary Table 2).

### Liver cirrhosis due to hepatitis C

The prevalence of liver cirrhosis caused by HCV increased 28.74% (Supplementary Table 3). China also had the largest number of HCV patients in the world (Supplementary Figure 5A, 5B). The UAE had the fastest increase rate in the number of HCV patients (Supplementary Figure 5B and Supplementary Table 6). The highest ASR was observed in Egypt (Supplementary Figure 6A, 6B). Similar to the liver cirrhosis caused by HBV, the ASR of HCV-related liver cirrhosis displayed a decrease trend with an EAPC of -0.39 (95% CI -0.42--0.36) (Supplementary Table 3). The fastest ASR increase was found in Iran, and the fastest ASR decrease was found in Equatorial Guinea (Supplementary Figure 6C). Additionally, the ASR also decreased in all 5 SDI regions. Amazing, the ASR increased in Eastern Europe and North America–high income regions (Supplementary Figure 6C and Supplementary Table 3).

### Liver cirrhosis due to alcohol use

The prevalence of liver cirrhosis caused by alcohol increased 78.27% (Supplementary Table 4). China also had the largest number of patients related to alcohol use in the world (Supplementary Figure 7A, 7B). The UAE had the highest increase of the number of cirrhosis patients caused by alcohol with a growth rate of 863.93% (Supplementary Figure 7B and Supplementary Table 6). Hungary and Slovakia had the highest ASR in 1990 and in 2017, respectively (Supplementary Figure 8A, 8B). Globally, the ASR increased 0.84 (95% CI 0.79-0.89) per year. With respect to the individual countries, the fastest increase of ASR was observed in Vietnam (Supplementary Figure 8C). Additionally, the ASR increased in all 5 SDI regions (Supplementary Table 4). The ASR also increased in all of the geographical regions except for Asia Pacific–high income, Western Sub-Saharan Africa and Southern Sub-Saharan Africa (Supplementary Figure 8C, Supplementary Table 4).

### Liver cirrhosis due to other causes

The prevalence of liver cirrhosis caused by other causes increased 45.30% from 1990 to 2017 (Supplementary Table 5). Although China also had the largest number of patients related to the other causes, the growth rate was 4.10%. The highest growth rate was found in Qatar, with a value of 409.68%, and the lowest growth rate was found in Bosnia and Herzegovina (Supplementary Figure 9A, 9B and Supplementary Table 6). The highest ASR was observed in South Korea and Brunei (Supplementary Figure 10A, 10B). Additionally, the global ASR remained stable during this period with an EAPC of 0.04 (95% CI -0.02-0.10) (Supplementary Figure 10C and Supplementary Table 5). The ASR increased in the low and low-middle regions. For geographical regions, the ASR decreased in 7 regions, including Asia Pacific–high income, East Asia, Central Europe, etc. (Supplementary Figure 10C and Supplementary Table 5).

## DISCUSSION

The worldwide prevalence of liver cirrhosis continues increase [7]. The heterogeneous pattern in risk factors is different [8, 9]. Although the incidence of HBV and HCV continuously decreases, the ever-increasing incidence of liver cirrhosis caused by alcohol and NASH remains a formidable threat [10, 11].

In our study, we analyzed the trends of liver cirrhosis. In general, the prevalence of liver cirrhosis continuously increased. The trends were mainly dominated by an increase of NASH-induced liver cirrhosis, with a smaller contribution from alcohol use. The prevalent cases of HBV and HCV increased during the period, but the ASR decreased [12]. The decrease trends of HBV and HCV ASR were mainly caused by the decrease in the number of patients with HBV- and HCV-related liver cirrhosis [13, 14]. In contrast to the previous result that hepatitis was the main cause of liver cirrhosis in the prevalent cases, liver cirrhosis caused by NASH occupied a major position [12]. Consequently, exploring the exact pattern of liver cirrhosis etiologies was important for developing specific preventive measures. Additionally, the HDI and EAPC were found to be positively correlated in our study. HDI was a summary measure indicative of a long and healthy life, being knowledgeable and having a decent standard of living. The HDI simplified and captured only part of human development details. Moreover, patients with liver cirrhosis had a long survival time. With the increase in the life expectancy index, the prevalence of patients with liver cirrhosis also increased. Thus, the dimensions of HDI might be positively correlated with EAPC. Moreover, a study conducted by Liu et al. also indicated that the HDI and EAPC were positively correlated in patients with liver cancer [1]. In 1990 and 2017, 46% and 59% of liver cirrhosis patients, respectively, had NASH. The fastest ASR increase in prevalent cases was also found in patients with NASH, which was different from the results found in other studies [1, 15]. Additionally, in contrast to other studies that used incidence as an indicator, we used prevalence as the indicator. Because patients with liver cirrhosis had a long survival time, it was more reasonable to use prevalence as the indicator. In addition, we further analyzed the reason why NASH accounts for the highest proportion in prevalent cases. By analyzing the GBD data, we found that liver cirrhosis caused by hepatitis accounted for the highest proportion of deaths, while liver cirrhosis caused by NASH accounted for the lowest proportion of deaths. Therefore, more and more patients had cirrhosis caused by NASH over time, but fewer patients died, resulting in a higher proportion of prevalent cases. A study revealed that the annual percentage change of mortality of NASH-induced cirrhosis was 3-fold greater than that for alcohol-induced cirrhosis, and NASH surpassed alcohol and hepatitis to be the leading cause of liver cirrhosis in the United States [16]. This might be related to the obesity epidemic in the United States, and the burden of NASH-induced liver cirrhosis might increase over the next decade [17]. Thus, public policy, which focused on primary prevention, prompt diagnosis, and pre-emptive therapy should establish plans to raise awareness and decrease the disease burden of NASH.

HBV was an important risk factor for liver cirrhosis in some regions [6, 18]. Moreover, HBV infection contributed to half of the mortality associated with liver cancer [19]. In our study, we found that liver cirrhosis caused by HBV was more prevalent in the low-middle and middle SDI regions. Additionally, more than 50% of patients with liver cirrhosis in Africa were caused by HBV, and nearly 40% of the cases in East Asia, Central Asia and Oceania were also caused by HBV. By promoting HBV vaccination, the ASR of the 21 regions decreased over the last few decades. Although China had the largest number of HBV patients, the growth rate was only 10.21% from 1990 to 2017. This was mainly due to the promotion of HBV vaccines in China [20]. By implementing these measures, the number of patients suffering from HBV infection was significantly suppressed in the general population [21, 22]. Amazingly, although the ASR decreased during the period in all 5 SDI regions, the slowest decrease was found in high the SDI regions, such as North America–high income and South Asia. This finding indicates that more effective public measures to prevent HBV should be implemented in these countries [23]. Moreover, the development of anti-HBV drugs, such as entecavir and tenofovir, has further reduced the number of patients [24]. Thus, we can expect that the number of patients with liver cirrhosis caused by HBV will be significantly decreased in the future.

Similar to liver cirrhosis caused by HBV, the ASR of liver cirrhosis caused by HCV also decreased. Additionally, the ASR of liver cirrhosis caused by HCV also decreased in all 5 SDI regions. To our surprise, the ASR increased in Eastern Europe, Tropical Latin America and North America–high income, which was not the same as the results reported in the study conducted by The Polaris Observatory HCV Collaborators [25]. China also had the largest number of patients with HCV, but the growth rate was 34.01% [26]. This might be related to a lack of effective treatment measures before 2014. Subsequently, direct-acting antiviral therapy was introduced, and more than 90% of patients with all genotypes of HCV could be cured [27]. As a result, interventions should be introduced all over the world, such as promoting direct-acting antiviral therapy and reducing the therapy price.

Alcohol was proven to be a major risk factor for liver cirrhosis. The ASR of liver cirrhosis caused by alcohol use increased from 1990 to 2017. The results obtained by Asrani et al. also revealed that alcohol use and NASH had overtaken hepatitis as the primary causes of liver diseases in Western countries [28]. The increase in ASR was higher in females than in males, similar to the result obtained by Roerecke et al. [29]. Additionally, alcohol was found to play an increasingly important role in chronic liver diseases [30]. Thus, polices to reduce alcohol consumption should be implemented, and people with high alcohol consumption should receive interventions to reduce their intake [29].

Although the GBD data demonstrated the temporal trend in the prevalence of liver cirrhosis, several limitations should be noted. The accuracy of results obtained from GBD data depend on the quality and quantity of liver cirrhosis data. In some countries or regions, the liver cirrhosis data were incomplete or even missing. This might lead to an underestimation of the severity of liver cirrhosis. Additionally, we could only study the temporal trend in the prevalence of liver cirrhosis by each etiology, and the interaction between etiologies could not be studied via the GBD data. Moreover, the GBD data of liver cirrhosis was not classified by decompensation and compensation. Thus, we could not discuss the difference between compensated and decompensated cirrhosis in terms of epidemiology.

In summary, liver cirrhosis poses a huge threat to people's health. Although the ASR of liver cirrhosis caused by hepatitis decreased with HBV vaccination and direct-acting antiviral therapy, the ASR of liver cirrhosis caused by alcohol and NASH continued to grow during the study period. Thus, public health priorities that target alcohol consumption and NASH should be implemented as soon as possible.

## MATERIALS AND METHODS

### Study data

Detailed information on liver cirrhosis was obtained from the GBD 2017 study. By using the sociodemographic index (SDI), the 195 countries and territories in the GBD 2017 study were divided into 5 groups. The 195 countries and territories were grouped into 21 regions according to their geographical location. The method of extracting data and the estimation method of liver cirrhosis disease burden were based on the Liu et al. study [1]. Moreover, we collected and analyzed the human development index (HDI) and matched the HDI with the GBD data.

### Statistical analysis

To study the trend in prevalence from 1990 to 2017, the age-standardized prevalence rate (ASR) and the estimated annual percentage change (EAPC) were used [1, 31]. The calculation of ASR and EAPC have been detailed in Liu et al. study [1]. By analyzing the ASR, we determined the prevalence of the disease and changes in etiology. Moreover, the ASR provided a theoretical basis for establishing targeted preventive strategies for public health departments [32]. In our study, EAPC was used to assess the trend in the ASR over a period of time [32, 33]. If ASR was a trend of increase, the value of EAPC and the lower boundary of 95% confidence interval (CI) are both greater than 0. In contrast, if ASR was a trend of decrease, the value of EAPC and the upper boundary of 95% CI are both less than 0. Additionally, if ASR was a constant trend, the 95% CI of EAPC contains 0. Additionally, a correlation analysis was conducted to study the influential factors for EAPC. All data were analyzed by R software (R 3.5.1 software, Institute for Statistics and Mathematics) and STATA (STATA 13.1, StataCorp LLC). A *p*-value less than 0.05 was considered statistically significant.

## Supplementary Material

Supplementary Figures

Supplementary Table 1

Supplementary Table 2

Supplementary Table 3

Supplementary Table 4

Supplementary Table 5

Supplementary Table 6

Supplementary Table 7
