# Supplementary material for: The burden of liver cirrhosis and underlying etiologies: results from the global burden of disease study 2017
Source: Aging (Albany NY). 2021 Jan 12;13(1):279–300. doi: 10.18632/aging.104127 (PMC7835066; doi:10.18632/aging.104127)
Supplement: Supplementary Figures [file aging-13-104127-s001.pdf]

SUPPLEMENTARY FIGURES

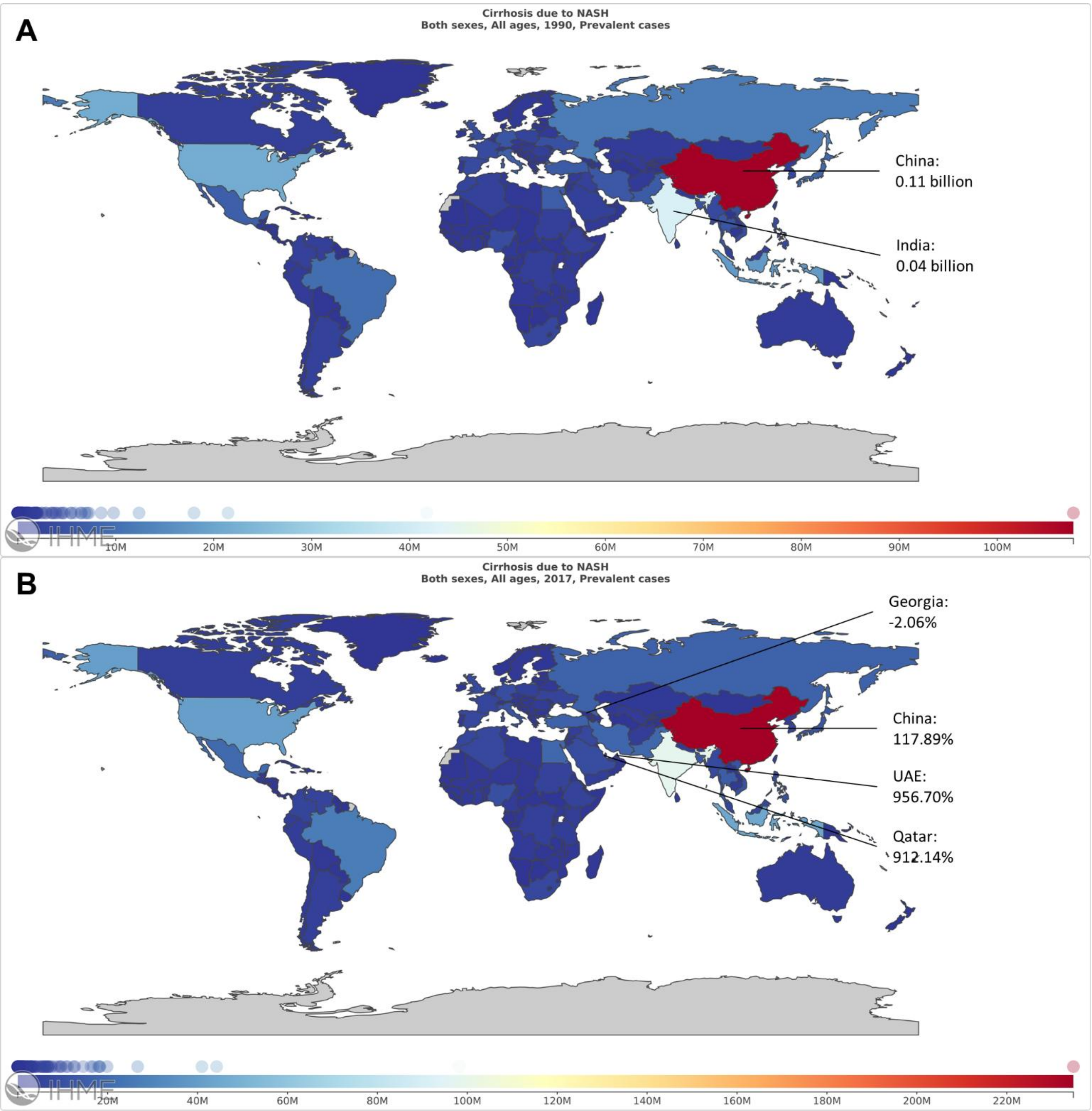

Supplementary Figure 1. The worldwide prevalence cases of liver cirrhosis caused by HBV in countries. (**A**) The worldwide prevalence cases of liver cirrhosis caused by HBV in 1990. (**B**) The worldwide prevalence cases of liver cirrhosis caused by HBV in 2017.

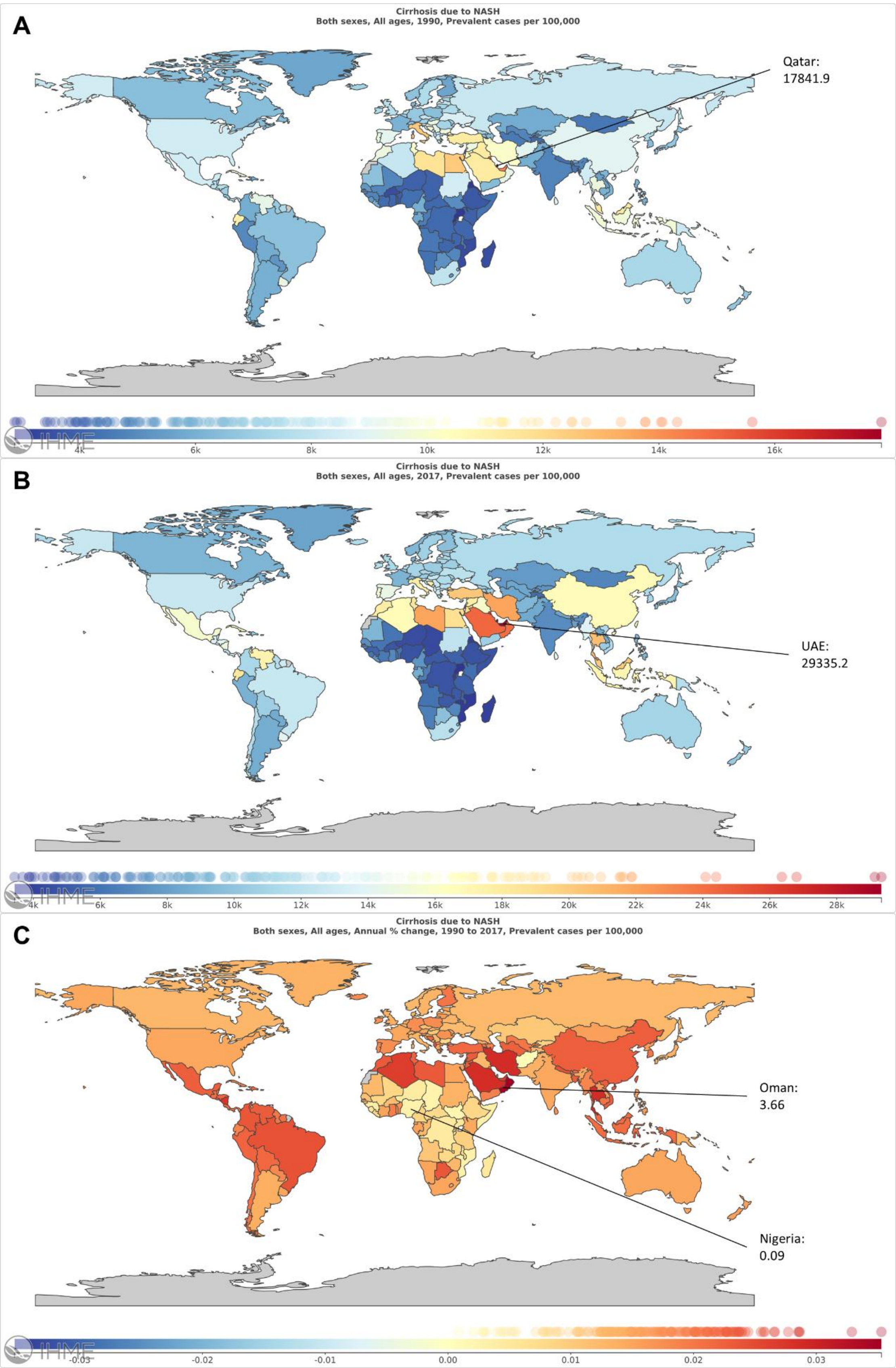

Supplementary Figure 2. The global burden of liver cirrhosis caused by HBV in countries. (**A**) The ASR of liver cirrhosis caused by HBV in 1990. (**B**) The ASR of liver cirrhosis caused by HBV in 2017. (**C**) The EAPC of liver cirrhosis caused by HBV from 1990 to 2017.

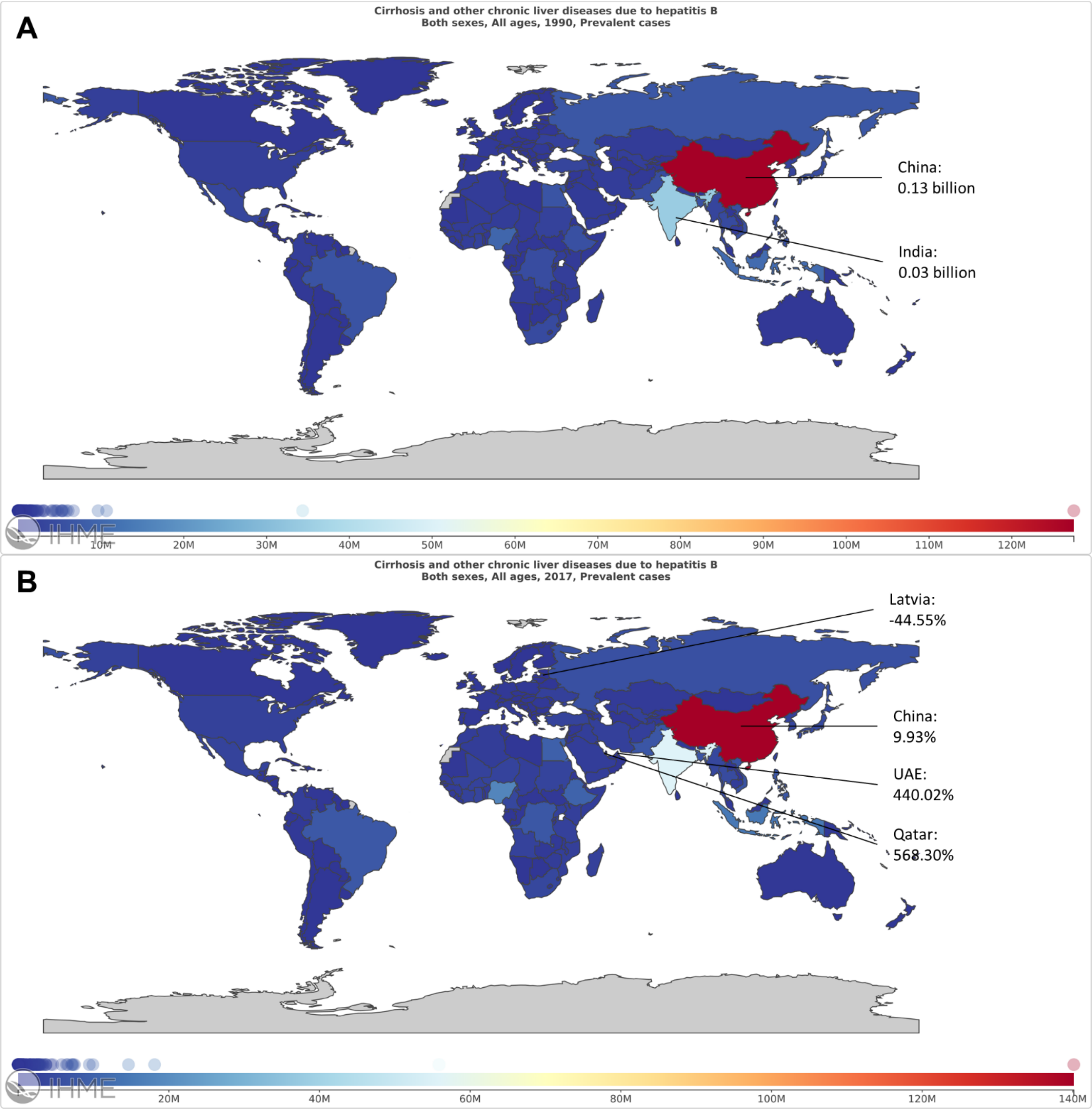

Supplementary Figure 3. The worldwide prevalence cases of liver cirrhosis caused by HCV in countries. (**A**) The worldwide prevalence cases of liver cirrhosis caused by HCV in 1990. (**B**) The worldwide prevalence cases of liver cirrhosis caused by HCV in 2017.

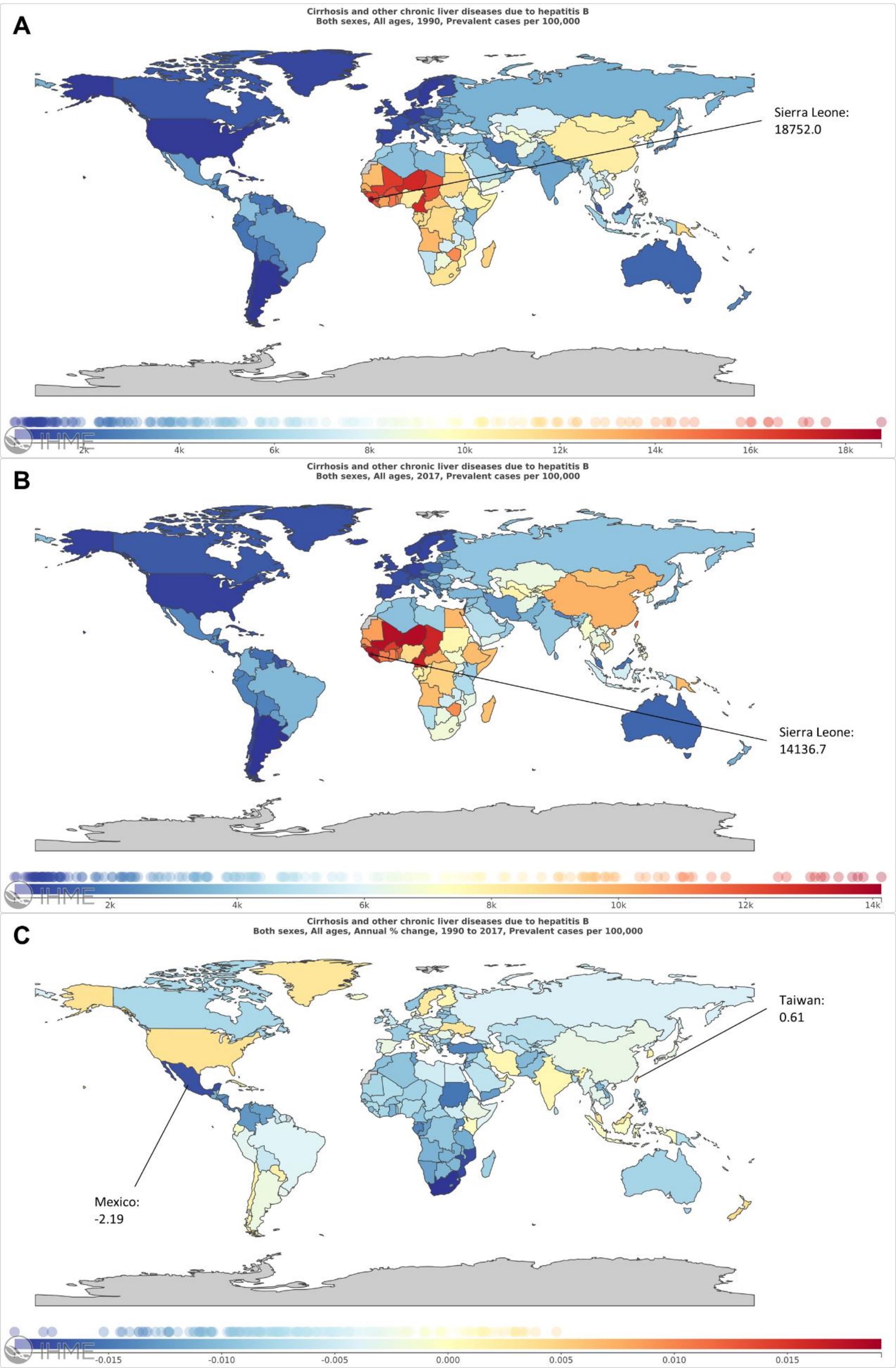

Supplementary Figure 4. The global burden of liver cirrhosis caused by HCV in countries. (**A**) The ASR of liver cirrhosis caused by HCV in 1990. (**B**) The ASR of liver cirrhosis caused by HCV in 2017. (**C**) The EAPC of liver cirrhosis caused by HCV from 1990 to 2017.

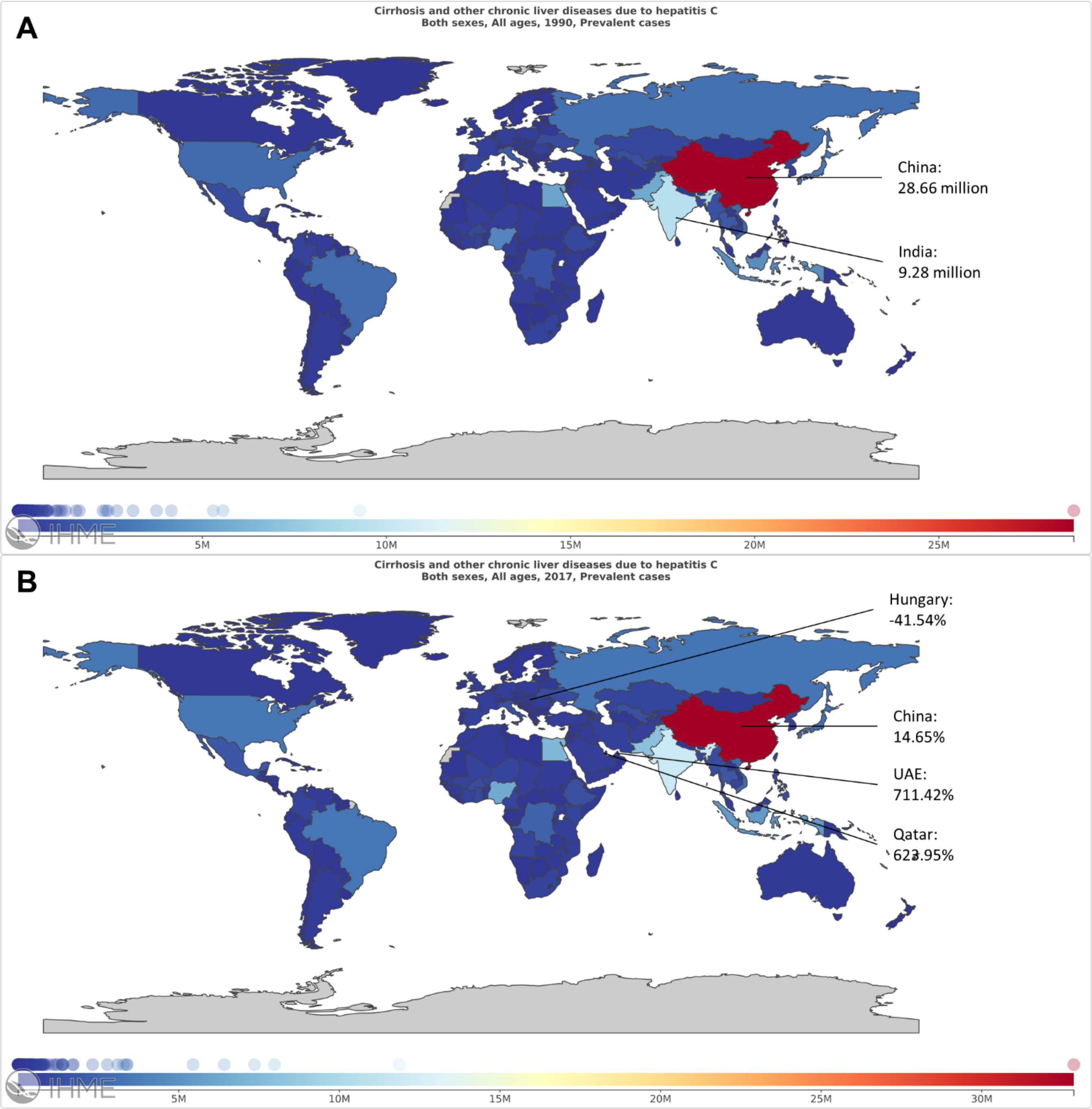

Supplementary Figure 5. The worldwide prevalence cases of liver cirrhosis caused by alcohol use in countries. (**A**) The worldwide prevalence cases of liver cirrhosis caused by alcohol use in 1990. (**B**) The worldwide prevalence cases of liver cirrhosis caused by alcohol use in 2017.

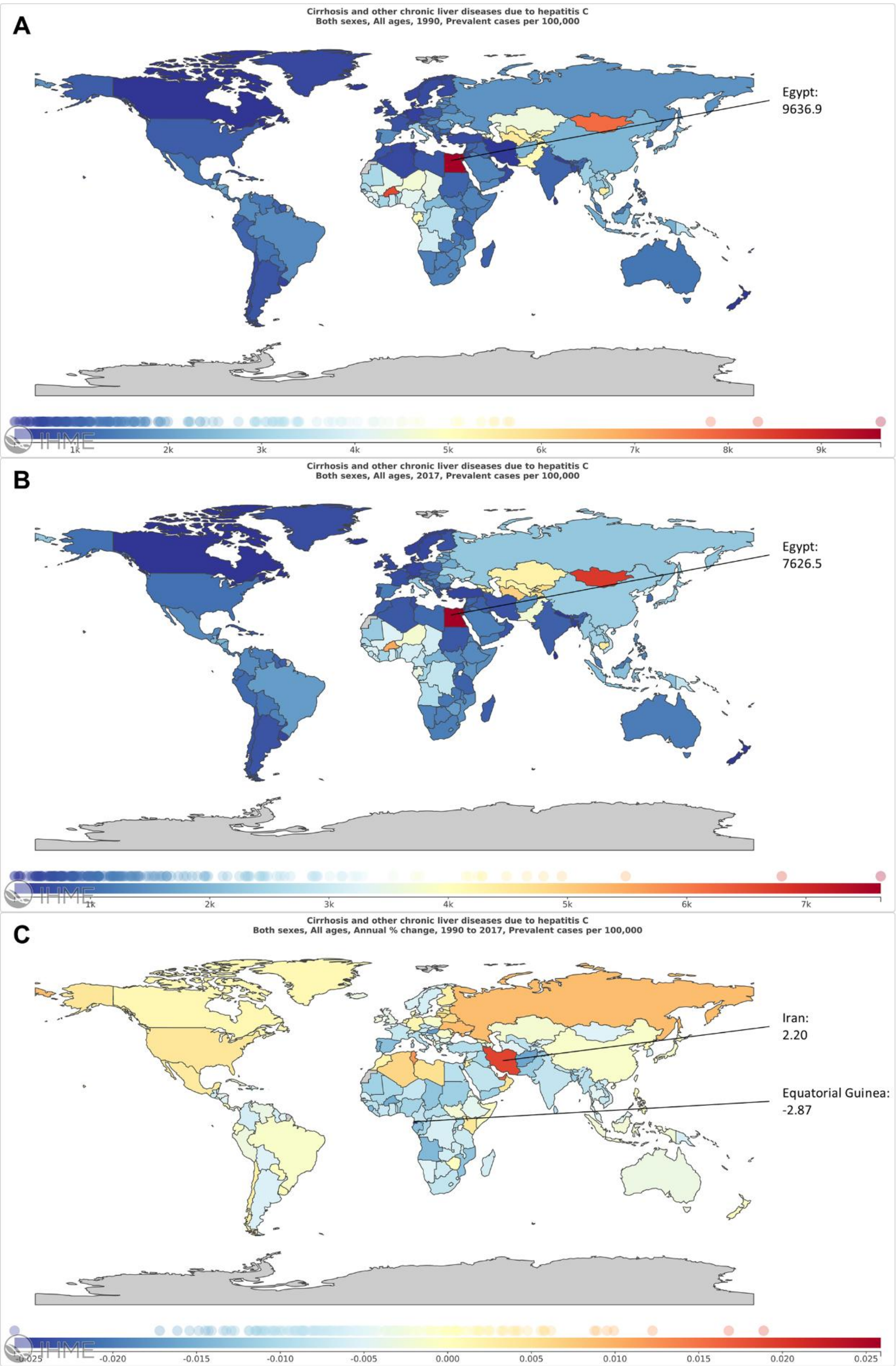

Supplementary Figure 6. The global burden of liver cirrhosis caused by alcohol use in countries. (**A**) The ASR of liver cirrhosis caused by alcohol use in 1990. (**B**) The ASR of liver cirrhosis caused by alcohol use in 2017. (**C**) The EAPC of liver cirrhosis caused by alcohol use from 1990 to 2017.

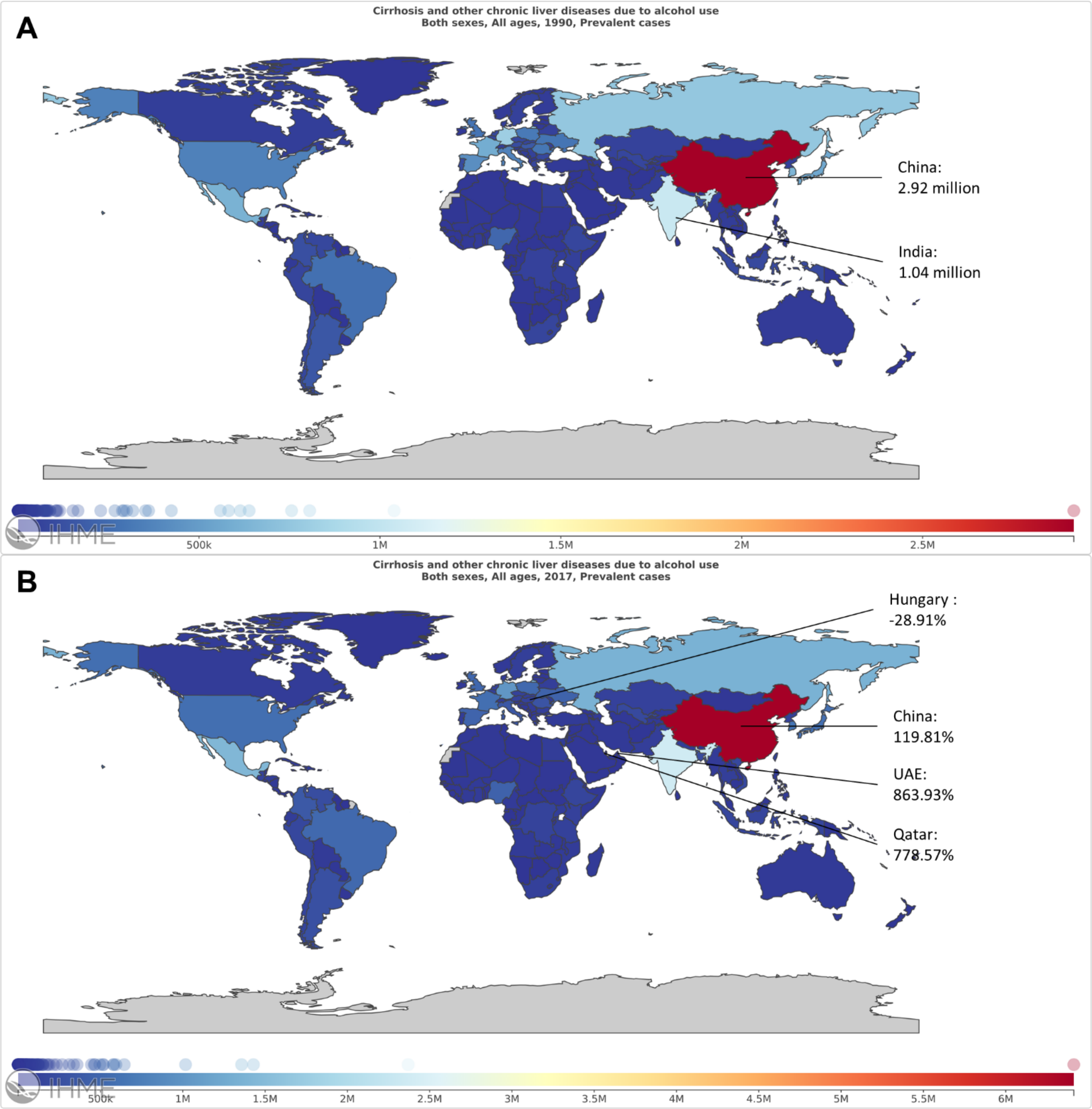

Supplementary Figure 7. The worldwide prevalence cases of liver cirrhosis caused by NASH in countries. (**A**) The worldwide prevalence cases of liver cirrhosis caused by NASH in 1990. (**B**) The worldwide prevalence cases of liver cirrhosis caused by NASH in 2017.

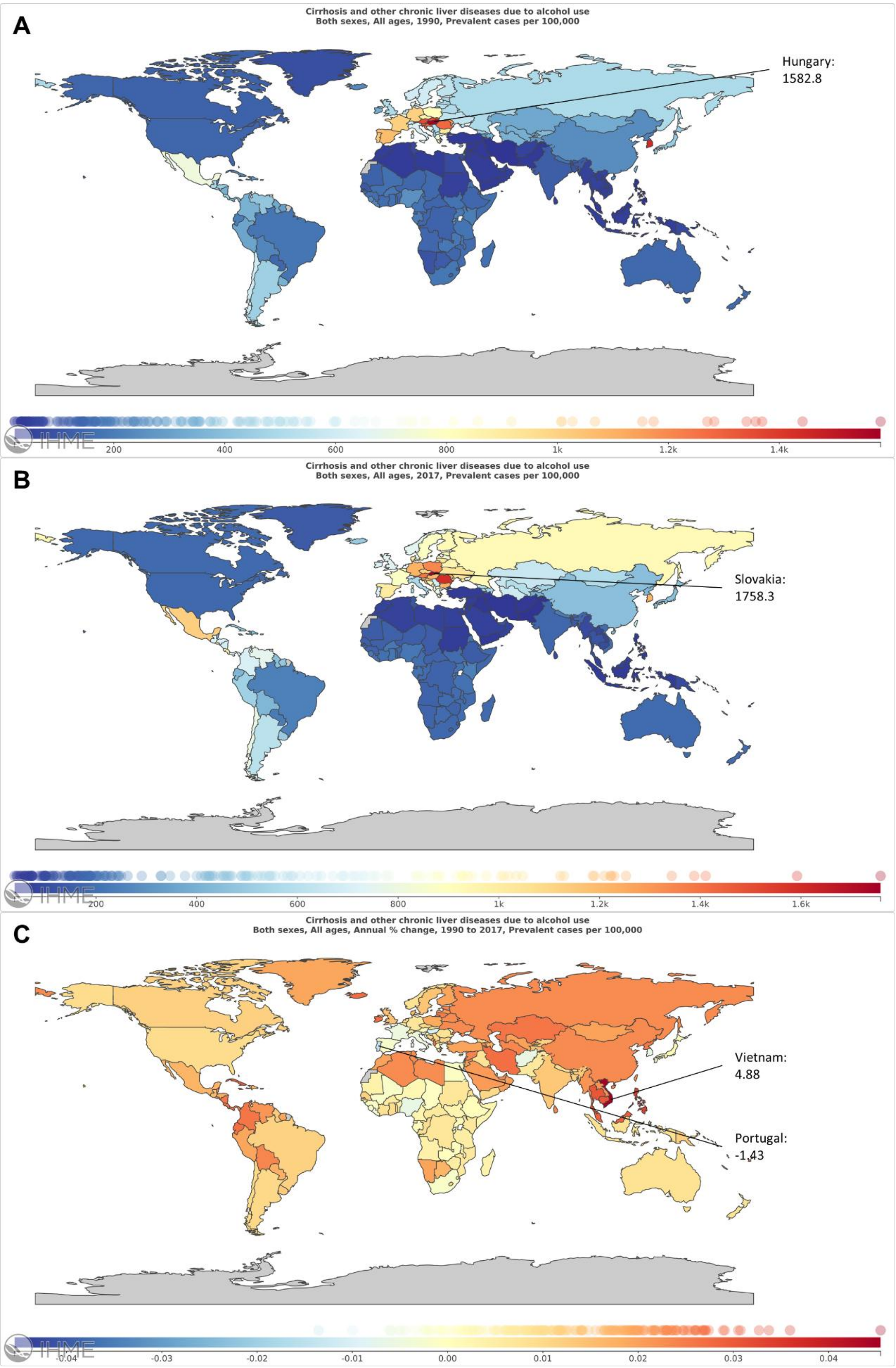

Supplementary Figure 8. The global burden of liver cirrhosis caused by NASH in countries. (**A**) The ASR of liver cirrhosis caused by NASH in 1990. (**B**) The ASR of liver cirrhosis caused by NASH in 2017. (**C**) The EAPC of liver cirrhosis caused by NASH from 1990 to 2017.

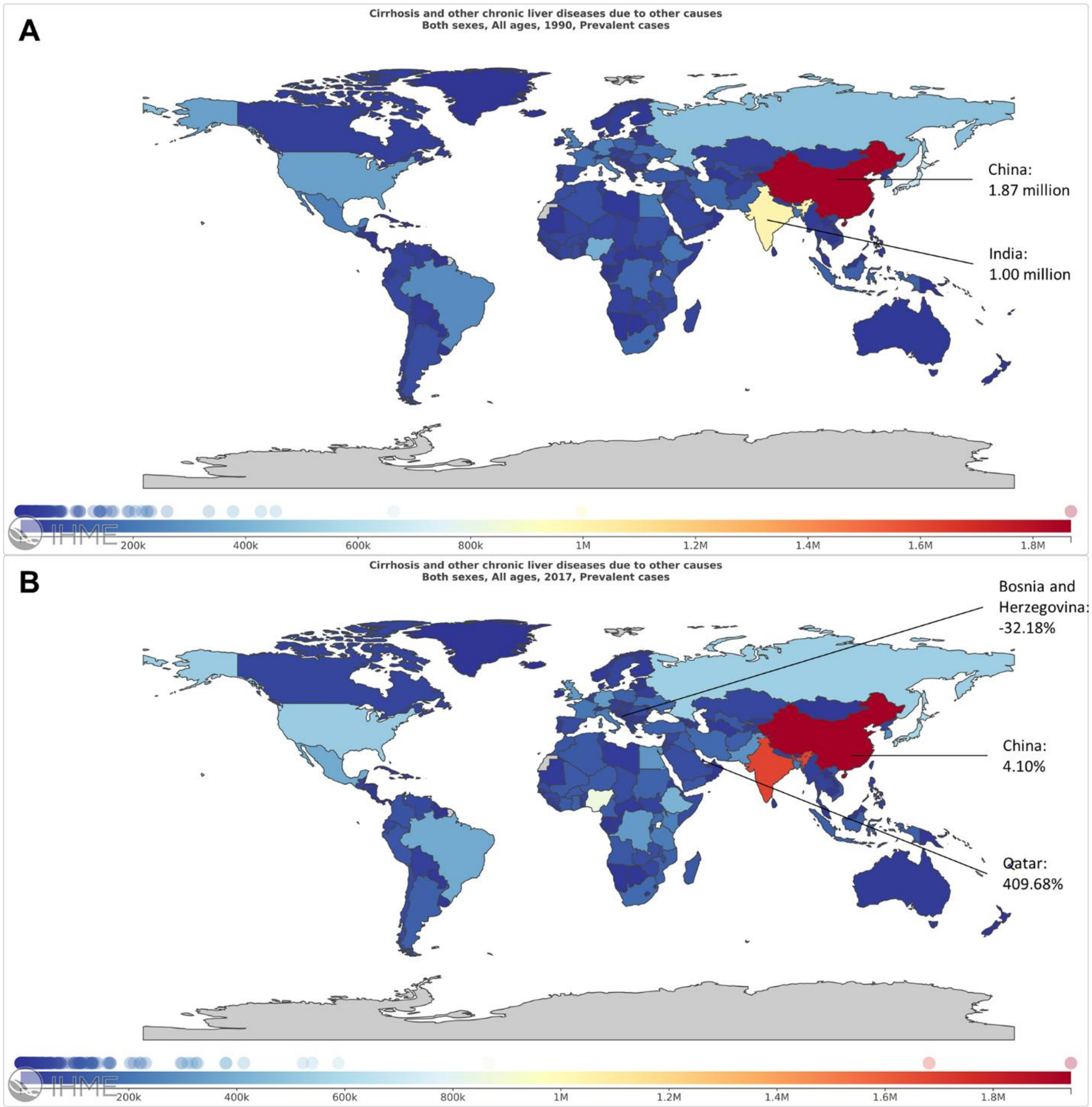

Supplementary Figure 9. The worldwide prevalence cases of liver cirrhosis caused by other causes in countries. (**A**) The worldwide prevalence cases of liver cirrhosis caused by other causes in 1990. (**B**) The worldwide prevalence cases of liver cirrhosis caused by other causes in 2017.

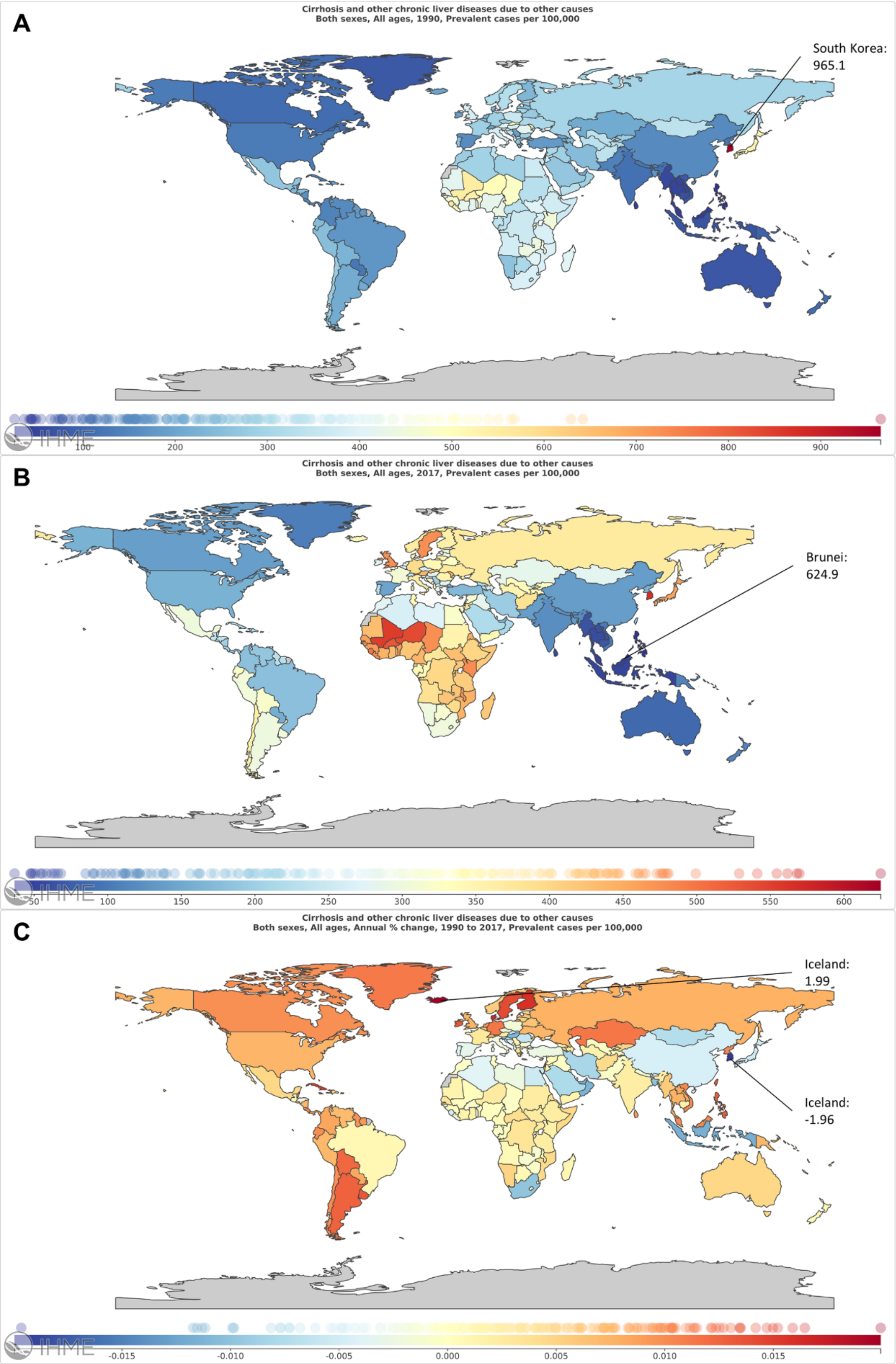

Supplementary Figure 10. The global burden of liver cirrhosis caused by other causes in countries. (**A**) The ASR of liver cirrhosis caused by other causes in 1990. (**B**) The ASR of liver cirrhosis caused by other causes in 2017. (**C**) The EAPC of liver cirrhosis caused by other causes from 1990 to 2017.
