# Supplementary material for: The burden of liver cirrhosis and underlying etiologies: results from the global burden of disease study 2017
Source: Aging (Albany NY). 2021 Jan 12;13(1):279–300. doi: 10.18632/aging.104127 (PMC7835066; doi:10.18632/aging.104127)
Supplement: Supplementary Table 1 [file aging-13-104127-s002.docx]

**Supplemental Table 1. The prevalence cases, age-standardized prevalence, and temporal trend of liver cirrhosis caused by NASH.**

| Characteristics | 1990 | |  | 2017 | |  | 1990–2017 |
| --- | --- | --- | --- | --- | --- | --- | --- |
|  | Prevalence cases No. ×10^3^ (95% UI) | ASR per 100,000 No. (95% UI) |  | Prevalence cases No. ×10^3^ (95% UI) | ASR per 100,000 No. (95% UI) |  | EAPC No. (95% CI) |
| Overall | 395517.8(379947.3-411747.6) | 7331.6(7043.0-7632.4) |  | 892322.8(858624.9-927954.4) | 11678.9(11237.9-12145.3) |  | 1.74(1.73-1.75) |
| Sex |  |  |  |  |  |  |  |
| Male | 238780.9(229292.9-248544.0) | 8786.7(8437.6-9146.0) |  | 529794.5(509933.1-550960.0) | 13816.5(13298.5-14368.5) |  | 1.69(1.69-1.70) |
| Female | 156736.9(150553.8-163266.7) | 5854.6(5623.6-6098.5) |  | 362528.3(348459.6-377017.9) | 9525.3(9155.6-9906.0) |  | 1.82(1.80-1.83) |
| Socio-demographic index |  |  |  |  |  |  |  |
| Low | 30697.2(29487.5-32013.0) | 4401.0(4227.6-4589.7) |  | 76936.8(73612.2-80490.1) | 5964.5(5706.8-6240.0) |  | 1.15(1.06-1.24) |
| Low-middle | 62964.0(60305.6-65690.0) | 6029.9(5775.3-6290.9) |  | 158757.4(152382.5-165361.3) | 9312.8(8938.9-9700.2) |  | 1.63(1.61-1.66) |
| Middle | 121124.5(116327.0-125974.5) | 7808.8(7499.5-8121.5) |  | 285445.7(274452.1-296946.3) | 13656.1(13130.1-14206.3) |  | 2.08(2.06-2.10) |
| Middle-high | 101417.8(97590.7-105568.1) | 9120.3(8776.1-9493.5) |  | 227516.8(219053.1-236317.9) | 16399.3(15789.3-17033.7) |  | 2.19(2.17-2.20) |
| High | 77185.3(74126.8-80480.2) | 7990.5(7673.9-8331.6) |  | 139104.6(133874.2-144820.7) | 12204.1(11745.3-12705.6) |  | 1.61(1.53-1.70) |
| Region |  |  |  |  |  |  |  |
| Asia Pacific–high income | 11619.5(11174.0-12091.3) | 6694.8(6438.1-6966.7) |  | 18994.9(18229.6-19845.6) | 10155.8(9746.6-10610.6) |  | 1.57(1.51-1.64) |
| Central Asia | 3851.0(3647.0-4075.7) | 5520.7(5228.2-5842.8) |  | 7536.2(7133.8-7981.8) | 8288.3(7845.8-8778.4) |  | 1.62(1.52-1.72) |
| East Asia | 112994.5(108297.2-117791.1) | 8977.4(8604.2-9358.5) |  | 245697.3(236288.9-255787.8) | 16537.3(15904.1-17216.5) |  | 2.22(2.18-2.26) |
| South Asia | 53810.4(51956.0-55724.3) | 4853.2(4685.9-5025.8) |  | 131737.1(127118.4-136528.9) | 7389.8(7130.8-7658.6) |  | 1.60(1.53-1.66) |
| Southeast Asia | 39616.3(38052.0-41321.2) | 8487.5(8152.4-8852.8) |  | 98943.6(95120.6-103082.6) | 14980.5(14401.7-15607.1) |  | 2.21(2.17-2.24) |
| Australasia | 1467.2(1400.6-1535.9) | 7238.1(6909.4-7576.8) |  | 3081.0(2958.6-3209.3) | 10851.7(10420.9-11303.7) |  | 1.58(1.45-1.71) |
| Caribbean | 2855.3(2707.9-3013.8) | 8084.9(7667.6-8533.7) |  | 5964.6(5669.1-6282.9) | 12892.1(12253.4-13580.2) |  | 1.87(1.83-1.92) |
| Central Europe | 9653.0(9256.5-10090.5) | 7776.6(7457.2-8129.1) |  | 13357.9(12818.8-13904.0) | 11635.4(11165.9-12111.1) |  | 1.55(1.53-1.58) |
| Eastern Europe | 18665.9(17932.4-19504.9) | 8225.4(7902.2-8595.1) |  | 24268.2(23295.1-25343.6) | 11545.3(11082.4-12056.9) |  | 1.33(1.29-1.38) |
| Western Europe | 31335.1(29961.4-32819.2) | 8124.7(7768.5-8509.5) |  | 52886.0(50746.6-55153.6) | 12214.7(11720.6-12738.4) |  | 1.53(1.40-1.67) |
| Andean Latin America | 2552.8(2439.0-2673.1) | 6655.0(6358.3-6968.5) |  | 7231.2(6975.2-7515.6) | 11767.9(11351.3-12230.7) |  | 2.19(2.14-2.24) |
| Central Latin America | 12970.8(12400.6-13573.8) | 7902.1(7554.7-8269.4) |  | 37394.0(35957.5-39016.7) | 14636.2(14074.0-15271.4) |  | 2.33(2.29-2.36) |
| Southern Latin America | 3161.7(3032.2-3299.2) | 6380.8(6119.5-6658.3) |  | 6425.3(6191.1-6690.3) | 9793.3(9436.4-10197.3) |  | 1.63(1.60-1.65) |
| Tropical Latin America | 9995.8(9493.1-10514.8) | 6514.0(6186.3-6852.2) |  | 27263.9(26145.4-28494.7) | 12463.9(11952.5-13026.5) |  | 2.39(2.37-2.40) |
| North Africa and Middle East | 35412.1(33899.4-37076.0) | 10387.7(9943.9-10875.8) |  | 109357.2(104821.1-113928.5) | 18220.7(17464.9-18982.3) |  | 2.16(2.12-2.19) |
| North America–high income | 23231.7(22282.4-24266.4) | 8275.8(7937.6-8644.4) |  | 44270.7(42584.0-46171.5) | 12267.3(11799.9-12794.0) |  | 1.53(1.45-1.62) |
| Oceania | 586.5(547.3-626.9) | 9083.4(8475.8-9708.3) |  | 1618.9(1512.8-1728.5) | 12845.5(12004.1-13715.3) |  | 1.33(1.31-1.35) |
| Central Sub-Saharan Africa | 2331.1(2183.1-2484.2) | 4236.6(3967.5-4514.8) |  | 6262.7(5825.1-6744.7) | 5147.3(4787.6-5543.4) |  | 0.74(0.64-0.83) |
| Eastern Sub-Saharan Africa | 7179.8(6817.3-7576.5) | 3748.0(3558.8-3955.1) |  | 18278.0(17309.0-19324.3) | 4648.8(4402.3-4914.9) |  | 0.79(0.71-0.86) |
| Southern Sub-Saharan Africa | 3717.5(3544.2-3909.4) | 7083.5(6753.3-7449.1) |  | 8210.6(7797.3-8650.5) | 10611.6(10077.5-11180.1) |  | 1.48(1.43-1.53) |
| Western Sub-Saharan Africa | 8509.6(8053.0-8960.3) | 4426.7(4189.1-4661.1) |  | 23543.6(22347.6-24804.1) | 5427.1(5151.4-5717.7) |  | 6.62(6.04-7.21) |
