# Supplementary material for: The burden of liver cirrhosis and underlying etiologies: results from the global burden of disease study 2017
Source: Aging (Albany NY). 2021 Jan 12;13(1):279–300. doi: 10.18632/aging.104127 (PMC7835066; doi:10.18632/aging.104127)
Supplement: Supplementary Table 2 [file aging-13-104127-s003.docx]

**Supplemental Table 2. The prevalence cases, age-standardized prevalence, and temporal trend of liver cirrhosis caused by HBV.**

| Characteristics | 1990 | |  | 2017 | |  | 1990–2017 |
| --- | --- | --- | --- | --- | --- | --- | --- |
|  | Prevalence cases No. ×10^3^ (95% UI) | ASR per 100,000 No. (95% UI) |  | Prevalence cases No. ×10^3^ (95% UI) | ASR per 100,000 No. (95% UI) |  | EAPC No. (95% CI) |
| Overall | 333780.7(302793.8-363301.0) | 6187.2(5612.8-6734.4) |  | 431116.3(395729.0-468718.9) | 5642.5(5179.4-6134.7) |  | -0.39(-0.46--0.34) |
| Sex |  |  |  |  |  |  |  |
| Male | 192320.7(174394.7-209162.4) | 7077.0(6417.4-7696.8) |  | 249020.2(229452.0-270899.7) | 6494.2(5983.9-7064.8) |  | -0.35(-0.40--0.29) |
| Female | 141460.0(128848.2-153923.4) | 5283.9(4812.8-5749.5) |  | 182096.1(166283.0-198589.0) | 4784.5(4369.0-5217.8) |  | -0.46(-0.53--0.40) |
| Socio-demographic index |  |  |  |  |  |  |  |
| Low | 50098.4(44256.8-556589.0 | 7182.6(6345.1-7979.8) |  | 84518.8(74380.2-94120.1) | 6552.3(5766.3-7296.7) |  | -0.40(-0.48--0.33) |
| Low-middle | 68108.3(61056.6-74366.7) | 6522.5(5847.2-7121.9) |  | 100967.9(90147.6-109822.9) | 5922.9(5288.1-6442.3) |  | -0.48(-0.55--0.42) |
| Middle | 1171367(106087.3-127396.1) | 7551.7(6839.4-8213.2) |  | 132943.0(122382.2-144656.1) | 6360.2(5854.9-6920.5) |  | -0.63(-0.71--0.55) |
| Middle-high | 75321.9(68633.1-81397.3) | 6773.6(6172.0-7319.9) |  | 86033.6(79185.8-94032.7) | 6201.3(5707.7-6777.9) |  | -0.38(-0.48--0.27) |
| High | 20757.2(19531.7-22168.9) | 2148.9(2022.0-2295.0) |  | 24086.9(22790.0-25617.4) | 2113.2(1999.4-2247.5) |  | -0.10(-0.13--0.06) |
| Region |  |  |  |  |  |  |  |
| Asia Pacific–high income | 7327.9(6851.5-7889.6) | 4222.1(3947.6-4545.8) |  | 7834.1(7419.3-8318.6) | 4188.6(3966.8-4447.6) |  | -0.12(-0.17--0.08) |
| Central Asia | 5680.9(5210.3-6215.3) | 8143.9(7469.3-8910.0) |  | 6387.4(5957.0-6929.8) | 7024.8(6551.5-7621.5) |  | -0.75(-0.83--0.67) |
| East Asia | 133819.0(119541.8-145328.8) | 10632.0(9497.6-11546.4) |  | 147573.8(135463.1-161697.7) | 9932.9(9117.7-10883.5) |  | -0.26(-0.39--0.13) |
| South Asia | 45154.7(40558.1-50123.8) | 4072.5(3657.9-4520.7) |  | 70394.3(63518.4-77835.3) | 3948.8(3563.1-4366.2) |  | -0.09(-0.12--0.06) |
| Southeast Asia | 30841.8(27238.8-33944.0) | 6607.6(5835.7-7272.3) |  | 39868.7(36158.4-43112.5) | 6036.3(5474.5-6527.4) |  | -0.34(-0.40--0.28) |
| Australasia | 428.1(391.9-469.2) | 2111.7(1933.4-2314.8) |  | 520.9(482.0-562.2) | 1834.8(1697.6-1980.3) |  | -0.68(-0.87--0.49) |
| Caribbean | 406.2(371.0-450.1) | 1150.2(1050.6-1274.6) |  | 516.5(476.2-562.4) | 1116.5(1029.4-1215.6) |  | -0.11(-0.14--0.08) |
| Central Europe | 3529.5(3325.5-3763.4) | 2843.5(2679.1-3031.9) |  | 2887.0(2743.8-3061.8) | 2514.7(2390.0-2667.0) |  | -0.40(-0.50--0.30) |
| Eastern Europe | 9997.3(9052.2-10990.4) | 4405.5(3989.0-4843.1) |  | 8593.3(7860.5-9395.8) | 4088.1(3739.6-4470.0) |  | -0.38(-0.48--0.27) |
| Western Europe | 4670.3(4384.6-5059.9) | 1210.9(1136.9-1311.9) |  | 4629.4(4359.3-4969.7) | 1069.2(1006.8-1147.8) |  | -0.36(-0.39--0.33) |
| Andean Latin America | 914.3(828.8-1011.2) | 2383.5(2160.7-2636.1) |  | 1346.4(1227.0-1466.1) | 2191.1(1996.8-2385.9) |  | -0.47(-0.54--0.40) |
| Central Latin America | 6080.7(5443.4-6840.0) | 3704.5(3316.2-4167.0) |  | 6293.4(5715.6-6909.6) | 2463.3(2237.1-2704.4) |  | -1.73(-2.00--1.47) |
| Southern Latin America | 310.9(289.7-334.4) | 627.5(584.7-674.8) |  | 402.9(381.8-433.2) | 614.1(581.9-660.2) |  | -0.18(-0.27--0.09) |
| Tropical Latin America | 5939.9(5355.4-6568.9) | 3870.8(3489.9-4280.8) |  | 7682.6(6958.0-8429.8) | 3512.1(3180.9-3853.7) |  | -0.44(-0.48--0.40) |
| North Africa and Middle East | 21418.5(19405.9-23335.8) | 6282.8(5692.5-6845.2) |  | 31404.3(28019.5-33966.2) | 5232.5(4668.5-5659.3) |  | -0.70(-0.75--0.65) |
| North America–high income | 2165.5(1976.9-2393.0) | 771.4(704.2-852.4) |  | 2903.7(2665.8-3166.9) | 804.6(738.7-877.5) |  | -0.02(-0.09--0.05) |
| Oceania | 705.5(607.5-786.1) | 10925.6(9407.7-12173.8) |  | 1191.5(932.0-1343.1) | 9454.6(7395.5-10657.4) |  | -0.47(-0.56--0.38) |
| Central Sub-Saharan Africa | 6539.4(5590.8-7327.9) | 11884.6(10160.6-13317.6) |  | 11152.1(8186.9-12869.6) | 9165.9(6728.8-10577.5) |  | -1.15(-1.35--0.94) |
| Eastern Sub-Saharan Africa | 16273.0(14553.4-18043.7) | 8494.8(7597.2-9419.2) |  | 27536.8(24500.8-30609.0) | 7003.6(6231.5-7785.0) |  | -0.83(-0.92--0.75) |
| Southern Sub-Saharan Africa | 6080.7(5304.2-6814.0) | 11586.4(10106.7-12983.7) |  | 5745.4(5088.9-6394.7) | 7425.4(6577.0-8264.7) |  | -1.47(-1.58--1.37) |
| Western Sub-Saharan Africa | 25496.8(20396.9-28072.3) | 13263.3(10610.4-14603.0) |  | 46251.7(37240.9-50760.0) | 10661.6(8584.5-11700.8) |  | -1.06(-1.18--0.93) |
