# Supplementary material for: The burden of liver cirrhosis and underlying etiologies: results from the global burden of disease study 2017
Source: Aging (Albany NY). 2021 Jan 12;13(1):279–300. doi: 10.18632/aging.104127 (PMC7835066; doi:10.18632/aging.104127)
Supplement: Supplementary Table 3 [file aging-13-104127-s004.docx]

**Supplemental Table 3. The prevalence cases, age-standardized prevalence, and temporal trend of liver cirrhosis caused by HCV.**

| Characteristics | 1990 | |  | 2017 | |  | 1990–2017 |
| --- | --- | --- | --- | --- | --- | --- | --- |
|  | Prevalence cases No. ×10^3^ (95% UI) | ASR per 100,000 No. (95% UI) |  | Prevalence cases No. ×10^3^ (95% UI) | ASR per 100,000 No. (95% UI) |  | EAPC No. (95% CI) |
| Overall | 104466.6(92754.6-118648.8) | 1936.5(1719.4-2199.4) |  | 134493.9(118558.2-153823.8) | 1760.3(1551.7-2013.3) |  | -0.39(-0.42--0.36) |
| Sex |  |  |  |  |  |  |  |
| Male | 51793.0(45985.5-58752.8) | 1905.9(1692.2-2162.0) |  | 67694.5(59790.1-77050.7) | 1765.4(1559.3-2009.4) |  | -0.36(-0.39--0.33) |
| Female | 52673.6(46577.1-59936.6) | 1967.5(1739.8-2238.8) |  | 66799.4(58752.0-76683.5) | 1755.1(1543.7-2014.8) |  | -0.42(-0.46--0.39) |
| Socio-demographic index |  |  |  |  |  |  |  |
| Low | 11254.4(9806.4-13081.5) | 1613.5(1405.9-1875.5) |  | 17828.9(15504.5-20702.7) | 1382.2(1202.0-1605.0) |  | -0.50(-0.53--0.48) |
| Low-middle | 28164.8(24697.5-32342.0) | 2697.3(2365.2-3097.3) |  | 39583.9(34602.7-45425.1) | 2322.0(2029.8-2664.7) |  | -0.60(-0.67--0.54) |
| Middle | 30637.4(27038.3-34958.2) | 1975.2(1743.1-2253.7) |  | 36903.4(32455.9-42384.6) | 1765.5(1552.7-2027.7) |  | -0.48(-0.52--0.44) |
| Middle-high | 21390.9(18988.1-24273.9) | 1923.6(1707.6-2182.9) |  | 25862.2(22860.7-29435.4) | 1864.1(1647.8-2121.7) |  | -0.16(-0.22--0.09) |
| High | 12463.1(11400.9-13770.3) | 1290.2(1180.3-1425.6) |  | 13684.7(12487.9-15209.6) | 1200.6(1095.6-1334.4) |  | -0.28(-0.33--0.23) |
| Region |  |  |  |  |  |  |  |
| Asia Pacific–high income | 3663.0(3346.4-4052.2) | 2110.5(1928.1-2334.7) |  | 3668.3(3362.0-4012.7) | 1961.3(1797.5-2145.4) |  | -0.06(-0.16--0.02) |
| Central Asia | 3535.4(3131.3-4012.1) | 5068.2(4488.8-5751.5) |  | 4059.4(3566.6-4646.3) | 4464.5(3922.5-5110.1) |  | -0.51(-0.62--0.39) |
| East Asia | 30482.1(27003.8-34543.2) | 2421.8(2145.5-2744.5) |  | 34980.9(30607.3-40167.1) | 2354.5(2060.1-2703.6) |  | -0.26(-0.33--0.19) |
| South Asia | 15685.3(13725.2-18038.1) | 1414.7(1237.9-1626.9) |  | 20753.4(18101.9-24186.7) | 1164.2(1015.4-1356.8) |  | -0.66(-0.77--0.54) |
| Southeast Asia | 11113.5(9651.3-12821.8) | 2381.0(2067.7-2747.0) |  | 13920.0(12130.3-16131.1) | 2107.5(1836.6-2442.3) |  | -0.41(-0.43--0.38) |
| Australasia | 234.1(207.4-268.0) | 1154.6(1023.1-1322.3) |  | 304.7(267.6-347.9) | 1073.0(942.5-1225.5) |  | -0.24(-0.29--0.18) |
| Caribbean | 326.0(284.6-377.4) | 923.1(805.8-1068.7) |  | 377.7(335.0-436.5) | 816.4(724.0-943.4) |  | -0.54(-0.58--0.50) |
| Central Europe | 1570.4(1404.3-1774.2) | 1265.1(1131.3-1429.4) |  | 1264.9(1134.0-1419.9) | 1101.8(987.8-1236.8) |  | -0.50(-0.58--0.41) |
| Eastern Europe | 4115.4(3605.6-4743.2) | 1813.5(1588.9-2090.1) |  | 4772.6(4220.3-5436.8) | 2270.5(2007.8-2586.5) |  | 1.05(0.81-1.29) |
| Western Europe | 4261.5(3887.7-4721.3) | 1104.9(1008.0-1224.2) |  | 3896.8(3542.1-4328.0) | 900.0(818.1-999.6) |  | -0.72(-0.80--0.64) |
| Andean Latin America | 417.6(358.9-488.1) | 1088.5(935.6-1272.5) |  | 621.3(540.4-723.1) | 1011.2(879.4-1176.8) |  | -0.29(-0.32--0.27) |
| Central Latin America | 2367.7(2103.3-2717.3) | 1442.4(1281.3-1655.5) |  | 3615.4(3304.4-4027.0) | 1415.1(1293.4-1576.2) |  | 0.00(-0.03-0.03) |
| Southern Latin America | 378.4(341.0-425.0) | 763.6(688.1-857.8) |  | 460.4(423.3-500.4) | 701.7(645.2-762.7) |  | -0.13(-0.21--0.06) |
| Tropical Latin America | 2494.7(2172.8-2895.1) | 1625.7(1416.0-1886.6) |  | 3461.3(3024.7-3981.7) | 1582.4(1382.7-1820.2) |  | 0.11(-0.10-0.33) |
| North Africa and Middle East | 7756.6(6809.7-8914.8) | 2275.3(1997.5-2615.1) |  | 11622.6(10399.3-13177.0) | 1936.5(1732.7-2195.5) |  | -0.82(-0.91--0.73) |
| North America–high income | 2479.6(2173.3-2828.0) | 883.3(774.2-1007.4) |  | 3531.6(3101.2-4016.3) | 978.6(859.3-1112.9) |  | 0.13(0.01-0.26) |
| Oceania | 172.1(149.7-197.6) | 2664.9(2319.1-3059.7) |  | 312.2(269.2-361.0) | 2477.4(2136.1-2864.2) |  | -0.18(-0.23--0.12) |
| Central Sub-Saharan Africa | 1895.1(1625.3-2191.9) | 3444.2(2953.8-3983.5) |  | 3358.7(2881.9-3891.8) | 2760.5(2368.6-3198.6) |  | -0.67(-0.81--0.54) |
| Eastern Sub-Saharan Africa | 2719.7(2346.7-3201.1) | 1419.8(1225.0-1671.1) |  | 4841.5(4187.5-5712.7) | 1231.4(1065.0-1452.9) |  | -0.59(-0.65--0.53) |
| Southern Sub-Saharan Africa | 806.9(699.9-940.6) | 1537.4(1333.6-1792.2) |  | 1007.4(879.2-1181.3) | 1302.0(1136.3-1526.8) |  | -0.59(-0.74--0.44) |
| Western Sub-Saharan Africa | 7991.7(6875.7-9294.0) | 4157.2(3576.7-4834.7) |  | 13662.8(11690.1-15840.9) | 3149.4(2694.7-3651.5) |  | -1.07(-1.12--1.03) |
