# Supplementary material for: The burden of liver cirrhosis and underlying etiologies: results from the global burden of disease study 2017
Source: Aging (Albany NY). 2021 Jan 12;13(1):279–300. doi: 10.18632/aging.104127 (PMC7835066; doi:10.18632/aging.104127)
Supplement: Supplementary Table 4 [file aging-13-104127-s005.docx]

**Supplemental Table 4. The prevalence cases, age-standardized prevalence, and temporal trend of liver cirrhosis caused by alcohol use.**

| Characteristics | 1990 | |  | 2017 | |  | 1990–2017 |
| --- | --- | --- | --- | --- | --- | --- | --- |
|  | Prevalence cases No. ×10^3^ (95% UI) | ASR per 100,000 No. (95% UI) |  | Prevalence cases No. ×10^3^ (95% UI) | ASR per 100,000 No. (95% UI) |  | EAPC No. (95% CI) |
| Overall | 14608.3(13711.3-15577.7) | 270.8(254.2-288.8) |  | 26041.9(24252.8-28011.2) | 340.8(317.4-366.6) |  | 0.84(0.79-0.89) |
| Sex |  |  |  |  |  |  |  |
| Male | 9386.8(8828.6-9992.8) | 345.4(324.9-367.7) |  | 16332.1(15213.7-17583.2) | 425.9(396.7-458.6) |  | 0.77(0.72-0.81) |
| Female | 5221.5(4884.7-5563.5) | 195.0(182.5-207.8) |  | 9709.8(9028.6-10449.5) | 255.1(237.2-274.6) |  | 0.97(0.91-1.04) |
| Socio-demographic index |  |  |  |  |  |  |  |
| Low | 898.2(824.6-983.0) | 128.8(118.2-140.9) |  | 2016.1(1836.8-2219.6) | 156.3(142.4-172.1) |  | 0.69(0.58-0.80) |
| Low-middle | 1440.6(1324.4-1564.9) | 138.0(126.8-149.9) |  | 3127.0(2857.1-3412.5) | 183.4(167.6-200.2) |  | 1.01(0.97-1.05) |
| Middle | 3044.5(2849.7-3256.4) | 196.3(183.7-210.0) |  | 7006.5(6505.0-7549.3) | 335.2(311.2-361.2) |  | 1.95(1.88-2.01) |
| Middle-high | 3758.9(3532.7-3993.0) | 338.0(317.7-359.1) |  | 6793.2(6331.3-7292.0) | 489.7(456.4-525.6) |  | 1.43(1.37-1.49) |
| High | 5407.6(5083.6-5726.8) | 559.8(526.3-592.9) |  | 6976.4(6544.6-7453.1) | 612.1(574.2-653.9) |  | 0.28(0.26-0.31) |
| Region |  |  |  |  |  |  |  |
| Asia Pacific–high income | 1206.2(1107.2-1310.9) | 695.0(637.9-755.3) |  | 1259.9(1158.8-1368.9) | 673.6(619.5-731.9) |  | -0.30(-0.40--0.20) |
| Central Asia | 238.5(224.0-254.2) | 342.0(321.1-364.4) |  | 523.4(485.4-561.0) | 575.6(533.9-617.0) |  | 2.43(2.23-2.63) |
| East Asia | 3084.7(2856.8-3325.5) | 245.1(227.0-264.2) |  | 6782.7(6243.9-7381.5) | 456.5(420.3-496.8) |  | 2.29(2.13-2.44) |
| South Asia | 1218.4(1118.1-1323.5) | 109.9(100.8-119.4) |  | 2736.3(2492.5-3001.2) | 153.5(139.8-168.4) |  | 1.17(1.05-1.29) |
| Southeast Asia | 213.0(190.9-237.3) | 45.6(40.9-50.8) |  | 592.8(524.7-666.5) | 89.8(79.4-100.9) |  | 2.51(2.44-2.58) |
| Australasia | 30.2(27.4-33.0) | 149.2(135.3-162.9) |  | 52.8(47.9-57.8) | 186.1(168.7-203.6) |  | 0.95(0.86-1.04) |
| Caribbean | 93.6(87.2-100.0) | 265.0(246.8-283.1) |  | 201.5(185.7-218.3) | 435.5(401.4-471.9) |  | 1.70(1.64-1.76) |
| Central Europe | 1221.3(1148.8-1294.9) | 983.9(925.5-1043.2) |  | 1459.3(1365.9-1561.9) | 1271.1(1189.8-1360.5) |  | 1.09(1.00-1.19) |
| Eastern Europe | 1194.2(1121.0-1273.0) | 526.2(494.0-561.0) |  | 2014.5(1887.9-2153.1) | 958.4(898.1-1024.3) |  | 2.65(2.47-2.82) |
| Western Europe | 3125.5(2951.8-3304.0) | 810.4(765.4-856.7) |  | 3800.5(3554.8-4052.6) | 877.8(821.0-936.0) |  | 0.24(0.22-0.27) |
| Andean Latin America | 102.8(93.7-111.9) | 268.1(244.4-291.7) |  | 292.3(264.9-320.9) | 475.7(431.1-522.2) |  | 2.17(2.08-2.27) |
| Central Latin America | 927.9(873.0-982.4) | 565.3(531.8-598.5) |  | 2385.1(2235.9-2546.5) | 933.6(875.1-996.7) |  | 1.71(1.64-1.77) |
| Southern Latin America | 241.7(229.3-256.5) | 487.9(462.8-517.6) |  | 425.1(397.3-451.5) | 648.0(605.6-688.1) |  | 1.15(1.07-1.23) |
| Tropical Latin America | 273.1(256.3-290.8) | 178.0(167.0-189.5) |  | 534.3(498.5-573.5) | 244.3(227.9-262.2) |  | 1.05(0.95-1.15) |
| North Africa and Middle East | 145.1(129.8-161.9) | 42.6(38.1-47.5) |  | 372.3(330.7-416.8) | 62.0(55.1-69.4) |  | 1.48(1.35-1.61) |
| North America–high income | 388.4(366.3-411.5) | 138.4(130.5-146.6) |  | 647.8(607.1-692.8) | 179.5(168.2-192.0) |  | 0.86(0.73-0.99) |
| Oceania | 4.4(4.0-4.8) | 67.8(61.7-74.1) |  | 12.6(11.4-13.8) | 99.8(90.2-109.8) |  | 1.52(1.43-1.61) |
| Central Sub-Saharan Africa | 76.8(69.2-85.4) | 139.6(125.7-155.1) |  | 201.6(179.8-226.0) | 165.7(147.8-185.7) |  | 0.67(0.58-0.76) |
| Eastern Sub-Saharan Africa | 337.4(306.2-371.2) | 176.1(159.8-193.8) |  | 748.4(673.9-833.7) | 190.3(171.4-212.1) |  | 0.19(0.10-0.28) |
| Southern Sub-Saharan Africa | 89.4(82.7-97.1) | 170.5(157.6-185.0) |  | 134.8(124.0-147.1) | 174.3(160.2-190.1) |  | 0.02(-0.03-0.06) |
| Western Sub-Saharan Africa | 395.3(360.2-434.0) | 205.6(187.4-225.8) |  | 863.8(779.9-957.8) | 199.1(179.8-220.8) |  | -0.20(-0.24--0.16) |
