# Supplementary material for: The burden of liver cirrhosis and underlying etiologies: results from the global burden of disease study 2017
Source: Aging (Albany NY). 2021 Jan 12;13(1):279–300. doi: 10.18632/aging.104127 (PMC7835066; doi:10.18632/aging.104127)
Supplement: Supplementary Table 5 [file aging-13-104127-s006.docx]

**Supplemental Table 5. The prevalence cases, age-standardized prevalence, and temporal trend of liver cirrhosis caused by other causes.**

| Characteristics | 1990 | |  | 2017 | |  | 1990–2017 |
| --- | --- | --- | --- | --- | --- | --- | --- |
|  | Prevalence cases No. ×10^3^ (95% UI) | ASR per 100,000 No. (95% UI) |  | Prevalence cases No. ×10^3^ (95% UI) | ASR per 100,000 No. (95% UI) |  | EAPC No. (95% CI) |
| Overall | 11435.6(10525.0-12332.8) | 212.0(195.1-228.6) |  | 16616.0(15165.6-17954.6) | 217.5(198.5-235.0) |  | 0.04(-0.02-0.10) |
| Sex |  |  |  |  |  |  |  |
| Male | 4840.9(4430.5-5250.1) | 178.1(163.0-193.2) |  | 6997.9(6383.7-7598.8) | 182.5(166.5-198.2) |  | 0.04(0.00-0.08) |
| Female | 6594.6(6089.8-7079.5) | 246.3(227.5-264.4) |  | 9618.1(8798.5-10383.4) | 252.7(231.2-272.8) |  | 0.03(-0.04-0.11) |
| Socio-demographic index |  |  |  |  |  |  |  |
| Low | 1737.6(1577.0-1887.3) | 249.1(226.1-270.6) |  | 3584.2(3225.6-3920.1) | 277.9(250.1-303.9) |  | 0.37(0.30-0.44) |
| Low-middle | 2099.5(1914.8-2276.5) | 201.1(183.4-218.0) |  | 3807.8(3442.7-4154.0) | 223.4(202.0-243.7) |  | 0.36(0.33-0.39) |
| Middle | 2311.2(2119.7-2488.2) | 149.0(136.7-160.4) |  | 3103.3(2851.8-3351.5) | 148.5(136.4-160.3) |  | -0.13(-0.21--0.05) |
| Middle-high | 2333.4(2141.9-2512.7) | 209.8(192.6-226.0) |  | 2768.8(2540.6-2988.0) | 199.6(183.1-215.4) |  | -0.26(-0.31--0.21) |
| High | 2916.3(2680.9-3154.8) | 301.9(277.5-326.6) |  | 3309.6(3042.5-3589.5) | 290.4(266.9-314.9) |  | -0.20(-0.26--0.13) |
| Region |  |  |  |  |  |  |  |
| Asia Pacific–high income | 1110.6(1010.1-1223.9) | 639.9(582.0-705.2) |  | 921.9(845.7-1005.2) | 492.9(452.2-537.4) |  | -0.94(-1.06--0.81) |
| Central Asia | 193.5(176.3-210.4) | 277.3(252.7-301.7) |  | 279.7(249.7-309.6) | 307.6(274.6-340.5) |  | 0.50(0.31-0.70) |
| East Asia | 1951.0(1780.5-2122.4) | 155.0(141.5-168.6) |  | 2062.4(1888.3-2241.5) | 138.8(127.1-150.9) |  | -0.65(-0.82--0.49) |
| South Asia | 1365.2(1249.7-1478.2) | 123.1(112.7-133.3) |  | 2224.9(2015.3-2422.4) | 124.8(113.1-135.9) |  | -0.04(-0.08--0.01) |
| Southeast Asia | 272.0(245.7-297.9) | 58.3(52.6-63.8) |  | 387.0(343.9-428.5) | 58.6(52.1-64.9) |  | -0.11(-0.17--0.05) |
| Australasia | 16.3(14.6-17.9) | 80.3(72.1-88.5) |  | 25.2(22.6-27.9) | 88.9(79.5-98.3) |  | 0.41(0.35-0.49) |
| Caribbean | 59.1(53.2-65.2) | 167.4(150.7-184.7) |  | 102.1(90.9-113.4) | 220.6(196.6-245.0) |  | 0.92(0.87-0.96) |
| Central Europe | 437.7(398.4-477.1) | 352.6(321.0-384.4) |  | 362.9(325.1-402.5) | 316.1(283.2-350.6) |  | -0.43(-0.47--0.40) |
| Eastern Europe | 688.0(635.0-740.2) | 303.2(279.8-326.2) |  | 773.5(709.3-834.2) | 368.0(337.4-396.8) |  | 0.86(0.72-1.01) |
| Western Europe | 1142.8(1032.8-1261.6) | 296.3(267.8-327.1) |  | 1484.9(1347.0-1637.9) | 343.0(311.1-378.3) |  | 0.45(0.41-0.48) |
| Andean Latin America | 94.1(85.5-103.0) | 245.4(222.9-268.5) |  | 191.5(171.5-210.0) | 311.6(279.1-341.7) |  | 0.78(0.73-0.84) |
| Central Latin America | 358.6(330.3-386.4) | 218.5(201.2-235.4) |  | 637.8(581.2-699.1) | 249.6(227.5-273.6) |  | 0.38(0.31-0.45) |
| Southern Latin America | 113.5(101.2-126.2) | 229.2(204.2-254.6) |  | 207.2(183.8-231.1) | 315.9(280.1-352.2) |  | 1.21(1.18-1.24) |
| Tropical Latin America | 265.5(245.3-284.9) | 173.0(159.8-185.6) |  | 390.0(359.5-419.3) | 178.3(164.4-191.7) |  | 0.19(0.00-0.38) |
| North Africa and Middle East | 963.3(866.0-1064.7) | 282.6(254.1-312.3) |  | 1620.7(1456.7-1789.3) | 270.0(242.7-298.1) |  | -0.21(-0.23--0.19) |
| North America–high income | 363.9(336.1-391.0) | 129.6(119.7-139.3) |  | 573.1(527.2-619.8) | 158.8(146.1-171.7) |  | 0.69(0.52-0.86) |
| Oceania | 5.9(5.2-6.6) | 91.4(80.9-102.4) |  | 14.0(12.4-15.7) | 110.7(98.0-124.5) |  | 0.74(0.71-0.76) |
| Central Sub-Saharan Africa | 201.1(180.5-222.1) | 365.5(328.1-403.7) |  | 477.9(428.7-526.3) | 392.8(352.3-432.5) |  | 0.31(0.26-0.36) |
| Eastern Sub-Saharan Africa | 774.1(700.2-842.7) | 404.1(365.5-439.9) |  | 1678.4(1513.8-1837.3) | 426.9(385.0-467.3) |  | 0.14(0.09-0.18) |
| Southern Sub-Saharan Africa | 202.2(185.5-218.4) | 385.2(353.5-416.2) |  | 247.7(225.6-269.3) | 320.1(291.6-348.1) |  | -0.51(-0.57--0.45) |
| Western Sub-Saharan Africa | 857.2(775.2-940.8) | 445.9(403.3-489.4) |  | 1953.3(1747.8-2152.6) | 450.3(402.9-496.2) |  | 0.02(-0.02-0.05) |
