# Supplementary material for: The burden of liver cirrhosis and underlying etiologies: results from the global burden of disease study 2017
Source: Aging (Albany NY). 2021 Jan 12;13(1):279–300. doi: 10.18632/aging.104127 (PMC7835066; doi:10.18632/aging.104127)
Supplement: Supplementary Table 6 [file aging-13-104127-s007.docx]

**Supplemental Table 6. The prevalence cases and temporal trend of liver cirrhosis caused by each etiology in 195 countries and territories.**

| Region | Cause | Case in 1990 No.×10^3^ | Case in 2017 No.×10^3^ | Change in absolute number (%) | EAPC (%) |
| --- | --- | --- | --- | --- | --- |
| Afghanistan | Cirrhosis | 2028.30 | 5787.36 | 185.33 | -0.45(-0.56--0.35) |
| Afghanistan | Cirrhosis due to hepatitis B | 849.73 | 2108.78 | 148.17 | -1.02(-1.17--0.87) |
| Afghanistan | Cirrhosis due to hepatitis C | 234.37 | 497.63 | 112.33 | -1.55(-1.62--1.47) |
| Afghanistan | Cirrhosis due to alcohol use | 5.44 | 15.21 | 179.60 | -0.20(-0.70-0.31) |
| Afghanistan | Cirrhosis due to NASH | 904.60 | 3039.76 | 236.03 | 0.23(-0.06-0.52) |
| Afghanistan | Cirrhosis due to other causes | 34.16 | 125.99 | 268.82 | 0.66(0.56-0.76) |
| Albania | Cirrhosis | 395.73 | 513.42 | 29.74 | 1.84(1.75-1.94) |
| Albania | Cirrhosis due to hepatitis B | 88.29 | 64.88 | -26.51 | -0.14(-0.32-0.03) |
| Albania | Cirrhosis due to hepatitis C | 37.62 | 29.51 | -21.56 | -0.33(-0.39--0.26) |
| Albania | Cirrhosis due to alcohol use | 14.21 | 23.97 | 68.68 | 2.77(2.69-2.85) |
| Albania | Cirrhosis due to NASH | 243.45 | 385.70 | 58.43 | 2.59(2.51-2.67) |
| Albania | Cirrhosis due to other causes | 12.17 | 9.36 | -23.09 | -0.30(-0.32--0.28) |
| Algeria | Cirrhosis | 3578.43 | 8743.09 | 144.33 | 1.62(1.53-1.71) |
| Algeria | Cirrhosis due to hepatitis B | 1258.16 | 1546.55 | 22.92 | -1.25(-1.42--1.09) |
| Algeria | Cirrhosis due to hepatitis C | 166.14 | 311.87 | 87.72 | 0.60(0.45-0.74) |
| Algeria | Cirrhosis due to alcohol use | 10.04 | 29.66 | 195.42 | 2.37(2.22-2.52) |
| Algeria | Cirrhosis due to NASH | 2069.68 | 6748.17 | 226.05 | 2.86(2.77-2.95) |
| Algeria | Cirrhosis due to other causes | 74.41 | 106.85 | 43.60 | -0.55(-0.62--0.48) |
| American Samoa | Cirrhosis | 11.40 | 17.20 | 50.88 | 1.07(1.04-1.10) |
| American Samoa | Cirrhosis due to hepatitis B | 4.01 | 4.55 | 13.47 | 0.05(0.02-0.09) |
| American Samoa | Cirrhosis due to hepatitis C | 0.49 | 0.55 | 12.24 | -0.03(-0.09-0.03) |
| American Samoa | Cirrhosis due to alcohol use | 0.03 | 0.05 | 66.67 | 1.71(1.61-1.82) |
| American Samoa | Cirrhosis due to NASH | 6.83 | 11.99 | 75.55 | 1.62(1.60-1.64) |
| American Samoa | Cirrhosis due to other causes | 0.04 | 0.05 | 25.00 | 0.66(0.60-0.72) |
| Andorra | Cirrhosis | 5.29 | 10.45 | 97.54 | 1.05(0.92-1.17) |
| Andorra | Cirrhosis due to hepatitis B | 0.52 | 0.64 | 23.08 | -0.48(-0.57--0.40) |
| Andorra | Cirrhosis due to hepatitis C | 0.32 | 0.47 | 46.88 | -0.04(-0.09-0.01) |
| Andorra | Cirrhosis due to alcohol use | 0.40 | 0.81 | 102.50 | 0.90(0.74-1.07) |
| Andorra | Cirrhosis due to NASH | 3.92 | 8.27 | 110.97 | 1.29(1.13-1.45) |
| Andorra | Cirrhosis due to other causes | 0.13 | 0.25 | 92.31 | 0.88(0.83-0.93) |
| Angola | Cirrhosis | 2231.69 | 5300.41 | 137.51 | -0.70(-0.78--0.62) |
| Angola | Cirrhosis due to hepatitis B | 1326.05 | 2720.30 | 105.14 | -1.35(-1.52--1.18) |
| Angola | Cirrhosis due to hepatitis C | 394.05 | 741.12 | 88.08 | -1.42(-1.50--1.33) |
| Angola | Cirrhosis due to alcohol use | 14.97 | 46.07 | 207.75 | 0.44(0.37-0.51) |
| Angola | Cirrhosis due to NASH | 457.78 | 1683.82 | 267.82 | 1.14(1.04-1.24) |
| Angola | Cirrhosis due to other causes | 38.84 | 109.10 | 180.90 | 0.01(-0.04-0.06) |
| Antigua and Barbuda | Cirrhosis | 6.90 | 15.64 | 126.67 | 1.58(1.46-1.70) |
| Antigua and Barbuda | Cirrhosis due to hepatitis B | 0.66 | 0.85 | 28.79 | -0.59(-0.84--0.33) |
| Antigua and Barbuda | Cirrhosis due to hepatitis C | 0.49 | 0.61 | 24.49 | -0.69(-0.77--0.62) |
| Antigua and Barbuda | Cirrhosis due to alcohol use | 0.15 | 0.38 | 153.33 | 2.02(1.97-2.07) |
| Antigua and Barbuda | Cirrhosis due to NASH | 5.51 | 13.60 | 146.82 | 1.91(1.80-2.02) |
| Antigua and Barbuda | Cirrhosis due to other causes | 0.10 | 0.19 | 90.00 | 0.97(0.92-1.02) |
| Argentina | Cirrhosis | 2656.47 | 4796.56 | 80.56 | 1.14(1.10-1.18) |
| Argentina | Cirrhosis due to hepatitis B | 185.98 | 234.27 | 25.97 | -0.32(-0.42--0.21) |
| Argentina | Cirrhosis due to hepatitis C | 273.07 | 314.19 | 15.06 | -0.43(-0.50--0.35) |
| Argentina | Cirrhosis due to alcohol use | 151.43 | 267.85 | 76.88 | 1.06(0.89-1.22) |
| Argentina | Cirrhosis due to NASH | 1975.82 | 3848.09 | 94.76 | 1.42(1.40-1.44) |
| Argentina | Cirrhosis due to other causes | 70.17 | 132.16 | 88.34 | 1.25(1.24-1.27) |
| Armenia | Cirrhosis | 628.47 | 655.15 | 4.25 | 0.59(0.55-0.64) |
| Armenia | Cirrhosis due to hepatitis B | 229.23 | 166.11 | -27.54 | -1.01(-1.17--0.85) |
| Armenia | Cirrhosis due to hepatitis C | 154.26 | 125.54 | -18.62 | -0.23(-0.30--0.17) |
| Armenia | Cirrhosis due to alcohol use | 10.10 | 16.48 | 63.17 | 2.69(2.56-2.82) |
| Armenia | Cirrhosis due to NASH | 227.76 | 339.76 | 49.17 | 2.07(2.02-2.12) |
| Armenia | Cirrhosis due to other causes | 7.11 | 7.25 | 1.97 | 0.63(0.57-0.68) |
| Australia | Cirrhosis | 1817.40 | 3343.83 | 83.99 | 1.00(0.95-1.06) |
| Australia | Cirrhosis due to hepatitis B | 324.78 | 371.63 | 14.43 | -0.99(-1.25--0.73) |
| Australia | Cirrhosis due to hepatitis C | 220.35 | 286.90 | 30.20 | -0.29(-0.34--0.24) |
| Australia | Cirrhosis due to alcohol use | 24.96 | 44.42 | 77.96 | 0.98(0.88-1.09) |
| Australia | Cirrhosis due to NASH | 1234.70 | 2620.55 | 112.24 | 1.57(1.44-1.70) |
| Australia | Cirrhosis due to other causes | 12.61 | 20.34 | 61.30 | 0.53(0.45-0.61) |
| Austria | Cirrhosis | 847.88 | 1243.76 | 46.69 | 0.93(0.86-1.00) |
| Austria | Cirrhosis due to hepatitis B | 92.14 | 88.50 | -3.95 | -0.70(-0.81--0.60) |
| Austria | Cirrhosis due to hepatitis C | 70.72 | 66.41 | -6.09 | -0.56(-0.61--0.50) |
| Austria | Cirrhosis due to alcohol use | 105.40 | 122.05 | 15.80 | 0.18(0.11-0.25) |
| Austria | Cirrhosis due to NASH | 547.95 | 928.25 | 69.40 | 1.42(1.32-1.53) |
| Austria | Cirrhosis due to other causes | 31.66 | 38.54 | 21.73 | 0.34(0.30-0.38) |
| Azerbaijan | Cirrhosis | 1406.33 | 2294.81 | 63.18 | 0.55(0.52-0.57) |
| Azerbaijan | Cirrhosis due to hepatitis B | 545.50 | 693.78 | 27.18 | -0.54(-0.66--0.41) |
| Azerbaijan | Cirrhosis due to hepatitis C | 344.70 | 438.35 | 27.17 | -0.44(-0.58--0.31) |
| Azerbaijan | Cirrhosis due to alcohol use | 25.06 | 55.59 | 121.83 | 2.03(1.93-2.13) |
| Azerbaijan | Cirrhosis due to NASH | 473.12 | 1080.41 | 128.36 | 2.02(1.86-2.18) |
| Azerbaijan | Cirrhosis due to other causes | 17.94 | 26.69 | 48.77 | 0.31(0.24-0.38) |
| Bahrain | Cirrhosis | 100.47 | 474.61 | 372.39 | 2.09(1.94-2.23) |
| Bahrain | Cirrhosis due to hepatitis B | 24.27 | 63.32 | 160.90 | 0.09(-0.15-0.33) |
| Bahrain | Cirrhosis due to hepatitis C | 3.39 | 12.68 | 274.04 | 0.80(0.62-0.98) |
| Bahrain | Cirrhosis due to alcohol use | 0.30 | 1.46 | 386.67 | 1.68(1.39-1.97) |
| Bahrain | Cirrhosis due to NASH | 71.30 | 394.07 | 452.69 | 2.63(2.51-2.74) |
| Bahrain | Cirrhosis due to other causes | 1.21 | 3.07 | 153.72 | -0.84(-0.98--0.70) |
| Bangladesh | Cirrhosis | 11268.10 | 20445.14 | 81.44 | 0.88(0.80-0.97) |
| Bangladesh | Cirrhosis due to hepatitis B | 5470.86 | 6587.16 | 20.40 | -0.85(-0.95--0.75) |
| Bangladesh | Cirrhosis due to hepatitis C | 743.09 | 785.25 | 5.67 | -0.97(-1.10--0.84) |
| Bangladesh | Cirrhosis due to alcohol use | 108.58 | 214.56 | 97.61 | 1.45(1.34-1.55) |
| Bangladesh | Cirrhosis due to NASH | 4755.55 | 12648.66 | 165.98 | 2.51(2.38-2.65) |
| Bangladesh | Cirrhosis due to other causes | 190.02 | 209.51 | 10.26 | -0.92(-0.97--0.86) |
| Barbados | Cirrhosis | 36.71 | 60.70 | 65.35 | 1.29(1.24-1.33) |
| Barbados | Cirrhosis due to hepatitis B | 2.63 | 2.72 | 3.42 | -0.70(-0.84--0.56) |
| Barbados | Cirrhosis due to hepatitis C | 2.00 | 1.98 | -1.00 | -0.66(-0.71--0.62) |
| Barbados | Cirrhosis due to alcohol use | 0.67 | 1.34 | 100.00 | 1.77(1.68-1.86) |
| Barbados | Cirrhosis due to NASH | 31.01 | 54.03 | 74.23 | 1.50(1.44-1.56) |
| Barbados | Cirrhosis due to other causes | 0.39 | 0.63 | 61.54 | 1.05(0.97-1.13) |
| Belarus | Cirrhosis | 1433.00 | 1596.88 | 11.44 | 0.78(0.71-0.85) |
| Belarus | Cirrhosis due to hepatitis B | 403.41 | 292.62 | -27.46 | -1.08(-1.29--0.88) |
| Belarus | Cirrhosis due to hepatitis C | 161.41 | 171.40 | 6.19 | 0.96(0.74-1.19) |
| Belarus | Cirrhosis due to alcohol use | 53.24 | 89.88 | 68.82 | 2.98(2.75-3.22) |
| Belarus | Cirrhosis due to NASH | 786.11 | 1012.10 | 28.75 | 1.29(1.23-1.35) |
| Belarus | Cirrhosis due to other causes | 28.82 | 30.88 | 7.15 | 0.87(0.75-0.99) |
| Belgium | Cirrhosis | 1007.37 | 1455.24 | 44.46 | 0.87(0.76-0.98) |
| Belgium | Cirrhosis due to hepatitis B | 110.90 | 103.21 | -6.93 | -0.89(-0.98--0.81) |
| Belgium | Cirrhosis due to hepatitis C | 96.90 | 97.37 | 0.49 | -0.55(-0.65--0.46) |
| Belgium | Cirrhosis due to alcohol use | 76.03 | 103.68 | 36.37 | 0.80(0.72-0.89) |
| Belgium | Cirrhosis due to NASH | 700.60 | 1120.24 | 59.90 | 1.25(1.09-1.41) |
| Belgium | Cirrhosis due to other causes | 22.94 | 30.75 | 34.05 | 0.67(0.64-0.71) |
| Belize | Cirrhosis | 18.90 | 60.47 | 219.95 | 1.57(1.54-1.61) |
| Belize | Cirrhosis due to hepatitis B | 2.49 | 4.37 | 75.50 | -0.79(-1.01--0.57) |
| Belize | Cirrhosis due to hepatitis C | 1.74 | 3.12 | 79.31 | -0.64(-0.73--0.56) |
| Belize | Cirrhosis due to alcohol use | 0.36 | 1.49 | 313.89 | 2.36(2.30-2.41) |
| Belize | Cirrhosis due to NASH | 14.00 | 50.63 | 261.64 | 2.04(1.95-2.12) |
| Belize | Cirrhosis due to other causes | 0.31 | 0.86 | 177.42 | 0.96(0.92-1.00) |
| Benin | Cirrhosis | 1075.83 | 2434.59 | 126.30 | -0.42(-0.50--0.33) |
| Benin | Cirrhosis due to hepatitis B | 694.40 | 1353.68 | 94.94 | -1.13(-1.28--0.98) |
| Benin | Cirrhosis due to hepatitis C | 164.84 | 300.29 | 82.17 | -1.02(-1.07--0.97) |
| Benin | Cirrhosis due to alcohol use | 7.43 | 20.27 | 172.81 | 0.34(0.25-0.44) |
| Benin | Cirrhosis due to NASH | 186.76 | 706.63 | 278.36 | 1.82(1.77-1.87) |
| Benin | Cirrhosis due to other causes | 22.39 | 53.72 | 139.93 | -0.11(-0.17--0.06) |
| Bermuda | Cirrhosis | 9.45 | 15.76 | 66.77 | 1.59(1.55-1.63) |
| Bermuda | Cirrhosis due to hepatitis B | 0.53 | 0.63 | 18.87 | 0.22(0.20-0.24) |
| Bermuda | Cirrhosis due to hepatitis C | 0.43 | 0.39 | -9.30 | -0.82(-0.88--0.75) |
| Bermuda | Cirrhosis due to alcohol use | 0.23 | 0.35 | 52.17 | 0.86(0.76-0.96) |
| Bermuda | Cirrhosis due to NASH | 8.16 | 14.25 | 74.63 | 1.78(1.73-1.83) |
| Bermuda | Cirrhosis due to other causes | 0.09 | 0.14 | 55.56 | 1.12(1.02-1.22) |
| Bhutan | Cirrhosis | 74.84 | 153.02 | 104.46 | 0.65(0.52-0.77) |
| Bhutan | Cirrhosis due to hepatitis B | 35.53 | 45.23 | 27.30 | -1.19(-1.45--0.94) |
| Bhutan | Cirrhosis due to hepatitis C | 5.09 | 6.30 | 23.77 | -1.24(-1.27--1.21) |
| Bhutan | Cirrhosis due to alcohol use | 0.68 | 1.52 | 123.53 | 0.74(0.60-0.88) |
| Bhutan | Cirrhosis due to NASH | 32.71 | 98.73 | 201.83 | 2.17(2.13-2.21) |
| Bhutan | Cirrhosis due to other causes | 0.83 | 1.24 | 49.40 | -0.55(-0.60--0.50) |
| Bolivia | Cirrhosis | 657.34 | 1693.47 | 157.62 | 1.49(1.44-1.54) |
| Bolivia | Cirrhosis due to hepatitis B | 164.00 | 252.26 | 53.82 | -0.62(-0.80--0.43) |
| Bolivia | Cirrhosis due to hepatitis C | 82.15 | 127.17 | 54.80 | -0.52(-0.59--0.46) |
| Bolivia | Cirrhosis due to alcohol use | 14.53 | 49.30 | 239.30 | 2.42(2.36-2.48) |
| Bolivia | Cirrhosis due to NASH | 381.95 | 1228.01 | 221.51 | 2.40(2.32-2.49) |
| Bolivia | Cirrhosis due to other causes | 14.71 | 36.73 | 149.69 | 1.12(1.04-1.20) |
| Bosnia and Herzegovina | Cirrhosis | 677.25 | 763.84 | 12.79 | 1.51(1.45-1.56) |
| Bosnia and Herzegovina | Cirrhosis due to hepatitis B | 127.38 | 81.41 | -36.09 | -0.84(-1.10--0.58) |
| Bosnia and Herzegovina | Cirrhosis due to hepatitis C | 62.12 | 39.10 | -37.06 | -0.77(-0.89--0.64) |
| Bosnia and Herzegovina | Cirrhosis due to alcohol use | 30.33 | 31.51 | 3.89 | 1.09(0.92-1.26) |
| Bosnia and Herzegovina | Cirrhosis due to NASH | 441.78 | 601.22 | 36.09 | 2.27(2.12-2.41) |
| Bosnia and Herzegovina | Cirrhosis due to other causes | 15.63 | 10.60 | -32.18 | -0.36(-0.46--0.26) |
| Botswana | Cirrhosis | 206.99 | 411.02 | 98.57 | 0.62(0.49-0.75) |
| Botswana | Cirrhosis due to hepatitis B | 114.31 | 148.93 | 30.29 | -0.82(-1.00--0.64) |
| Botswana | Cirrhosis due to hepatitis C | 19.53 | 28.22 | 44.50 | -0.63(-0.67--0.59) |
| Botswana | Cirrhosis due to alcohol use | 1.53 | 4.22 | 175.82 | 1.61(1.51-1.71) |
| Botswana | Cirrhosis due to NASH | 67.59 | 222.48 | 229.16 | 2.34(2.28-2.40) |
| Botswana | Cirrhosis due to other causes | 4.04 | 7.17 | 77.48 | 0.00(-0.05-0.04) |
| Brazil | Cirrhosis | 18584.90 | 38425.70 | 106.76 | 1.39(1.34-1.44) |
| Brazil | Cirrhosis due to hepatitis B | 5828.36 | 7482.71 | 28.38 | -0.44(-0.49--0.40) |
| Brazil | Cirrhosis due to hepatitis C | 2444.15 | 3374.52 | 38.07 | 0.12(-0.10-0.34) |
| Brazil | Cirrhosis due to alcohol use | 267.35 | 518.35 | 93.88 | 1.05(0.95-1.15) |
| Brazil | Cirrhosis due to NASH | 9784.61 | 26670.92 | 172.58 | 2.40(2.39-2.42) |
| Brazil | Cirrhosis due to other causes | 260.43 | 379.21 | 45.61 | 0.18(-0.02-0.37) |
| Brunei | Cirrhosis | 27.36 | 63.88 | 133.48 | 1.32(1.27-1.37) |
| Brunei | Cirrhosis due to hepatitis B | 9.85 | 18.01 | 82.84 | 0.42(0.31-0.53) |
| Brunei | Cirrhosis due to hepatitis C | 2.16 | 3.66 | 69.44 | -0.05(-0.14-0.06) |
| Brunei | Cirrhosis due to alcohol use | 0.76 | 2.14 | 181.58 | 1.70(1.54-1.86) |
| Brunei | Cirrhosis due to NASH | 12.94 | 37.36 | 188.72 | 2.15(2.11-2.19) |
| Brunei | Cirrhosis due to other causes | 1.66 | 2.70 | 62.65 | -0.17(-0.20--0.13) |
| Bulgaria | Cirrhosis | 1501.27 | 1284.71 | -14.43 | 0.37(0.25-0.50) |
| Bulgaria | Cirrhosis due to hepatitis B | 444.55 | 271.02 | -39.03 | -0.61(-0.82--0.40) |
| Bulgaria | Cirrhosis due to hepatitis C | 98.27 | 70.33 | -28.43 | -0.31(-0.36--0.26) |
| Bulgaria | Cirrhosis due to alcohol use | 81.73 | 86.69 | 6.07 | 1.06(0.97-1.15) |
| Bulgaria | Cirrhosis due to NASH | 850.56 | 836.77 | -1.62 | 0.78(0.67-0.90) |
| Bulgaria | Cirrhosis due to other causes | 26.17 | 19.90 | -23.96 | -0.31(-0.40--0.22) |
| Burkina Faso | Cirrhosis | 2729.14 | 4977.61 | 82.39 | -0.80(-0.90--0.71) |
| Burkina Faso | Cirrhosis due to hepatitis B | 1529.91 | 2642.93 | 72.75 | -1.18(-1.44--0.93) |
| Burkina Faso | Cirrhosis due to hepatitis C | 795.62 | 1159.11 | 45.69 | -1.28(-1.45--1.11) |
| Burkina Faso | Cirrhosis due to alcohol use | 18.04 | 46.75 | 159.15 | 0.46(0.31-0.62) |
| Burkina Faso | Cirrhosis due to NASH | 333.97 | 1017.12 | 204.55 | 1.21(1.16-1.27) |
| Burkina Faso | Cirrhosis due to other causes | 51.59 | 111.70 | 116.51 | -0.11(-0.23-0.00) |
| Burundi | Cirrhosis | 666.21 | 1205.39 | 80.93 | -0.53(-0.63--0.42) |
| Burundi | Cirrhosis due to hepatitis B | 352.91 | 553.20 | 56.75 | -1.14(-1.31--0.96) |
| Burundi | Cirrhosis due to hepatitis C | 80.41 | 139.77 | 73.82 | -0.67(-0.76--0.58) |
| Burundi | Cirrhosis due to alcohol use | 12.99 | 25.31 | 94.84 | -0.41(-0.53--0.29) |
| Burundi | Cirrhosis due to NASH | 196.76 | 440.72 | 123.99 | 0.39(0.33-0.46) |
| Burundi | Cirrhosis due to other causes | 23.15 | 46.39 | 100.39 | -0.01(-0.03-0.02) |
| Cambodia | Cirrhosis | 2243.64 | 3907.06 | 74.14 | 0.39(0.33-0.46) |
| Cambodia | Cirrhosis due to hepatitis B | 1029.02 | 1468.67 | 42.73 | -0.53(-0.67--0.39) |
| Cambodia | Cirrhosis due to hepatitis C | 573.90 | 723.58 | 26.08 | -0.77(-0.81--0.73) |
| Cambodia | Cirrhosis due to alcohol use | 4.50 | 15.94 | 254.22 | 3.39(3.21-3.57) |
| Cambodia | Cirrhosis due to NASH | 629.45 | 1687.86 | 168.15 | 2.24(2.15-2.32) |
| Cambodia | Cirrhosis due to other causes | 6.77 | 11.00 | 62.48 | 0.17(0.03-0.32) |
| Cameroon | Cirrhosis | 2823.17 | 6752.90 | 139.20 | -0.60(-0.69--0.50) |
| Cameroon | Cirrhosis due to hepatitis B | 1771.17 | 3678.08 | 107.66 | -1.17(-1.31--1.03) |
| Cameroon | Cirrhosis due to hepatitis C | 371.98 | 805.57 | 116.56 | -0.85(-0.95--0.75) |
| Cameroon | Cirrhosis due to alcohol use | 18.29 | 52.68 | 188.03 | 0.32(0.22-0.41) |
| Cameroon | Cirrhosis due to NASH | 616.66 | 2094.45 | 239.64 | 0.78(0.72-0.84) |
| Cameroon | Cirrhosis due to other causes | 45.06 | 122.11 | 170.99 | 0.03(-0.01-0.07) |
| Canada | Cirrhosis | 2353.89 | 4001.26 | 69.99 | 0.94(0.87-1.01) |
| Canada | Cirrhosis due to hepatitis B | 430.45 | 462.49 | 7.44 | -0.77(-0.84--0.70) |
| Canada | Cirrhosis due to hepatitis C | 95.96 | 130.77 | 36.28 | 0.22(0.14-0.29) |
| Canada | Cirrhosis due to alcohol use | 36.39 | 66.15 | 81.78 | 1.26(1.22-1.30) |
| Canada | Cirrhosis due to NASH | 1761.87 | 3291.54 | 86.82 | 1.28(1.19-1.37) |
| Canada | Cirrhosis due to other causes | 29.22 | 50.31 | 72.18 | 1.05(1.00-1.10) |
| Cape Verde | Cirrhosis | 74.82 | 122.26 | 63.41 | 0.01(-0.13-0.15) |
| Cape Verde | Cirrhosis due to hepatitis B | 43.18 | 52.28 | 21.07 | -1.30(-1.50--1.11) |
| Cape Verde | Cirrhosis due to hepatitis C | 11.18 | 11.72 | 4.83 | -1.43(-1.51--1.34) |
| Cape Verde | Cirrhosis due to alcohol use | 0.46 | 1.36 | 195.65 | 2.36(2.19-2.53) |
| Cape Verde | Cirrhosis due to NASH | 18.55 | 54.55 | 194.07 | 2.53(2.36-2.69) |
| Cape Verde | Cirrhosis due to other causes | 1.45 | 2.35 | 62.07 | 0.13(0.07-0.19) |
| Central African Republic | Cirrhosis | 531.67 | 809.17 | 52.19 | -0.59(-0.69--0.50) |
| Central African Republic | Cirrhosis due to hepatitis B | 337.79 | 460.20 | 36.24 | -1.11(-1.27--0.94) |
| Central African Republic | Cirrhosis due to hepatitis C | 69.37 | 91.84 | 32.39 | -0.98(-1.02--0.95) |
| Central African Republic | Cirrhosis due to alcohol use | 3.83 | 7.66 | 100.00 | 0.59(0.38-0.80) |
| Central African Republic | Cirrhosis due to NASH | 111.48 | 232.11 | 108.21 | 0.81(0.70-0.92) |
| Central African Republic | Cirrhosis due to other causes | 9.20 | 17.36 | 88.70 | 0.33(0.23-0.43) |
| Chad | Cirrhosis | 1508.99 | 3209.97 | 112.72 | -0.69(-0.78--0.61) |
| Chad | Cirrhosis due to hepatitis B | 967.55 | 1992.61 | 105.94 | -0.82(-0.96--0.69) |
| Chad | Cirrhosis due to hepatitis C | 263.57 | 471.25 | 78.80 | -1.28(-1.35--1.21) |
| Chad | Cirrhosis due to alcohol use | 9.15 | 22.62 | 147.21 | -0.17(-0.27--0.08) |
| Chad | Cirrhosis due to NASH | 239.59 | 650.89 | 171.67 | 0.24(0.22-0.26) |
| Chad | Cirrhosis due to other causes | 29.13 | 72.60 | 149.23 | -0.13(-0.27-0.02) |
| Chile | Cirrhosis | 1203.71 | 2580.33 | 114.36 | 1.79(1.70-1.88) |
| Chile | Cirrhosis due to hepatitis B | 107.97 | 151.58 | 40.39 | 0.04(-0.05-0.14) |
| Chile | Cirrhosis due to hepatitis C | 87.76 | 126.78 | 44.46 | 0.59(0.48-0.69) |
| Chile | Cirrhosis due to alcohol use | 79.35 | 140.14 | 76.61 | 1.27(1.17-1.38) |
| Chile | Cirrhosis due to NASH | 891.84 | 2097.08 | 135.14 | 2.11(1.99-2.23) |
| Chile | Cirrhosis due to other causes | 36.79 | 64.75 | 76.00 | 1.10(1.02-1.19) |
| China | Cirrhosis | 268707.61 | 416185.99 | 54.88 | 0.95(0.87-1.02) |
| China | Cirrhosis due to hepatitis B | 127486.97 | 140140.55 | 9.93 | -0.27(-0.40--0.13) |
| China | Cirrhosis due to hepatitis C | 28664.95 | 32865.19 | 14.65 | -0.27(-0.34--0.20) |
| China | Cirrhosis due to alcohol use | 2917.92 | 6413.90 | 119.81 | 2.28(2.11-2.44) |
| China | Cirrhosis due to NASH | 107770.23 | 234822.31 | 117.89 | 2.23(2.18-2.27) |
| China | Cirrhosis due to other causes | 1867.55 | 1944.04 | 4.10 | -0.73(-0.90--0.55) |
| Colombia | Cirrhosis | 4245.19 | 8588.40 | 102.31 | 1.06(1.00-1.13) |
| Colombia | Cirrhosis due to hepatitis B | 1626.35 | 1778.74 | 9.37 | -1.12(-1.26--0.98) |
| Colombia | Cirrhosis due to hepatitis C | 532.09 | 696.05 | 30.81 | -0.37(-0.47--0.26) |
| Colombia | Cirrhosis due to alcohol use | 114.99 | 358.20 | 211.51 | 2.32(2.20-2.43) |
| Colombia | Cirrhosis due to NASH | 1924.85 | 5664.21 | 194.27 | 2.35(2.32-2.39) |
| Colombia | Cirrhosis due to other causes | 46.92 | 91.20 | 94.37 | 0.74(0.67-0.82) |
| Comoros | Cirrhosis | 60.18 | 103.65 | 72.23 | 0.29(0.21-0.36) |
| Comoros | Cirrhosis due to hepatitis B | 29.04 | 37.76 | 30.03 | -0.80(-0.94--0.66) |
| Comoros | Cirrhosis due to hepatitis C | 5.40 | 8.35 | 54.63 | -0.04(-0.07--0.01) |
| Comoros | Cirrhosis due to alcohol use | 0.64 | 1.56 | 143.75 | 1.64(1.49-1.80) |
| Comoros | Cirrhosis due to NASH | 23.34 | 52.86 | 126.48 | 1.36(1.33-1.39) |
| Comoros | Cirrhosis due to other causes | 1.76 | 3.12 | 77.27 | 0.45(0.35-0.55) |
| Congo | Cirrhosis | 482.14 | 867.80 | 79.99 | -0.44(-0.51--0.38) |
| Congo | Cirrhosis due to hepatitis B | 279.44 | 404.43 | 44.73 | -1.23(-1.40--1.07) |
| Congo | Cirrhosis due to hepatitis C | 79.01 | 120.46 | 52.46 | -1.07(-1.10--1.04) |
| Congo | Cirrhosis due to alcohol use | 3.54 | 9.21 | 160.17 | 1.02(0.88-1.16) |
| Congo | Cirrhosis due to NASH | 111.92 | 316.90 | 183.15 | 1.31(1.27-1.35) |
| Congo | Cirrhosis due to other causes | 8.22 | 16.80 | 104.38 | -0.02(-0.06-0.03) |
| Costa Rica | Cirrhosis | 338.47 | 751.64 | 122.07 | 1.36(1.29-1.43) |
| Costa Rica | Cirrhosis due to hepatitis B | 77.45 | 81.94 | 5.80 | -1.51(-1.82--1.21) |
| Costa Rica | Cirrhosis due to hepatitis C | 40.18 | 62.18 | 54.75 | -0.02(-0.07-0.02) |
| Costa Rica | Cirrhosis due to alcohol use | 14.39 | 45.37 | 215.29 | 2.42(2.32-2.51) |
| Costa Rica | Cirrhosis due to NASH | 201.66 | 552.47 | 173.96 | 2.19(2.17-2.22) |
| Costa Rica | Cirrhosis due to other causes | 4.79 | 9.68 | 102.09 | 0.88(0.80-0.95) |
| Cote d'Ivoire | Cirrhosis | 2630.37 | 5106.27 | 94.13 | -0.27(-0.32--0.21) |
| Cote d'Ivoire | Cirrhosis due to hepatitis B | 1669.76 | 2752.28 | 64.83 | -0.99(-1.11--0.87) |
| Cote d'Ivoire | Cirrhosis due to hepatitis C | 372.07 | 615.15 | 65.33 | -0.67(-0.72--0.62) |
| Cote d'Ivoire | Cirrhosis due to alcohol use | 19.43 | 49.66 | 155.58 | 0.87(0.83-0.91) |
| Cote d'Ivoire | Cirrhosis due to NASH | 517.22 | 1578.75 | 205.24 | 1.62(1.47-1.77) |
| Cote d'Ivoire | Cirrhosis due to other causes | 51.89 | 110.42 | 112.80 | 0.14(0.10-0.18) |
| Croatia | Cirrhosis | 698.36 | 754.78 | 8.08 | 0.83(0.79-0.88) |
| Croatia | Cirrhosis due to hepatitis B | 128.24 | 99.38 | -22.50 | -0.65(-0.87--0.43) |
| Croatia | Cirrhosis due to hepatitis C | 53.04 | 38.15 | -28.07 | -0.81(-0.89--0.74) |
| Croatia | Cirrhosis due to alcohol use | 62.22 | 50.73 | -18.47 | -0.09(-0.17--0.01) |
| Croatia | Cirrhosis due to NASH | 437.73 | 554.37 | 26.65 | 1.48(1.38-1.59) |
| Croatia | Cirrhosis due to other causes | 17.13 | 12.15 | -29.07 | -0.72(-0.78--0.66) |
| Cuba | Cirrhosis | 1278.15 | 2103.91 | 64.61 | 1.83(1.77-1.89) |
| Cuba | Cirrhosis due to hepatitis B | 95.04 | 103.67 | 9.08 | 0.12(0.11-0.13) |
| Cuba | Cirrhosis due to hepatitis C | 86.34 | 86.46 | 0.14 | -0.17(-0.20--0.15) |
| Cuba | Cirrhosis due to alcohol use | 25.15 | 57.93 | 130.34 | 2.83(2.75-2.90) |
| Cuba | Cirrhosis due to NASH | 1055.29 | 1830.01 | 73.41 | 2.06(1.99-2.14) |
| Cuba | Cirrhosis due to other causes | 16.33 | 25.84 | 58.24 | 1.40(1.33-1.46) |
| Cyprus | Cirrhosis | 70.72 | 165.35 | 133.81 | 1.36(1.27-1.45) |
| Cyprus | Cirrhosis due to hepatitis B | 8.22 | 11.44 | 39.17 | -0.46(-0.58--0.33) |
| Cyprus | Cirrhosis due to hepatitis C | 4.97 | 6.83 | 37.42 | -0.55(-0.59--0.52) |
| Cyprus | Cirrhosis due to alcohol use | 3.30 | 6.93 | 110.00 | 0.85(0.71-0.99) |
| Cyprus | Cirrhosis due to NASH | 52.91 | 137.60 | 160.06 | 1.73(1.60-1.86) |
| Cyprus | Cirrhosis due to other causes | 1.33 | 2.55 | 91.73 | 0.63(0.51-0.75) |
| Czech Republic | Cirrhosis | 1343.55 | 1661.89 | 23.69 | 0.69(0.66-0.72) |
| Czech Republic | Cirrhosis due to hepatitis B | 267.21 | 234.80 | -12.13 | -0.87(-1.04--0.70) |
| Czech Republic | Cirrhosis due to hepatitis C | 118.31 | 98.61 | -16.65 | -0.59(-0.66--0.53) |
| Czech Republic | Cirrhosis due to alcohol use | 88.04 | 118.83 | 34.97 | 1.09(1.04-1.14) |
| Czech Republic | Cirrhosis due to NASH | 841.46 | 1181.68 | 40.43 | 1.22(1.17-1.26) |
| Czech Republic | Cirrhosis due to other causes | 28.53 | 27.97 | -1.96 | -0.29(-0.35--0.22) |
| Democratic Republic of the Congo | Cirrhosis | 7485.45 | 13878.09 | 85.40 | -0.56(-0.69--0.43) |
| Democratic Republic of the Congo | Cirrhosis due to hepatitis B | 4434.20 | 7319.61 | 65.07 | -1.08(-1.32--0.85) |
| Democratic Republic of the Congo | Cirrhosis due to hepatitis C | 1286.24 | 2318.73 | 80.27 | -0.37(-0.55--0.20) |
| Democratic Republic of the Congo | Cirrhosis due to alcohol use | 51.98 | 133.30 | 156.44 | 0.75(0.67-0.83) |
| Democratic Republic of the Congo | Cirrhosis due to NASH | 1573.00 | 3782.32 | 140.45 | 0.46(0.36-0.55) |
| Democratic Republic of the Congo | Cirrhosis due to other causes | 140.02 | 324.14 | 131.50 | 0.42(0.35-0.48) |
| Denmark | Cirrhosis | 435.35 | 659.83 | 51.56 | 1.17(1.05-1.29) |
| Denmark | Cirrhosis due to hepatitis B | 45.55 | 48.65 | 6.81 | -0.07(-0.11--0.03) |
| Denmark | Cirrhosis due to hepatitis C | 23.76 | 26.61 | 11.99 | 0.01(-0.09-0.12) |
| Denmark | Cirrhosis due to alcohol use | 33.48 | 58.42 | 74.49 | 1.65(1.39-1.90) |
| Denmark | Cirrhosis due to NASH | 321.14 | 506.28 | 57.65 | 1.31(1.19-1.44) |
| Denmark | Cirrhosis due to other causes | 11.43 | 19.88 | 73.93 | 1.68(1.56-1.81) |
| Djibouti | Cirrhosis | 59.09 | 162.79 | 175.50 | 0.73(0.70-0.75) |
| Djibouti | Cirrhosis due to hepatitis B | 31.55 | 62.66 | 98.61 | -0.61(-0.74--0.47) |
| Djibouti | Cirrhosis due to hepatitis C | 5.28 | 11.95 | 126.33 | -0.10(-0.17--0.03) |
| Djibouti | Cirrhosis due to alcohol use | 0.73 | 2.72 | 272.60 | 1.78(1.68-1.88) |
| Djibouti | Cirrhosis due to NASH | 19.69 | 81.02 | 311.48 | 2.41(2.34-2.48) |
| Djibouti | Cirrhosis due to other causes | 1.83 | 4.44 | 142.62 | 0.29(0.24-0.34) |
| Dominica | Cirrhosis | 8.00 | 11.47 | 43.38 | 1.57(1.52-1.61) |
| Dominica | Cirrhosis due to hepatitis B | 0.83 | 0.65 | -21.69 | -0.88(-1.04--0.71) |
| Dominica | Cirrhosis due to hepatitis C | 0.64 | 0.50 | -21.88 | -0.79(-0.88--0.69) |
| Dominica | Cirrhosis due to alcohol use | 0.18 | 0.30 | 66.67 | 1.97(1.86-2.08) |
| Dominica | Cirrhosis due to NASH | 6.24 | 9.87 | 58.17 | 1.96(1.89-2.03) |
| Dominica | Cirrhosis due to other causes | 0.12 | 0.15 | 25.00 | 0.92(0.85-0.99) |
| Dominican Republic | Cirrhosis | 631.40 | 1401.07 | 121.90 | 1.57(1.51-1.62) |
| Dominican Republic | Cirrhosis due to hepatitis B | 102.59 | 140.64 | 37.09 | -0.21(-0.29--0.13) |
| Dominican Republic | Cirrhosis due to hepatitis C | 72.30 | 87.00 | 20.33 | -0.87(-0.95--0.79) |
| Dominican Republic | Cirrhosis due to alcohol use | 20.01 | 46.72 | 133.48 | 1.65(1.54-1.76) |
| Dominican Republic | Cirrhosis due to NASH | 422.33 | 1103.58 | 161.31 | 2.19(2.15-2.24) |
| Dominican Republic | Cirrhosis due to other causes | 14.17 | 23.14 | 63.30 | 0.41(0.37-0.45) |
| Ecuador | Cirrhosis | 1479.01 | 3704.67 | 150.48 | 1.39(1.33-1.44) |
| Ecuador | Cirrhosis due to hepatitis B | 231.48 | 374.54 | 61.80 | -0.39(-0.52--0.27) |
| Ecuador | Cirrhosis due to hepatitis C | 105.21 | 171.17 | 62.69 | -0.06(-0.09--0.03) |
| Ecuador | Cirrhosis due to alcohol use | 20.25 | 69.85 | 244.94 | 2.77(2.65-2.90) |
| Ecuador | Cirrhosis due to NASH | 1097.45 | 3035.44 | 176.59 | 1.77(1.70-1.84) |
| Ecuador | Cirrhosis due to other causes | 24.62 | 53.67 | 117.99 | 0.85(0.75-0.94) |
| Egypt | Cirrhosis | 18473.06 | 35389.95 | 91.58 | 0.29(0.26-0.32) |
| Egypt | Cirrhosis due to hepatitis B | 6054.91 | 9440.55 | 55.92 | -0.25(-0.33--0.17) |
| Egypt | Cirrhosis due to hepatitis C | 5299.49 | 7358.34 | 38.85 | -1.22(-1.35--1.09) |
| Egypt | Cirrhosis due to alcohol use | 40.04 | 68.39 | 70.80 | 0.14(-0.04-0.32) |
| Egypt | Cirrhosis due to NASH | 6877.79 | 18219.42 | 164.90 | 1.52(1.49-1.55) |
| Egypt | Cirrhosis due to other causes | 203.64 | 308.94 | 51.71 | -0.50(-0.57--0.43) |
| El Salvador | Cirrhosis | 666.40 | 1138.89 | 70.90 | 1.51(1.46-1.55) |
| El Salvador | Cirrhosis due to hepatitis B | 152.68 | 123.90 | -18.85 | -1.56(-1.87--1.25) |
| El Salvador | Cirrhosis due to hepatitis C | 91.97 | 99.85 | 8.57 | -0.24(-0.29--0.19) |
| El Salvador | Cirrhosis due to alcohol use | 23.75 | 53.89 | 126.91 | 2.56(2.53-2.59) |
| El Salvador | Cirrhosis due to NASH | 388.00 | 847.18 | 118.35 | 2.48(2.40-2.57) |
| El Salvador | Cirrhosis due to other causes | 10.01 | 14.07 | 40.56 | 0.73(0.67-0.79) |
| Equatorial Guinea | Cirrhosis | 88.12 | 247.13 | 180.45 | -0.53(-0.61--0.46) |
| Equatorial Guinea | Cirrhosis due to hepatitis B | 48.65 | 114.17 | 134.68 | -1.25(-1.33--1.17) |
| Equatorial Guinea | Cirrhosis due to hepatitis C | 16.67 | 26.33 | 57.95 | -2.87(-3.05--2.68) |
| Equatorial Guinea | Cirrhosis due to alcohol use | 0.57 | 1.97 | 245.61 | 0.33(0.08-0.57) |
| Equatorial Guinea | Cirrhosis due to NASH | 20.80 | 99.98 | 380.67 | 1.79(1.67-1.91) |
| Equatorial Guinea | Cirrhosis due to other causes | 1.43 | 4.68 | 227.27 | 0.02(-0.05-0.10) |
| Eritrea | Cirrhosis | 379.15 | 723.09 | 90.71 | -0.44(-0.58--0.30) |
| Eritrea | Cirrhosis due to hepatitis B | 238.94 | 384.53 | 60.93 | -1.16(-1.37--0.95) |
| Eritrea | Cirrhosis due to hepatitis C | 39.66 | 73.40 | 85.07 | -0.46(-0.53--0.40) |
| Eritrea | Cirrhosis due to alcohol use | 4.71 | 12.12 | 157.32 | 0.72(0.67-0.77) |
| Eritrea | Cirrhosis due to NASH | 84.26 | 226.93 | 169.32 | 1.03(0.94-1.12) |
| Eritrea | Cirrhosis due to other causes | 11.58 | 26.12 | 125.56 | 0.33(0.31-0.35) |
| Estonia | Cirrhosis | 201.49 | 215.02 | 6.71 | 1.01(0.97-1.05) |
| Estonia | Cirrhosis due to hepatitis B | 53.34 | 36.21 | -32.11 | -0.94(-1.09--0.80) |
| Estonia | Cirrhosis due to hepatitis C | 22.76 | 20.33 | -10.68 | 0.16(-0.09-0.42) |
| Estonia | Cirrhosis due to alcohol use | 7.69 | 12.33 | 60.34 | 2.81(2.59-3.03) |
| Estonia | Cirrhosis due to NASH | 113.47 | 142.10 | 25.23 | 1.73(1.64-1.81) |
| Estonia | Cirrhosis due to other causes | 4.23 | 4.05 | -4.26 | 0.53(0.45-0.61) |
| Ethiopia | Cirrhosis | 8256.89 | 16643.10 | 101.57 | 0.05(-0.01-0.12) |
| Ethiopia | Cirrhosis due to hepatitis B | 5297.99 | 9922.38 | 87.29 | -0.20(-0.33--0.08) |
| Ethiopia | Cirrhosis due to hepatitis C | 788.03 | 1400.12 | 77.67 | -0.40(-0.50--0.29) |
| Ethiopia | Cirrhosis due to alcohol use | 74.35 | 150.15 | 101.95 | -0.02(-0.17-0.12) |
| Ethiopia | Cirrhosis due to NASH | 1902.94 | 4757.30 | 150.00 | 0.84(0.74-0.94) |
| Ethiopia | Cirrhosis due to other causes | 193.58 | 413.15 | 113.43 | 0.22(0.15-0.28) |
| Federated States of Micronesia | Cirrhosis | 21.02 | 28.13 | 33.82 | 1.20(1.16-1.25) |
| Federated States of Micronesia | Cirrhosis due to hepatitis B | 9.64 | 9.41 | -2.39 | -0.02(-0.12-0.07) |
| Federated States of Micronesia | Cirrhosis due to hepatitis C | 1.50 | 1.33 | -11.33 | -0.48(-0.55--0.41) |
| Federated States of Micronesia | Cirrhosis due to alcohol use | 0.07 | 0.11 | 57.14 | 1.98(1.87-2.09) |
| Federated States of Micronesia | Cirrhosis due to NASH | 9.70 | 17.16 | 76.91 | 2.27(2.17-2.37) |
| Federated States of Micronesia | Cirrhosis due to other causes | 0.11 | 0.12 | 9.09 | 0.10(0.07-0.12) |
| Fiji | Cirrhosis | 139.45 | 222.09 | 59.26 | 1.09(1.07-1.12) |
| Fiji | Cirrhosis due to hepatitis B | 45.92 | 47.30 | 3.01 | -0.36(-0.43--0.28) |
| Fiji | Cirrhosis due to hepatitis C | 8.20 | 9.09 | 10.85 | -0.16(-0.21--0.11) |
| Fiji | Cirrhosis due to alcohol use | 0.36 | 0.89 | 147.22 | 2.76(2.63-2.89) |
| Fiji | Cirrhosis due to NASH | 84.41 | 164.00 | 94.29 | 1.76(1.72-1.79) |
| Fiji | Cirrhosis due to other causes | 0.56 | 0.82 | 46.43 | 0.67(0.54-0.79) |
| Finland | Cirrhosis | 408.28 | 708.11 | 73.44 | 1.79(1.65-1.93) |
| Finland | Cirrhosis due to hepatitis B | 54.32 | 61.25 | 12.76 | 0.08(0.05-0.11) |
| Finland | Cirrhosis due to hepatitis C | 32.46 | 38.29 | 17.96 | 0.55(0.39-0.71) |
| Finland | Cirrhosis due to alcohol use | 27.44 | 51.23 | 86.70 | 2.04(1.77-2.30) |
| Finland | Cirrhosis due to NASH | 282.35 | 537.50 | 90.37 | 2.14(1.98-2.30) |
| Finland | Cirrhosis due to other causes | 11.71 | 19.85 | 69.51 | 1.61(1.48-1.74) |
| France | Cirrhosis | 5485.99 | 7871.59 | 43.49 | 0.96(0.86-1.05) |
| France | Cirrhosis due to hepatitis B | 757.59 | 680.95 | -10.12 | -0.71(-0.83--0.59) |
| France | Cirrhosis due to hepatitis C | 466.92 | 432.95 | -7.28 | -0.56(-0.66--0.46) |
| France | Cirrhosis due to alcohol use | 581.76 | 577.45 | -0.74 | -0.45(-0.54--0.36) |
| France | Cirrhosis due to NASH | 3514.81 | 5977.42 | 70.06 | 1.57(1.43-1.71) |
| France | Cirrhosis due to other causes | 164.91 | 202.83 | 22.99 | 0.31(0.26-0.35) |
| Gabon | Cirrhosis | 224.53 | 350.51 | 56.11 | -0.46(-0.52--0.40) |
| Gabon | Cirrhosis due to hepatitis B | 113.26 | 133.41 | 17.79 | -1.65(-1.77--1.53) |
| Gabon | Cirrhosis due to hepatitis C | 49.80 | 60.25 | 20.98 | -1.35(-1.46--1.24) |
| Gabon | Cirrhosis due to alcohol use | 1.92 | 3.43 | 78.65 | 0.10(-0.07-0.27) |
| Gabon | Cirrhosis due to NASH | 56.16 | 147.58 | 162.78 | 1.69(1.63-1.74) |
| Gabon | Cirrhosis due to other causes | 3.40 | 5.83 | 71.47 | -0.06(-0.13-0.01) |
| Georgia | Cirrhosis | 1027.37 | 776.87 | -24.38 | 0.45(0.42-0.48) |
| Georgia | Cirrhosis due to hepatitis B | 361.32 | 203.17 | -43.77 | -0.84(-0.92--0.76) |
| Georgia | Cirrhosis due to hepatitis C | 231.79 | 154.34 | -33.41 | 0.06(0.01-0.11) |
| Georgia | Cirrhosis due to alcohol use | 24.67 | 21.43 | -13.13 | 0.91(0.70-1.11) |
| Georgia | Cirrhosis due to NASH | 398.16 | 389.96 | -2.06 | 1.55(1.48-1.61) |
| Georgia | Cirrhosis due to other causes | 11.43 | 7.98 | -30.18 | -0.06(-0.17-0.06) |
| Germany | Cirrhosis | 7627.20 | 11561.00 | 51.58 | 1.47(1.35-1.58) |
| Germany | Cirrhosis due to hepatitis B | 705.75 | 644.55 | -8.67 | -0.44(-0.50--0.39) |
| Germany | Cirrhosis due to hepatitis C | 415.20 | 450.85 | 8.59 | 0.07(-0.03-0.16) |
| Germany | Cirrhosis due to alcohol use | 805.67 | 1017.36 | 26.28 | 0.52(0.40-0.64) |
| Germany | Cirrhosis due to NASH | 5468.84 | 9120.83 | 66.78 | 1.87(1.74-2.01) |
| Germany | Cirrhosis due to other causes | 231.73 | 327.41 | 41.29 | 1.01(0.92-1.09) |
| Ghana | Cirrhosis | 3475.94 | 6547.05 | 88.35 | -0.51(-0.63--0.39) |
| Ghana | Cirrhosis due to hepatitis B | 2213.97 | 3327.20 | 50.28 | -1.41(-1.60--1.22) |
| Ghana | Cirrhosis due to hepatitis C | 562.14 | 940.55 | 67.32 | -0.93(-1.03--0.82) |
| Ghana | Cirrhosis due to alcohol use | 24.96 | 65.26 | 161.46 | 0.70(0.58-0.82) |
| Ghana | Cirrhosis due to NASH | 613.15 | 2083.09 | 239.74 | 1.88(1.84-1.93) |
| Ghana | Cirrhosis due to other causes | 61.72 | 130.94 | 112.15 | 0.11(0.07-0.15) |
| Greece | Cirrhosis | 1357.45 | 1768.45 | 30.28 | 1.05(0.98-1.12) |
| Greece | Cirrhosis due to hepatitis B | 288.28 | 247.65 | -14.09 | -0.77(-1.00--0.54) |
| Greece | Cirrhosis due to hepatitis C | 92.96 | 77.80 | -16.31 | -0.58(-0.67--0.49) |
| Greece | Cirrhosis due to alcohol use | 76.40 | 80.70 | 5.63 | 0.14(-0.06-0.34) |
| Greece | Cirrhosis due to NASH | 872.91 | 1332.68 | 52.67 | 1.69(1.52-1.86) |
| Greece | Cirrhosis due to other causes | 26.90 | 29.62 | 10.11 | 0.23(0.13-0.33) |
| Greenland | Cirrhosis | 4.17 | 5.72 | 37.17 | 1.25(1.22-1.29) |
| Greenland | Cirrhosis due to hepatitis B | 0.57 | 0.62 | 8.77 | 0.20(0.15-0.24) |
| Greenland | Cirrhosis due to hepatitis C | 0.35 | 0.36 | 2.86 | 0.22(0.12-0.32) |
| Greenland | Cirrhosis due to alcohol use | 0.05 | 0.08 | 60.00 | 1.88(1.80-1.96) |
| Greenland | Cirrhosis due to NASH | 3.16 | 4.60 | 45.57 | 1.51(1.46-1.56) |
| Greenland | Cirrhosis due to other causes | 0.04 | 0.06 | 50.00 | 1.19(1.08-1.30) |
| Grenada | Cirrhosis | 8.64 | 18.92 | 118.98 | 2.21(2.14-2.28) |
| Grenada | Cirrhosis due to hepatitis B | 1.05 | 1.17 | 11.43 | -0.58(-0.81--0.35) |
| Grenada | Cirrhosis due to hepatitis C | 0.85 | 0.85 | 0.00 | -0.76(-0.92--0.60) |
| Grenada | Cirrhosis due to alcohol use | 0.23 | 0.47 | 104.35 | 1.75(1.68-1.83) |
| Grenada | Cirrhosis due to NASH | 6.37 | 16.20 | 154.32 | 2.80(2.71-2.90) |
| Grenada | Cirrhosis due to other causes | 0.14 | 0.24 | 71.43 | 0.84(0.72-0.95) |
| Guam | Cirrhosis | 31.16 | 49.55 | 59.02 | 1.05(0.97-1.12) |
| Guam | Cirrhosis due to hepatitis B | 13.28 | 15.52 | 16.87 | -0.13(-0.16--0.11) |
| Guam | Cirrhosis due to hepatitis C | 1.45 | 1.78 | 22.76 | 0.02(-0.07-0.11) |
| Guam | Cirrhosis due to alcohol use | 0.15 | 0.25 | 66.67 | 1.18(1.09-1.27) |
| Guam | Cirrhosis due to NASH | 16.17 | 31.84 | 96.91 | 1.88(1.78-1.97) |
| Guam | Cirrhosis due to other causes | 0.12 | 0.16 | 33.33 | 0.38(0.35-0.41) |
| Guatemala | Cirrhosis | 1157.26 | 3268.25 | 182.41 | 1.09(0.99-1.19) |
| Guatemala | Cirrhosis due to hepatitis B | 291.44 | 454.54 | 55.96 | -1.44(-1.69--1.18) |
| Guatemala | Cirrhosis due to hepatitis C | 187.40 | 330.40 | 76.31 | -0.65(-0.71--0.60) |
| Guatemala | Cirrhosis due to alcohol use | 38.44 | 123.53 | 221.36 | 1.24(1.13-1.35) |
| Guatemala | Cirrhosis due to NASH | 620.71 | 2318.99 | 273.60 | 2.25(2.18-2.31) |
| Guatemala | Cirrhosis due to other causes | 19.27 | 40.78 | 111.62 | -0.28(-0.38--0.18) |
| Guinea | Cirrhosis | 1587.90 | 2672.99 | 68.33 | -0.62(-0.72--0.52) |
| Guinea | Cirrhosis due to hepatitis B | 1007.30 | 1591.19 | 57.97 | -0.93(-1.09--0.77) |
| Guinea | Cirrhosis due to hepatitis C | 247.72 | 377.64 | 52.45 | -0.85(-0.94--0.77) |
| Guinea | Cirrhosis due to alcohol use | 10.22 | 19.37 | 89.53 | -0.06(-0.14-0.02) |
| Guinea | Cirrhosis due to NASH | 294.10 | 629.79 | 114.14 | 0.43(0.37-0.49) |
| Guinea | Cirrhosis due to other causes | 28.56 | 55.00 | 92.58 | -0.03(-0.17-0.12) |
| Guinea-Bissau | Cirrhosis | 259.15 | 427.87 | 65.11 | -0.50(-0.62--0.38) |
| Guinea-Bissau | Cirrhosis due to hepatitis B | 165.23 | 234.56 | 41.96 | -1.19(-1.40--0.97) |
| Guinea-Bissau | Cirrhosis due to hepatitis C | 38.97 | 53.09 | 36.23 | -1.04(-1.12--0.96) |
| Guinea-Bissau | Cirrhosis due to alcohol use | 1.65 | 3.17 | 92.12 | 0.22(0.08-0.36) |
| Guinea-Bissau | Cirrhosis due to NASH | 48.38 | 128.52 | 165.65 | 1.53(1.44-1.62) |
| Guinea-Bissau | Cirrhosis due to other causes | 4.92 | 8.54 | 73.58 | -0.11(-0.19--0.04) |
| Guyana | Cirrhosis | 76.79 | 108.08 | 40.75 | 1.57(1.53-1.60) |
| Guyana | Cirrhosis due to hepatitis B | 11.24 | 9.14 | -18.68 | -0.89(-1.06--0.71) |
| Guyana | Cirrhosis due to hepatitis C | 8.21 | 6.62 | -19.37 | -0.54(-0.64--0.43) |
| Guyana | Cirrhosis due to alcohol use | 2.53 | 3.41 | 34.78 | 1.33(1.22-1.45) |
| Guyana | Cirrhosis due to NASH | 53.43 | 87.21 | 63.22 | 2.19(2.12-2.25) |
| Guyana | Cirrhosis due to other causes | 1.38 | 1.70 | 23.19 | 0.97(0.94-1.00) |
| Haiti | Cirrhosis | 546.87 | 1362.34 | 149.12 | 1.20(1.17-1.24) |
| Haiti | Cirrhosis due to hepatitis B | 88.53 | 142.46 | 60.92 | -0.38(-0.46--0.30) |
| Haiti | Cirrhosis due to hepatitis C | 73.34 | 111.75 | 52.37 | -0.79(-0.83--0.75) |
| Haiti | Cirrhosis due to alcohol use | 13.16 | 38.84 | 195.14 | 1.57(1.48-1.66) |
| Haiti | Cirrhosis due to NASH | 362.33 | 1044.78 | 188.35 | 1.79(1.74-1.84) |
| Haiti | Cirrhosis due to other causes | 9.51 | 24.51 | 157.73 | 1.11(1.06-1.16) |
| Honduras | Cirrhosis | 663.36 | 1836.19 | 176.80 | 1.20(1.13-1.27) |
| Honduras | Cirrhosis due to hepatitis B | 165.05 | 231.47 | 40.24 | -1.56(-1.86--1.26) |
| Honduras | Cirrhosis due to hepatitis C | 103.96 | 187.15 | 80.02 | -0.44(-0.45--0.42) |
| Honduras | Cirrhosis due to alcohol use | 20.73 | 68.48 | 230.34 | 1.82(1.72-1.92) |
| Honduras | Cirrhosis due to NASH | 361.82 | 1326.51 | 266.62 | 2.31(2.25-2.38) |
| Honduras | Cirrhosis due to other causes | 11.80 | 22.58 | 91.36 | -0.25(-0.29--0.21) |
| Hungary | Cirrhosis | 1804.23 | 1896.76 | 5.13 | 0.41(0.39-0.43) |
| Hungary | Cirrhosis due to hepatitis B | 427.33 | 417.86 | -2.22 | 0.19(0.16-0.22) |
| Hungary | Cirrhosis due to hepatitis C | 177.51 | 103.77 | -41.54 | -2.00(-2.14--1.86) |
| Hungary | Cirrhosis due to alcohol use | 165.52 | 117.66 | -28.91 | -1.06(-1.27--0.85) |
| Hungary | Cirrhosis due to NASH | 994.00 | 1230.04 | 23.75 | 1.06(1.03-1.09) |
| Hungary | Cirrhosis due to other causes | 39.87 | 27.43 | -31.20 | -1.18(-1.28--1.08) |
| Iceland | Cirrhosis | 22.54 | 45.94 | 103.82 | 1.77(1.68-1.85) |
| Iceland | Cirrhosis due to hepatitis B | 2.05 | 2.65 | 29.27 | -0.05(-0.08--0.02) |
| Iceland | Cirrhosis due to hepatitis C | 1.14 | 1.39 | 21.93 | -0.29(-0.31--0.26) |
| Iceland | Cirrhosis due to alcohol use | 0.60 | 1.68 | 180.00 | 2.60(2.53-2.67) |
| Iceland | Cirrhosis due to NASH | 18.24 | 39.07 | 114.20 | 1.99(1.88-2.10) |
| Iceland | Cirrhosis due to other causes | 0.51 | 1.15 | 125.49 | 1.92(1.88-1.96) |
| India | Cirrhosis | 87407.84 | 170020.21 | 94.51 | 0.79(0.75-0.83) |
| India | Cirrhosis due to hepatitis B | 34352.81 | 55874.72 | 62.65 | 0.19(0.14-0.24) |
| India | Cirrhosis due to hepatitis C | 9275.16 | 11858.66 | 27.85 | -0.81(-0.99--0.62) |
| India | Cirrhosis due to alcohol use | 1039.08 | 2369.53 | 128.04 | 1.24(1.10-1.39) |
| India | Cirrhosis due to NASH | 41743.08 | 98235.85 | 135.33 | 1.48(1.43-1.53) |
| India | Cirrhosis due to other causes | 997.70 | 1681.45 | 68.53 | 0.10(0.03-0.18) |
| Indonesia | Cirrhosis | 33039.60 | 64612.93 | 95.56 | 1.35(1.31-1.38) |
| Indonesia | Cirrhosis due to hepatitis B | 10669.62 | 14643.27 | 37.24 | -0.07(-0.09--0.05) |
| Indonesia | Cirrhosis due to hepatitis C | 4159.69 | 5442.92 | 30.85 | 0.04(-0.05-0.12) |
| Indonesia | Cirrhosis due to alcohol use | 86.84 | 150.19 | 72.95 | 0.74(0.64-0.84) |
| Indonesia | Cirrhosis due to NASH | 17993.72 | 44245.18 | 145.89 | 2.22(2.17-2.27) |
| Indonesia | Cirrhosis due to other causes | 129.74 | 131.37 | 1.26 | -1.31(-1.40--1.21) |
| Iran | Cirrhosis | 7723.05 | 20909.02 | 170.74 | 2.58(2.51-2.65) |
| Iran | Cirrhosis due to hepatitis B | 1487.90 | 2170.81 | 45.90 | 0.28(0.21-0.34) |
| Iran | Cirrhosis due to hepatitis C | 241.06 | 569.87 | 136.40 | 2.20(2.08-2.32) |
| Iran | Cirrhosis due to alcohol use | 12.16 | 35.70 | 193.59 | 3.12(2.95-3.28) |
| Iran | Cirrhosis due to NASH | 5840.88 | 17971.81 | 207.69 | 3.06(2.99-3.12) |
| Iran | Cirrhosis due to other causes | 141.06 | 160.83 | 14.02 | -0.88(-1.00--0.77) |
| Iraq | Cirrhosis | 3057.31 | 9094.54 | 197.47 | 0.69(0.62-0.76) |
| Iraq | Cirrhosis due to hepatitis B | 842.54 | 1747.44 | 107.40 | -0.74(-0.77--0.71) |
| Iraq | Cirrhosis due to hepatitis C | 197.50 | 371.25 | 87.97 | -1.05(-1.14--0.96) |
| Iraq | Cirrhosis due to alcohol use | 5.96 | 18.30 | 207.05 | 0.84(0.62-1.06) |
| Iraq | Cirrhosis due to NASH | 1961.08 | 6834.28 | 248.50 | 1.33(1.24-1.42) |
| Iraq | Cirrhosis due to other causes | 50.24 | 123.26 | 145.34 | -0.02(-0.10-0.07) |
| Ireland | Cirrhosis | 343.51 | 687.15 | 100.04 | 1.46(1.31-1.60) |
| Ireland | Cirrhosis due to hepatitis B | 31.55 | 35.35 | 12.04 | -0.93(-1.17--0.69) |
| Ireland | Cirrhosis due to hepatitis C | 21.79 | 27.47 | 26.07 | -0.01(-0.10-0.08) |
| Ireland | Cirrhosis due to alcohol use | 12.35 | 34.69 | 180.89 | 2.68(2.50-2.86) |
| Ireland | Cirrhosis due to NASH | 270.95 | 576.29 | 112.69 | 1.69(1.53-1.85) |
| Ireland | Cirrhosis due to other causes | 6.87 | 13.36 | 94.47 | 1.25(1.21-1.29) |
| Israel | Cirrhosis | 587.31 | 1565.87 | 166.62 | 1.45(1.35-1.54) |
| Israel | Cirrhosis due to hepatitis B | 49.40 | 74.35 | 50.51 | -0.74(-0.95--0.53) |
| Israel | Cirrhosis due to hepatitis C | 29.17 | 47.74 | 63.66 | -0.34(-0.38--0.31) |
| Israel | Cirrhosis due to alcohol use | 17.65 | 43.71 | 147.65 | 1.06(0.98-1.14) |
| Israel | Cirrhosis due to NASH | 479.02 | 1372.38 | 186.50 | 1.72(1.59-1.85) |
| Israel | Cirrhosis due to other causes | 12.07 | 27.69 | 129.41 | 0.82(0.74-0.90) |
| Italy | Cirrhosis | 10367.94 | 13771.87 | 32.83 | 0.73(0.64-0.83) |
| Italy | Cirrhosis due to hepatitis B | 1036.05 | 1101.23 | 6.29 | 0.07(0.00-0.14) |
| Italy | Cirrhosis due to hepatitis C | 1660.14 | 1390.93 | -16.22 | -1.09(-1.30--0.88) |
| Italy | Cirrhosis due to alcohol use | 360.65 | 336.51 | -6.69 | -0.38(-0.49--0.28) |
| Italy | Cirrhosis due to NASH | 7095.03 | 10723.62 | 51.14 | 1.20(1.07-1.34) |
| Italy | Cirrhosis due to other causes | 216.08 | 219.58 | 1.62 | -0.09(-0.15--0.04) |
| Jamaica | Cirrhosis | 244.19 | 447.40 | 83.22 | 1.81(1.74-1.88) |
| Jamaica | Cirrhosis due to hepatitis B | 20.30 | 19.36 | -4.63 | -1.06(-1.23--0.88) |
| Jamaica | Cirrhosis due to hepatitis C | 17.18 | 17.49 | 1.80 | -0.63(-0.72--0.54) |
| Jamaica | Cirrhosis due to alcohol use | 3.85 | 9.16 | 137.92 | 2.25(1.99-2.51) |
| Jamaica | Cirrhosis due to NASH | 199.39 | 395.86 | 98.54 | 2.17(2.07-2.27) |
| Jamaica | Cirrhosis due to other causes | 3.47 | 5.54 | 59.65 | 0.95(0.82-1.07) |
| Japan | Cirrhosis | 17428.92 | 21105.86 | 21.10 | 0.66(0.62-0.69) |
| Japan | Cirrhosis due to hepatitis B | 4567.46 | 4350.01 | -4.76 | -0.46(-0.53--0.39) |
| Japan | Cirrhosis due to hepatitis C | 3120.19 | 3098.20 | -0.70 | 0.16(0.05-0.27) |
| Japan | Cirrhosis due to alcohol use | 559.02 | 593.91 | 6.24 | 0.40(0.27-0.52) |
| Japan | Cirrhosis due to NASH | 8518.47 | 12475.42 | 46.45 | 1.37(1.29-1.45) |
| Japan | Cirrhosis due to other causes | 663.77 | 588.32 | -11.37 | -0.45(-0.55--0.34) |
| Jordan | Cirrhosis | 670.54 | 2666.06 | 297.60 | 1.19(1.08-1.30) |
| Jordan | Cirrhosis due to hepatitis B | 242.63 | 500.46 | 106.26 | -1.19(-1.45--0.93) |
| Jordan | Cirrhosis due to hepatitis C | 26.71 | 82.88 | 210.30 | 0.17(0.03-0.32) |
| Jordan | Cirrhosis due to alcohol use | 1.40 | 6.94 | 395.71 | 1.96(1.64-2.28) |
| Jordan | Cirrhosis due to NASH | 386.04 | 2035.24 | 427.21 | 2.22(2.18-2.25) |
| Jordan | Cirrhosis due to other causes | 13.76 | 40.54 | 194.62 | 0.12(0.00-0.24) |
| Kazakhstan | Cirrhosis | 3124.43 | 3487.42 | 11.62 | 0.14(0.04-0.24) |
| Kazakhstan | Cirrhosis due to hepatitis B | 1288.52 | 1158.43 | -10.10 | -0.82(-1.08--0.56) |
| Kazakhstan | Cirrhosis due to hepatitis C | 757.14 | 766.90 | 1.29 | -0.10(-0.19--0.01) |
| Kazakhstan | Cirrhosis due to alcohol use | 54.00 | 115.89 | 114.61 | 3.33(3.06-3.60) |
| Kazakhstan | Cirrhosis due to NASH | 989.39 | 1395.06 | 41.00 | 1.01(0.96-1.05) |
| Kazakhstan | Cirrhosis due to other causes | 35.39 | 51.14 | 44.50 | 1.42(1.31-1.53) |
| Kenya | Cirrhosis | 2362.75 | 5921.26 | 150.61 | 0.70(0.66-0.73) |
| Kenya | Cirrhosis due to hepatitis B | 988.91 | 2041.85 | 106.47 | 0.06(-0.01-0.13) |
| Kenya | Cirrhosis due to hepatitis C | 311.55 | 719.23 | 130.86 | 0.42(0.38-0.47) |
| Kenya | Cirrhosis due to alcohol use | 46.66 | 116.33 | 149.31 | 0.58(0.50-0.66) |
| Kenya | Cirrhosis due to NASH | 910.26 | 2812.59 | 208.99 | 1.43(1.38-1.48) |
| Kenya | Cirrhosis due to other causes | 105.38 | 231.26 | 119.45 | 0.07(0.00-0.14) |
| Kiribati | Cirrhosis | 18.81 | 33.28 | 76.93 | 0.56(0.49-0.64) |
| Kiribati | Cirrhosis due to hepatitis B | 8.93 | 11.64 | 30.35 | -0.56(-0.69--0.44) |
| Kiribati | Cirrhosis due to hepatitis C | 1.28 | 1.86 | 45.31 | -0.31(-0.34--0.28) |
| Kiribati | Cirrhosis due to alcohol use | 0.05 | 0.12 | 140.00 | 1.45(1.30-1.60) |
| Kiribati | Cirrhosis due to NASH | 8.47 | 19.51 | 130.34 | 1.55(1.44-1.65) |
| Kiribati | Cirrhosis due to other causes | 0.07 | 0.14 | 100.00 | 0.56(0.50-0.62) |
| Kuwait | Cirrhosis | 355.54 | 1305.01 | 267.05 | 1.50(1.47-1.53) |
| Kuwait | Cirrhosis due to hepatitis B | 65.09 | 141.31 | 117.10 | -0.46(-0.49--0.42) |
| Kuwait | Cirrhosis due to hepatitis C | 9.23 | 29.00 | 214.19 | 0.82(0.71-0.93) |
| Kuwait | Cirrhosis due to alcohol use | 0.65 | 2.83 | 335.38 | 2.12(1.98-2.26) |
| Kuwait | Cirrhosis due to NASH | 276.97 | 1123.56 | 305.66 | 1.87(1.83-1.91) |
| Kuwait | Cirrhosis due to other causes | 3.61 | 8.31 | 130.19 | -0.07(-0.13--0.01) |
| Kyrgyzstan | Cirrhosis | 921.30 | 1313.12 | 42.53 | -0.03(-0.04--0.01) |
| Kyrgyzstan | Cirrhosis due to hepatitis B | 384.44 | 476.64 | 23.98 | -0.75(-0.86--0.64) |
| Kyrgyzstan | Cirrhosis due to hepatitis C | 244.70 | 305.57 | 24.88 | -0.39(-0.64--0.14) |
| Kyrgyzstan | Cirrhosis due to alcohol use | 16.98 | 33.66 | 98.23 | 1.91(1.59-2.24) |
| Kyrgyzstan | Cirrhosis due to NASH | 262.45 | 476.45 | 81.54 | 0.98(0.89-1.08) |
| Kyrgyzstan | Cirrhosis due to other causes | 12.73 | 20.81 | 63.47 | 0.76(0.61-0.91) |
| Laos | Cirrhosis | 699.64 | 1208.49 | 72.73 | -0.01(-0.10-0.08) |
| Laos | Cirrhosis due to hepatitis B | 342.68 | 434.32 | 26.74 | -1.38(-1.51--1.24) |
| Laos | Cirrhosis due to hepatitis C | 122.16 | 162.93 | 33.37 | -0.87(-0.92--0.82) |
| Laos | Cirrhosis due to alcohol use | 1.97 | 5.96 | 202.54 | 2.12(1.89-2.34) |
| Laos | Cirrhosis due to NASH | 230.79 | 600.97 | 160.40 | 1.77(1.62-1.92) |
| Laos | Cirrhosis due to other causes | 2.04 | 4.31 | 111.27 | 0.81(0.77-0.85) |
| Latvia | Cirrhosis | 387.28 | 336.57 | -13.09 | 0.82(0.73-0.92) |
| Latvia | Cirrhosis due to hepatitis B | 110.00 | 60.99 | -44.55 | -1.16(-1.39--0.92) |
| Latvia | Cirrhosis due to hepatitis C | 40.64 | 32.62 | -19.73 | 0.45(0.29-0.62) |
| Latvia | Cirrhosis due to alcohol use | 12.98 | 16.23 | 25.04 | 2.58(2.40-2.76) |
| Latvia | Cirrhosis due to NASH | 216.87 | 221.01 | 1.91 | 1.52(1.43-1.61) |
| Latvia | Cirrhosis due to other causes | 6.80 | 5.71 | -16.03 | 0.74(0.63-0.85) |
| Lebanon | Cirrhosis | 654.57 | 1890.96 | 188.89 | 1.23(1.18-1.28) |
| Lebanon | Cirrhosis due to hepatitis B | 209.94 | 362.83 | 72.83 | -0.76(-0.92--0.61) |
| Lebanon | Cirrhosis due to hepatitis C | 27.23 | 59.39 | 118.11 | 0.20(0.00-0.40) |
| Lebanon | Cirrhosis due to alcohol use | 1.87 | 5.66 | 202.67 | 1.50(1.29-1.71) |
| Lebanon | Cirrhosis due to NASH | 403.94 | 1439.14 | 256.28 | 2.04(2.00-2.09) |
| Lebanon | Cirrhosis due to other causes | 11.59 | 23.94 | 106.56 | 0.04(-0.04-0.13) |
| Lesotho | Cirrhosis | 326.57 | 335.36 | 2.69 | -0.34(-0.42--0.26) |
| Lesotho | Cirrhosis due to hepatitis B | 188.04 | 141.42 | -24.79 | -1.67(-1.83--1.51) |
| Lesotho | Cirrhosis due to hepatitis C | 34.23 | 31.84 | -6.98 | -0.43(-0.52--0.35) |
| Lesotho | Cirrhosis due to alcohol use | 2.48 | 3.39 | 36.69 | 0.79(0.70-0.88) |
| Lesotho | Cirrhosis due to NASH | 95.80 | 152.12 | 58.79 | 1.51(1.48-1.53) |
| Lesotho | Cirrhosis due to other causes | 6.03 | 6.58 | 9.12 | 0.00(-0.06-0.06) |
| Liberia | Cirrhosis | 521.79 | 1145.35 | 119.50 | -0.51(-0.60--0.42) |
| Liberia | Cirrhosis due to hepatitis B | 313.65 | 622.69 | 98.53 | -0.95(-1.15--0.75) |
| Liberia | Cirrhosis due to hepatitis C | 73.81 | 137.21 | 85.90 | -1.32(-1.47--1.17) |
| Liberia | Cirrhosis due to alcohol use | 4.48 | 9.73 | 117.19 | -0.55(-0.78--0.31) |
| Liberia | Cirrhosis due to NASH | 119.92 | 353.01 | 194.37 | 0.91(0.71-1.11) |
| Liberia | Cirrhosis due to other causes | 9.93 | 22.71 | 128.70 | -0.45(-0.56--0.35) |
| Libya | Cirrhosis | 741.68 | 1897.15 | 155.79 | 1.72(1.61-1.83) |
| Libya | Cirrhosis due to hepatitis B | 203.44 | 294.58 | 44.80 | -0.62(-0.89--0.36) |
| Libya | Cirrhosis due to hepatitis C | 35.85 | 67.33 | 87.81 | 0.43(0.26-0.59) |
| Libya | Cirrhosis due to alcohol use | 1.66 | 5.05 | 204.22 | 2.38(2.13-2.63) |
| Libya | Cirrhosis due to NASH | 488.64 | 1511.43 | 209.31 | 2.50(2.44-2.56) |
| Libya | Cirrhosis due to other causes | 12.09 | 18.76 | 55.17 | -0.33(-0.40--0.27) |
| Lithuania | Cirrhosis | 505.94 | 495.97 | -1.97 | 1.13(1.04-1.21) |
| Lithuania | Cirrhosis due to hepatitis B | 142.14 | 84.03 | -40.88 | -1.06(-1.27--0.85) |
| Lithuania | Cirrhosis due to hepatitis C | 61.21 | 53.98 | -11.81 | 0.86(0.64-1.09) |
| Lithuania | Cirrhosis due to alcohol use | 19.91 | 29.83 | 49.82 | 3.20(2.92-3.48) |
| Lithuania | Cirrhosis due to NASH | 271.33 | 318.13 | 17.25 | 1.84(1.76-1.93) |
| Lithuania | Cirrhosis due to other causes | 11.35 | 10.00 | -11.89 | 0.70(0.56-0.83) |
| Luxembourg | Cirrhosis | 42.63 | 84.19 | 97.49 | 0.96(0.92-1.00) |
| Luxembourg | Cirrhosis due to hepatitis B | 4.73 | 6.50 | 37.42 | -0.54(-0.69--0.39) |
| Luxembourg | Cirrhosis due to hepatitis C | 3.10 | 3.87 | 24.84 | -0.76(-0.78--0.74) |
| Luxembourg | Cirrhosis due to alcohol use | 4.47 | 8.33 | 86.35 | 0.84(0.79-0.90) |
| Luxembourg | Cirrhosis due to NASH | 28.85 | 62.97 | 118.27 | 1.34(1.27-1.42) |
| Luxembourg | Cirrhosis due to other causes | 1.48 | 2.52 | 70.27 | 0.28(0.23-0.33) |
| Macedonia | Cirrhosis | 248.05 | 375.65 | 51.44 | 1.26(1.21-1.30) |
| Macedonia | Cirrhosis due to hepatitis B | 50.21 | 47.88 | -4.64 | -0.78(-0.92--0.64) |
| Macedonia | Cirrhosis due to hepatitis C | 20.69 | 20.22 | -2.27 | -0.36(-0.41--0.31) |
| Macedonia | Cirrhosis due to alcohol use | 10.54 | 18.24 | 73.06 | 1.56(1.38-1.74) |
| Macedonia | Cirrhosis due to NASH | 160.56 | 283.20 | 76.38 | 1.93(1.87-1.98) |
| Macedonia | Cirrhosis due to other causes | 6.04 | 6.12 | 1.32 | -0.39(-0.50--0.29) |
| Madagascar | Cirrhosis | 2025.21 | 3892.34 | 92.19 | -0.67(-0.79--0.56) |
| Madagascar | Cirrhosis due to hepatitis B | 1424.52 | 2481.53 | 74.20 | -1.12(-1.28--0.96) |
| Madagascar | Cirrhosis due to hepatitis C | 120.82 | 222.57 | 84.22 | -0.62(-0.73--0.52) |
| Madagascar | Cirrhosis due to alcohol use | 20.07 | 47.78 | 138.07 | 0.30(0.20-0.40) |
| Madagascar | Cirrhosis due to NASH | 411.22 | 1031.26 | 150.78 | 0.52(0.44-0.60) |
| Madagascar | Cirrhosis due to other causes | 48.59 | 109.19 | 124.72 | 0.08(0.01-0.15) |
| Malawi | Cirrhosis | 1455.65 | 2344.33 | 61.05 | -0.57(-0.70--0.44) |
| Malawi | Cirrhosis due to hepatitis B | 821.90 | 1086.59 | 32.20 | -1.36(-1.58--1.14) |
| Malawi | Cirrhosis due to hepatitis C | 139.16 | 208.98 | 50.17 | -0.89(-1.03--0.74) |
| Malawi | Cirrhosis due to alcohol use | 17.78 | 33.50 | 88.41 | -0.12(-0.25-0.00) |
| Malawi | Cirrhosis due to NASH | 433.11 | 933.96 | 115.64 | 0.66(0.59-0.73) |
| Malawi | Cirrhosis due to other causes | 43.70 | 81.29 | 86.02 | 0.05(0.01-0.10) |
| Malaysia | Cirrhosis | 2557.80 | 7141.49 | 179.20 | 1.87(1.83-1.91) |
| Malaysia | Cirrhosis due to hepatitis B | 310.80 | 584.90 | 88.19 | 0.39(0.35-0.42) |
| Malaysia | Cirrhosis due to hepatitis C | 240.23 | 365.60 | 52.19 | -0.35(-0.42--0.27) |
| Malaysia | Cirrhosis due to alcohol use | 5.75 | 21.82 | 279.48 | 2.77(2.57-2.96) |
| Malaysia | Cirrhosis due to NASH | 1994.63 | 6154.51 | 208.55 | 2.24(2.20-2.28) |
| Malaysia | Cirrhosis due to other causes | 6.40 | 14.66 | 129.06 | 0.98(0.91-1.05) |
| Maldives | Cirrhosis | 29.53 | 107.32 | 263.43 | 2.33(2.13-2.54) |
| Maldives | Cirrhosis due to hepatitis B | 9.09 | 16.26 | 78.88 | -0.33(-0.58--0.08) |
| Maldives | Cirrhosis due to hepatitis C | 3.50 | 5.44 | 55.43 | -1.05(-1.10--1.00) |
| Maldives | Cirrhosis due to alcohol use | 0.06 | 0.32 | 433.33 | 3.66(3.13-4.19) |
| Maldives | Cirrhosis due to NASH | 16.78 | 85.08 | 407.03 | 3.62(3.47-3.77) |
| Maldives | Cirrhosis due to other causes | 0.10 | 0.22 | 120.00 | 0.12(0.08-0.15) |
| Mali | Cirrhosis | 2289.80 | 4804.44 | 109.82 | -0.55(-0.65--0.46) |
| Mali | Cirrhosis due to hepatitis B | 1436.01 | 2770.20 | 92.91 | -0.93(-1.09--0.77) |
| Mali | Cirrhosis due to hepatitis C | 370.82 | 656.03 | 76.91 | -1.04(-1.06--1.01) |
| Mali | Cirrhosis due to alcohol use | 14.46 | 32.28 | 123.24 | -0.44(-0.61--0.26) |
| Mali | Cirrhosis due to NASH | 420.83 | 1233.66 | 193.15 | 0.83(0.81-0.84) |
| Mali | Cirrhosis due to other causes | 47.68 | 112.27 | 135.47 | -0.20(-0.34--0.06) |
| Malta | Cirrhosis | 37.46 | 70.56 | 88.36 | 1.86(1.73-1.98) |
| Malta | Cirrhosis due to hepatitis B | 3.62 | 3.71 | 2.49 | -0.69(-0.82--0.55) |
| Malta | Cirrhosis due to hepatitis C | 2.33 | 2.54 | 9.01 | -0.19(-0.22--0.16) |
| Malta | Cirrhosis due to alcohol use | 1.43 | 2.19 | 53.15 | 0.97(0.91-1.02) |
| Malta | Cirrhosis due to NASH | 29.52 | 61.30 | 107.66 | 2.25(2.09-2.41) |
| Malta | Cirrhosis due to other causes | 0.57 | 0.82 | 43.86 | 0.71(0.67-0.74) |
| Marshall Islands | Cirrhosis | 10.63 | 16.44 | 54.66 | 0.98(0.90-1.06) |
| Marshall Islands | Cirrhosis due to hepatitis B | 5.47 | 5.87 | 7.31 | -0.38(-0.48--0.28) |
| Marshall Islands | Cirrhosis due to hepatitis C | 0.69 | 0.78 | 13.04 | -0.29(-0.31--0.27) |
| Marshall Islands | Cirrhosis due to alcohol use | 0.03 | 0.06 | 100.00 | 2.49(2.44-2.53) |
| Marshall Islands | Cirrhosis due to NASH | 4.39 | 9.65 | 119.82 | 2.29(2.25-2.34) |
| Marshall Islands | Cirrhosis due to other causes | 0.05 | 0.06 | 20.00 | -0.05(-0.08--0.02) |
| Mauritania | Cirrhosis | 475.92 | 876.37 | 84.14 | -0.21(-0.27--0.15) |
| Mauritania | Cirrhosis due to hepatitis B | 263.96 | 404.31 | 53.17 | -0.97(-1.09--0.86) |
| Mauritania | Cirrhosis due to hepatitis C | 64.57 | 90.12 | 39.57 | -1.10(-1.12--1.08) |
| Mauritania | Cirrhosis due to alcohol use | 2.74 | 5.71 | 108.39 | 0.25(0.12-0.38) |
| Mauritania | Cirrhosis due to NASH | 136.05 | 359.36 | 164.14 | 1.22(1.20-1.25) |
| Mauritania | Cirrhosis due to other causes | 8.60 | 16.87 | 96.16 | 0.04(-0.04-0.12) |
| Mauritius | Cirrhosis | 175.71 | 299.26 | 70.31 | 1.41(1.37-1.45) |
| Mauritius | Cirrhosis due to hepatitis B | 56.96 | 62.76 | 10.18 | -0.38(-0.59--0.17) |
| Mauritius | Cirrhosis due to hepatitis C | 19.20 | 17.54 | -8.65 | -0.99(-1.10--0.89) |
| Mauritius | Cirrhosis due to alcohol use | 0.80 | 1.35 | 68.75 | 1.28(1.12-1.43) |
| Mauritius | Cirrhosis due to NASH | 98.26 | 216.98 | 120.82 | 2.46(2.41-2.51) |
| Mauritius | Cirrhosis due to other causes | 0.49 | 0.62 | 26.53 | 0.32(0.24-0.40) |
| Mexico | Cirrhosis | 12340.94 | 26317.67 | 113.25 | 1.30(1.22-1.38) |
| Mexico | Cirrhosis due to hepatitis B | 3178.54 | 2935.55 | -7.64 | -2.19(-2.59--1.79) |
| Mexico | Cirrhosis due to hepatitis C | 1053.57 | 1705.47 | 61.88 | 0.34(0.32-0.37) |
| Mexico | Cirrhosis due to alcohol use | 613.87 | 1429.37 | 132.85 | 1.58(1.51-1.66) |
| Mexico | Cirrhosis due to NASH | 7268.74 | 19866.67 | 173.32 | 2.29(2.23-2.34) |
| Mexico | Cirrhosis due to other causes | 226.21 | 380.61 | 68.26 | 0.38(0.30-0.45) |
| Moldova | Cirrhosis | 815.34 | 810.25 | -0.62 | 0.78(0.71-0.85) |
| Moldova | Cirrhosis due to hepatitis B | 288.73 | 219.53 | -23.97 | -0.17(-0.34-0.00) |
| Moldova | Cirrhosis due to hepatitis C | 136.54 | 119.95 | -12.15 | 0.23(0.07-0.40) |
| Moldova | Cirrhosis due to alcohol use | 52.37 | 46.61 | -11.00 | 0.74(0.52-0.95) |
| Moldova | Cirrhosis due to NASH | 315.33 | 407.54 | 29.24 | 1.71(1.61-1.81) |
| Moldova | Cirrhosis due to other causes | 22.38 | 16.62 | -25.74 | -0.16(-0.31--0.01) |
| Mongolia | Cirrhosis | 501.92 | 778.08 | 55.02 | 0.29(0.22-0.36) |
| Mongolia | Cirrhosis due to hepatitis B | 223.20 | 308.01 | 38.00 | -0.01(-0.16-0.14) |
| Mongolia | Cirrhosis due to hepatitis C | 168.18 | 220.99 | 31.40 | -0.45(-0.56--0.34) |
| Mongolia | Cirrhosis due to alcohol use | 7.20 | 18.60 | 158.33 | 2.73(2.49-2.97) |
| Mongolia | Cirrhosis due to NASH | 96.02 | 221.41 | 130.59 | 1.72(1.64-1.79) |
| Mongolia | Cirrhosis due to other causes | 7.32 | 9.09 | 24.18 | -0.68(-0.71--0.65) |
| Montenegro | Cirrhosis | 75.33 | 95.72 | 27.07 | 0.87(0.83-0.91) |
| Montenegro | Cirrhosis due to hepatitis B | 14.62 | 12.74 | -12.86 | -0.74(-0.90--0.58) |
| Montenegro | Cirrhosis due to hepatitis C | 5.57 | 5.32 | -4.49 | -0.20(-0.28--0.12) |
| Montenegro | Cirrhosis due to alcohol use | 3.22 | 5.30 | 64.60 | 1.68(1.46-1.90) |
| Montenegro | Cirrhosis due to NASH | 50.19 | 70.56 | 40.59 | 1.32(1.27-1.38) |
| Montenegro | Cirrhosis due to other causes | 1.74 | 1.80 | 3.45 | -0.03(-0.18-0.12) |
| Morocco | Cirrhosis | 4107.02 | 8284.77 | 101.72 | 1.32(1.21-1.42) |
| Morocco | Cirrhosis due to hepatitis B | 1432.93 | 1550.12 | 8.18 | -1.17(-1.31--1.02) |
| Morocco | Cirrhosis due to hepatitis C | 201.69 | 297.59 | 47.55 | 0.08(-0.09-0.24) |
| Morocco | Cirrhosis due to alcohol use | 10.90 | 26.00 | 138.53 | 1.96(1.73-2.18) |
| Morocco | Cirrhosis due to NASH | 2390.22 | 6312.32 | 164.09 | 2.40(2.35-2.45) |
| Morocco | Cirrhosis due to other causes | 71.28 | 98.74 | 38.52 | -0.11(-0.19--0.03) |
| Mozambique | Cirrhosis | 2349.49 | 3638.47 | 54.86 | -1.36(-1.51--1.21) |
| Mozambique | Cirrhosis due to hepatitis B | 1492.66 | 1921.11 | 28.70 | -2.13(-2.36--1.89) |
| Mozambique | Cirrhosis due to hepatitis C | 287.52 | 423.04 | 47.13 | -1.39(-1.46--1.31) |
| Mozambique | Cirrhosis due to alcohol use | 22.73 | 56.32 | 147.78 | 0.42(0.27-0.56) |
| Mozambique | Cirrhosis due to NASH | 489.86 | 1104.79 | 125.53 | 0.22(0.16-0.28) |
| Mozambique | Cirrhosis due to other causes | 56.71 | 133.21 | 134.90 | 0.32(0.19-0.45) |
| Myanmar | Cirrhosis | 7730.16 | 12331.56 | 59.53 | 0.71(0.64-0.78) |
| Myanmar | Cirrhosis due to hepatitis B | 3081.14 | 3686.33 | 19.64 | -0.64(-0.82--0.45) |
| Myanmar | Cirrhosis due to hepatitis C | 1184.83 | 1224.06 | 3.31 | -0.91(-0.98--0.83) |
| Myanmar | Cirrhosis due to alcohol use | 21.73 | 51.02 | 134.79 | 2.25(2.23-2.26) |
| Myanmar | Cirrhosis due to NASH | 3420.33 | 7336.43 | 114.49 | 2.01(1.93-2.08) |
| Myanmar | Cirrhosis due to other causes | 22.14 | 33.73 | 52.35 | 0.58(0.57-0.59) |
| Namibia | Cirrhosis | 183.11 | 307.67 | 68.02 | -0.08(-0.17-0.02) |
| Namibia | Cirrhosis due to hepatitis B | 96.38 | 114.86 | 19.17 | -1.51(-1.78--1.24) |
| Namibia | Cirrhosis due to hepatitis C | 20.57 | 29.68 | 44.29 | -0.47(-0.58--0.36) |
| Namibia | Cirrhosis due to alcohol use | 1.38 | 3.89 | 181.88 | 2.08(2.04-2.12) |
| Namibia | Cirrhosis due to NASH | 61.11 | 152.24 | 149.12 | 1.58(1.50-1.65) |
| Namibia | Cirrhosis due to other causes | 3.68 | 7.01 | 90.49 | 0.44(0.40-0.47) |
| Nepal | Cirrhosis | 1560.33 | 3406.37 | 118.31 | 1.19(1.01-1.36) |
| Nepal | Cirrhosis due to hepatitis B | 567.84 | 688.67 | 21.28 | -1.08(-1.20--0.97) |
| Nepal | Cirrhosis due to hepatitis C | 99.35 | 123.62 | 24.43 | -0.80(-0.84--0.76) |
| Nepal | Cirrhosis due to alcohol use | 35.06 | 68.87 | 96.43 | 0.86(0.78-0.94) |
| Nepal | Cirrhosis due to NASH | 833.63 | 2488.86 | 198.56 | 2.46(2.24-2.68) |
| Nepal | Cirrhosis due to other causes | 24.44 | 36.35 | 48.73 | -0.29(-0.36--0.22) |
| Netherlands | Cirrhosis | 1473.16 | 2343.39 | 59.07 | 1.27(1.21-1.32) |
| Netherlands | Cirrhosis due to hepatitis B | 124.31 | 112.96 | -9.13 | -0.52(-0.69--0.34) |
| Netherlands | Cirrhosis due to hepatitis C | 53.96 | 67.50 | 25.09 | 0.34(0.26-0.42) |
| Netherlands | Cirrhosis due to alcohol use | 81.51 | 125.03 | 53.39 | 1.02(0.88-1.16) |
| Netherlands | Cirrhosis due to NASH | 1179.64 | 1988.21 | 68.54 | 1.48(1.43-1.53) |
| Netherlands | Cirrhosis due to other causes | 33.74 | 49.69 | 47.27 | 0.88(0.83-0.93) |
| New Zealand | Cirrhosis | 358.50 | 640.76 | 78.73 | 1.21(1.15-1.27) |
| New Zealand | Cirrhosis due to hepatitis B | 103.29 | 149.30 | 44.54 | 0.30(0.24-0.35) |
| New Zealand | Cirrhosis due to hepatitis C | 13.71 | 17.75 | 29.47 | 0.14(0.08-0.19) |
| New Zealand | Cirrhosis due to alcohol use | 5.28 | 8.41 | 59.28 | 0.78(0.76-0.81) |
| New Zealand | Cirrhosis due to NASH | 232.54 | 460.41 | 97.99 | 1.63(1.52-1.73) |
| New Zealand | Cirrhosis due to other causes | 3.68 | 4.89 | 32.88 | 0.07(0.03-0.10) |
| Nicaragua | Cirrhosis | 439.27 | 1201.33 | 173.48 | 2.08(1.97-2.20) |
| Nicaragua | Cirrhosis due to hepatitis B | 67.65 | 77.24 | 14.18 | -1.56(-1.90--1.23) |
| Nicaragua | Cirrhosis due to hepatitis C | 76.53 | 111.10 | 45.17 | -0.46(-0.47--0.44) |
| Nicaragua | Cirrhosis due to alcohol use | 13.36 | 44.33 | 231.81 | 2.67(2.62-2.72) |
| Nicaragua | Cirrhosis due to NASH | 275.28 | 955.77 | 247.20 | 3.09(2.99-3.19) |
| Nicaragua | Cirrhosis due to other causes | 6.46 | 12.88 | 99.38 | 0.68(0.66-0.71) |
| Niger | Cirrhosis | 2121.61 | 4837.27 | 128.00 | -0.68(-0.83--0.54) |
| Niger | Cirrhosis due to hepatitis B | 1382.71 | 2942.21 | 112.79 | -1.06(-1.28--0.84) |
| Niger | Cirrhosis due to hepatitis C | 371.25 | 803.74 | 116.50 | -0.56(-0.66--0.47) |
| Niger | Cirrhosis due to alcohol use | 11.49 | 32.24 | 180.59 | 0.32(0.24-0.40) |
| Niger | Cirrhosis due to NASH | 315.59 | 943.41 | 198.94 | 0.47(0.43-0.50) |
| Niger | Cirrhosis due to other causes | 40.57 | 115.66 | 185.09 | 0.49(0.32-0.67) |
| Nigeria | Cirrhosis | 17864.73 | 35390.05 | 98.10 | -0.78(-0.88--0.67) |
| Nigeria | Cirrhosis due to hepatitis B | 9656.09 | 18113.07 | 87.58 | -1.03(-1.17--0.90) |
| Nigeria | Cirrhosis due to hepatitis C | 3757.21 | 6414.50 | 70.73 | -1.24(-1.33--1.16) |
| Nigeria | Cirrhosis due to alcohol use | 228.18 | 444.91 | 94.98 | -0.66(-0.78--0.54) |
| Nigeria | Cirrhosis due to NASH | 3845.54 | 9550.96 | 148.36 | 0.09(-0.03-0.20) |
| Nigeria | Cirrhosis due to other causes | 377.71 | 866.62 | 129.44 | -0.01(-0.01-0.00) |
| North Korea | Cirrhosis | 4344.69 | 6360.97 | 46.41 | 0.42(0.34-0.49) |
| North Korea | Cirrhosis due to hepatitis B | 2100.50 | 2422.31 | 15.32 | -0.61(-0.76--0.47) |
| North Korea | Cirrhosis due to hepatitis C | 657.10 | 843.33 | 28.34 | 0.13(0.05-0.22) |
| North Korea | Cirrhosis due to alcohol use | 43.36 | 108.99 | 151.36 | 2.50(2.38-2.63) |
| North Korea | Cirrhosis due to NASH | 1518.87 | 2944.61 | 93.87 | 1.56(1.50-1.63) |
| North Korea | Cirrhosis due to other causes | 24.86 | 41.74 | 67.90 | 1.08(1.05-1.11) |
| Northern Mariana Islands | Cirrhosis | 10.81 | 14.64 | 35.43 | 1.19(1.11-1.27) |
| Northern Mariana Islands | Cirrhosis due to hepatitis B | 4.05 | 4.39 | 8.40 | 0.37(0.31-0.43) |
| Northern Mariana Islands | Cirrhosis due to hepatitis C | 0.45 | 0.47 | 4.44 | 0.18(0.02-0.34) |
| Northern Mariana Islands | Cirrhosis due to alcohol use | 0.05 | 0.08 | 60.00 | 2.04(1.82-2.26) |
| Northern Mariana Islands | Cirrhosis due to NASH | 6.22 | 9.66 | 55.31 | 1.69(1.61-1.77) |
| Northern Mariana Islands | Cirrhosis due to other causes | 0.03 | 0.04 | 33.33 | 0.68(0.52-0.84) |
| Norway | Cirrhosis | 389.30 | 616.70 | 58.41 | 0.97(0.88-1.06) |
| Norway | Cirrhosis due to hepatitis B | 44.75 | 43.25 | -3.35 | -0.51(-0.70--0.32) |
| Norway | Cirrhosis due to hepatitis C | 25.16 | 27.01 | 7.35 | -0.40(-0.45--0.35) |
| Norway | Cirrhosis due to alcohol use | 25.46 | 39.60 | 55.54 | 1.02(0.89-1.16) |
| Norway | Cirrhosis due to NASH | 280.54 | 487.54 | 73.79 | 1.25(1.15-1.35) |
| Norway | Cirrhosis due to other causes | 13.39 | 19.30 | 44.14 | 0.81(0.66-0.96) |
| Oman | Cirrhosis | 279.96 | 1325.10 | 373.32 | 2.66(2.34-2.97) |
| Oman | Cirrhosis due to hepatitis B | 82.93 | 186.37 | 124.73 | -0.16(-0.23--0.09) |
| Oman | Cirrhosis due to hepatitis C | 12.62 | 33.37 | 164.42 | 0.40(0.13-0.66) |
| Oman | Cirrhosis due to alcohol use | 0.82 | 3.32 | 304.88 | 2.02(1.65-2.39) |
| Oman | Cirrhosis due to NASH | 178.05 | 1092.36 | 513.51 | 3.66(3.30-4.02) |
| Oman | Cirrhosis due to other causes | 5.55 | 9.68 | 74.41 | -1.23(-1.48--0.98) |
| Pakistan | Cirrhosis | 16922.97 | 33821.32 | 99.85 | 0.06(-0.03-0.14) |
| Pakistan | Cirrhosis due to hepatitis B | 4727.64 | 7198.53 | 52.26 | -1.19(-1.34--1.04) |
| Pakistan | Cirrhosis due to hepatitis C | 5562.63 | 7979.60 | 43.45 | -1.02(-1.30--0.73) |
| Pakistan | Cirrhosis due to alcohol use | 35.05 | 81.77 | 133.30 | 0.59(0.57-0.62) |
| Pakistan | Cirrhosis due to NASH | 6445.47 | 18265.04 | 183.38 | 1.40(1.31-1.50) |
| Pakistan | Cirrhosis due to other causes | 152.17 | 296.38 | 94.77 | -0.04(-0.13-0.06) |
| Palestine | Cirrhosis | 316.17 | 935.50 | 195.89 | 1.03(0.95-1.12) |
| Palestine | Cirrhosis due to hepatitis B | 108.56 | 192.08 | 76.93 | -0.57(-0.75--0.39) |
| Palestine | Cirrhosis due to hepatitis C | 15.04 | 32.38 | 115.29 | -0.32(-0.41--0.22) |
| Palestine | Cirrhosis due to alcohol use | 0.65 | 2.41 | 270.77 | 1.72(1.48-1.97) |
| Palestine | Cirrhosis due to NASH | 185.71 | 692.93 | 273.12 | 1.76(1.72-1.81) |
| Palestine | Cirrhosis due to other causes | 6.21 | 15.70 | 152.82 | 0.30(0.23-0.37) |
| Panama | Cirrhosis | 286.83 | 676.87 | 135.98 | 1.37(1.33-1.42) |
| Panama | Cirrhosis due to hepatitis B | 55.24 | 61.36 | 11.08 | -1.58(-1.88--1.28) |
| Panama | Cirrhosis due to hepatitis C | 30.09 | 45.92 | 52.61 | -0.17(-0.21--0.13) |
| Panama | Cirrhosis due to alcohol use | 8.93 | 28.64 | 220.72 | 2.31(2.22-2.39) |
| Panama | Cirrhosis due to NASH | 188.88 | 533.25 | 182.32 | 2.06(2.00-2.12) |
| Panama | Cirrhosis due to other causes | 3.70 | 7.70 | 108.11 | 0.75(0.66-0.85) |
| Papua New Guinea | Cirrhosis | 956.11 | 2266.31 | 137.03 | 0.21(0.14-0.28) |
| Papua New Guinea | Cirrhosis due to hepatitis B | 473.94 | 887.70 | 87.30 | -0.68(-0.79--0.57) |
| Papua New Guinea | Cirrhosis due to hepatitis C | 137.98 | 266.38 | 93.06 | -0.52(-0.58--0.45) |
| Papua New Guinea | Cirrhosis due to alcohol use | 2.87 | 9.12 | 217.77 | 1.34(1.23-1.45) |
| Papua New Guinea | Cirrhosis due to NASH | 337.57 | 1092.79 | 223.72 | 1.39(1.35-1.43) |
| Papua New Guinea | Cirrhosis due to other causes | 3.74 | 10.32 | 175.94 | 0.76(0.74-0.77) |
| Paraguay | Cirrhosis | 384.10 | 906.51 | 136.01 | 1.17(1.11-1.23) |
| Paraguay | Cirrhosis due to hepatitis B | 111.51 | 199.89 | 79.26 | -0.03(-0.27-0.22) |
| Paraguay | Cirrhosis due to hepatitis C | 50.58 | 86.83 | 71.67 | -0.02(-0.07-0.03) |
| Paraguay | Cirrhosis due to alcohol use | 5.76 | 15.96 | 177.08 | 1.42(1.27-1.56) |
| Paraguay | Cirrhosis due to NASH | 211.21 | 593.02 | 180.77 | 1.93(1.89-1.98) |
| Paraguay | Cirrhosis due to other causes | 5.03 | 10.81 | 114.91 | 0.75(0.69-0.82) |
| Peru | Cirrhosis | 1945.31 | 4284.56 | 120.25 | 1.45(1.42-1.47) |
| Peru | Cirrhosis due to hepatitis B | 518.82 | 719.58 | 38.70 | -0.47(-0.53--0.41) |
| Peru | Cirrhosis due to hepatitis C | 230.19 | 323.00 | 40.32 | -0.35(-0.37--0.32) |
| Peru | Cirrhosis due to alcohol use | 68.07 | 173.15 | 154.37 | 1.97(1.85-2.09) |
| Peru | Cirrhosis due to NASH | 1073.44 | 2967.77 | 176.47 | 2.39(2.34-2.44) |
| Peru | Cirrhosis due to other causes | 54.79 | 101.05 | 84.43 | 0.64(0.60-0.68) |
| Philippines | Cirrhosis | 10072.82 | 18181.44 | 80.50 | 0.43(0.38-0.49) |
| Philippines | Cirrhosis due to hepatitis B | 5307.61 | 7093.03 | 33.64 | -0.71(-0.82--0.60) |
| Philippines | Cirrhosis due to hepatitis C | 822.43 | 1368.68 | 66.42 | 0.20(0.16-0.25) |
| Philippines | Cirrhosis due to alcohol use | 13.63 | 55.36 | 306.16 | 3.37(3.32-3.42) |
| Philippines | Cirrhosis due to NASH | 3912.77 | 9626.35 | 146.02 | 1.57(1.52-1.62) |
| Philippines | Cirrhosis due to other causes | 16.37 | 38.02 | 132.25 | 1.22(1.16-1.28) |
| Poland | Cirrhosis | 3975.40 | 5577.34 | 40.30 | 1.40(1.36-1.44) |
| Poland | Cirrhosis due to hepatitis B | 628.12 | 565.03 | -10.04 | -0.33(-0.52--0.14) |
| Poland | Cirrhosis due to hepatitis C | 349.71 | 332.43 | -4.94 | 0.06(-0.07-0.19) |
| Poland | Cirrhosis due to alcohol use | 317.18 | 515.79 | 62.62 | 2.15(1.96-2.35) |
| Poland | Cirrhosis due to NASH | 2539.96 | 4032.00 | 58.74 | 1.84(1.82-1.86) |
| Poland | Cirrhosis due to other causes | 140.44 | 132.09 | -5.95 | -0.17(-0.26--0.07) |
| Portugal | Cirrhosis | 1335.90 | 1956.04 | 46.42 | 1.14(1.05-1.24) |
| Portugal | Cirrhosis due to hepatitis B | 142.00 | 131.26 | -7.56 | -0.54(-0.64--0.44) |
| Portugal | Cirrhosis due to hepatitis C | 106.57 | 75.26 | -29.38 | -1.42(-1.48--1.36) |
| Portugal | Cirrhosis due to alcohol use | 103.99 | 76.13 | -26.79 | -1.43(-1.51--1.36) |
| Portugal | Cirrhosis due to NASH | 957.33 | 1648.68 | 72.22 | 1.72(1.56-1.88) |
| Portugal | Cirrhosis due to other causes | 26.00 | 24.71 | -4.96 | -0.57(-0.65--0.50) |
| Puerto Rico | Cirrhosis | 500.99 | 810.07 | 61.69 | 1.82(1.76-1.89) |
| Puerto Rico | Cirrhosis due to hepatitis B | 41.65 | 43.74 | 5.02 | 0.10(0.06-0.15) |
| Puerto Rico | Cirrhosis due to hepatitis C | 33.07 | 27.41 | -17.12 | -0.94(-1.02--0.86) |
| Puerto Rico | Cirrhosis due to alcohol use | 18.02 | 21.54 | 19.53 | 0.39(0.30-0.49) |
| Puerto Rico | Cirrhosis due to NASH | 400.73 | 707.77 | 76.62 | 2.19(2.09-2.29) |
| Puerto Rico | Cirrhosis due to other causes | 7.51 | 9.61 | 27.96 | 0.69(0.62-0.77) |
| Qatar | Cirrhosis | 103.46 | 964.13 | 831.89 | 1.72(1.63-1.80) |
| Qatar | Cirrhosis due to hepatitis B | 20.06 | 134.06 | 568.30 | 0.39(0.31-0.47) |
| Qatar | Cirrhosis due to hepatitis C | 3.09 | 22.37 | 623.95 | 0.65(0.62-0.68) |
| Qatar | Cirrhosis due to alcohol use | 0.28 | 2.46 | 778.57 | 1.34(1.27-1.41) |
| Qatar | Cirrhosis due to NASH | 79.09 | 800.50 | 912.14 | 2.05(1.96-2.14) |
| Qatar | Cirrhosis due to other causes | 0.93 | 4.74 | 409.68 | -1.11(-1.33--0.88) |
| Romania | Cirrhosis | 3426.60 | 3654.73 | 6.66 | 1.02(0.98-1.05) |
| Romania | Cirrhosis due to hepatitis B | 888.66 | 683.18 | -23.12 | -0.03(-0.18-0.12) |
| Romania | Cirrhosis due to hepatitis C | 448.79 | 360.00 | -19.78 | -0.22(-0.34--0.11) |
| Romania | Cirrhosis due to alcohol use | 300.09 | 309.45 | 3.12 | 0.98(0.83-1.12) |
| Romania | Cirrhosis due to NASH | 1691.48 | 2233.33 | 32.03 | 1.76(1.71-1.81) |
| Romania | Cirrhosis due to other causes | 97.58 | 68.76 | -29.53 | -0.62(-0.67--0.58) |
| Russian Federation | Cirrhosis | 22959.94 | 27732.50 | 20.79 | 0.87(0.83-0.91) |
| Russian Federation | Cirrhosis due to hepatitis B | 6685.11 | 5772.03 | -13.66 | -0.55(-0.67--0.44) |
| Russian Federation | Cirrhosis due to hepatitis C | 2683.75 | 3285.56 | 22.42 | 1.16(0.91-1.41) |
| Russian Federation | Cirrhosis due to alcohol use | 755.81 | 1357.55 | 79.62 | 2.69(2.52-2.86) |
| Russian Federation | Cirrhosis due to NASH | 12381.59 | 16777.81 | 35.51 | 1.32(1.28-1.36) |
| Russian Federation | Cirrhosis due to other causes | 453.68 | 539.56 | 18.93 | 0.91(0.77-1.05) |
| Rwanda | Cirrhosis | 965.78 | 1561.66 | 61.70 | -0.43(-0.53--0.33) |
| Rwanda | Cirrhosis due to hepatitis B | 564.75 | 808.72 | 43.20 | -0.96(-1.11--0.80) |
| Rwanda | Cirrhosis due to hepatitis C | 137.40 | 178.11 | 29.63 | -1.33(-1.43--1.23) |
| Rwanda | Cirrhosis due to alcohol use | 20.19 | 32.97 | 63.30 | -0.20(-0.32--0.09) |
| Rwanda | Cirrhosis due to NASH | 209.02 | 486.70 | 132.85 | 1.20(0.97-1.43) |
| Rwanda | Cirrhosis due to other causes | 34.42 | 55.17 | 60.28 | -0.33(-0.38--0.28) |
| Saint Lucia | Cirrhosis | 14.15 | 32.20 | 127.56 | 2.16(2.10-2.21) |
| Saint Lucia | Cirrhosis due to hepatitis B | 1.54 | 1.84 | 19.48 | -0.54(-0.71--0.37) |
| Saint Lucia | Cirrhosis due to hepatitis C | 1.24 | 1.32 | 6.45 | -0.76(-0.88--0.64) |
| Saint Lucia | Cirrhosis due to alcohol use | 0.36 | 0.82 | 127.78 | 1.91(1.80-2.02) |
| Saint Lucia | Cirrhosis due to NASH | 10.78 | 27.84 | 158.26 | 2.66(2.56-2.76) |
| Saint Lucia | Cirrhosis due to other causes | 0.23 | 0.38 | 65.22 | 0.71(0.64-0.78) |
| Saint Vincent and the Grenadines | Cirrhosis | 11.96 | 22.15 | 85.20 | 2.34(2.27-2.40) |
| Saint Vincent and the Grenadines | Cirrhosis due to hepatitis B | 1.22 | 1.07 | -12.30 | -0.89(-1.05--0.73) |
| Saint Vincent and the Grenadines | Cirrhosis due to hepatitis C | 0.96 | 0.86 | -10.42 | -0.61(-0.66--0.55) |
| Saint Vincent and the Grenadines | Cirrhosis due to alcohol use | 0.21 | 0.46 | 119.05 | 2.56(2.49-2.63) |
| Saint Vincent and the Grenadines | Cirrhosis due to NASH | 9.40 | 19.54 | 107.87 | 2.82(2.72-2.93) |
| Saint Vincent and the Grenadines | Cirrhosis due to other causes | 0.16 | 0.22 | 37.50 | 0.83(0.76-0.91) |
| Samoa | Cirrhosis | 37.11 | 54.75 | 47.53 | 0.88(0.82-0.95) |
| Samoa | Cirrhosis due to hepatitis B | 14.03 | 15.08 | 7.48 | -0.06(-0.22-0.11) |
| Samoa | Cirrhosis due to hepatitis C | 1.92 | 2.13 | 10.94 | -0.36(-0.36--0.35) |
| Samoa | Cirrhosis due to alcohol use | 0.11 | 0.20 | 81.82 | 1.80(1.73-1.88) |
| Samoa | Cirrhosis due to NASH | 20.88 | 37.12 | 77.78 | 1.45(1.38-1.52) |
| Samoa | Cirrhosis due to other causes | 0.17 | 0.23 | 35.29 | 0.51(0.42-0.61) |
| Sao Tome and Principe | Cirrhosis | 33.25 | 50.63 | 52.27 | -0.57(-0.70--0.44) |
| Sao Tome and Principe | Cirrhosis due to hepatitis B | 21.44 | 27.74 | 29.38 | -1.34(-1.54--1.13) |
| Sao Tome and Principe | Cirrhosis due to hepatitis C | 3.93 | 5.13 | 30.53 | -1.02(-1.12--0.93) |
| Sao Tome and Principe | Cirrhosis due to alcohol use | 0.26 | 0.58 | 123.08 | 1.07(0.94-1.20) |
| Sao Tome and Principe | Cirrhosis due to NASH | 6.85 | 16.05 | 134.31 | 1.41(1.35-1.47) |
| Sao Tome and Principe | Cirrhosis due to other causes | 0.77 | 1.12 | 45.45 | -0.55(-0.72--0.39) |
| Saudi Arabia | Cirrhosis | 3087.34 | 10587.19 | 242.92 | 1.85(1.78-1.92) |
| Saudi Arabia | Cirrhosis due to hepatitis B | 933.76 | 1658.71 | 77.64 | -0.48(-0.64--0.33) |
| Saudi Arabia | Cirrhosis due to hepatitis C | 252.46 | 425.72 | 68.63 | -0.78(-0.95--0.62) |
| Saudi Arabia | Cirrhosis due to alcohol use | 6.76 | 24.71 | 265.53 | 2.08(1.95-2.21) |
| Saudi Arabia | Cirrhosis due to NASH | 1850.49 | 8403.19 | 354.11 | 2.84(2.78-2.90) |
| Saudi Arabia | Cirrhosis due to other causes | 43.87 | 74.86 | 70.64 | -0.76(-0.84--0.69) |
| Senegal | Cirrhosis | 1697.27 | 3108.19 | 83.13 | -0.44(-0.56--0.33) |
| Senegal | Cirrhosis due to hepatitis B | 1012.37 | 1559.73 | 54.07 | -1.21(-1.39--1.03) |
| Senegal | Cirrhosis due to hepatitis C | 237.69 | 393.03 | 65.35 | -0.49(-0.52--0.46) |
| Senegal | Cirrhosis due to alcohol use | 8.48 | 22.53 | 165.68 | 1.07(0.87-1.27) |
| Senegal | Cirrhosis due to NASH | 406.61 | 1065.21 | 161.97 | 1.00(0.89-1.11) |
| Senegal | Cirrhosis due to other causes | 32.12 | 67.69 | 110.74 | 0.27(0.13-0.42) |
| Serbia | Cirrhosis | 1273.72 | 1492.14 | 17.15 | 0.89(0.85-0.92) |
| Serbia | Cirrhosis due to hepatitis B | 233.57 | 191.11 | -18.18 | -0.75(-0.87--0.63) |
| Serbia | Cirrhosis due to hepatitis C | 108.48 | 89.97 | -17.06 | -0.40(-0.51--0.29) |
| Serbia | Cirrhosis due to alcohol use | 49.24 | 61.24 | 24.37 | 1.17(1.10-1.24) |
| Serbia | Cirrhosis due to NASH | 864.49 | 1132.15 | 30.96 | 1.37(1.32-1.42) |
| Serbia | Cirrhosis due to other causes | 17.94 | 17.67 | -1.51 | 0.14(0.08-0.19) |
| Seychelles | Cirrhosis | 13.67 | 27.93 | 104.32 | 1.63(1.57-1.69) |
| Seychelles | Cirrhosis due to hepatitis B | 3.72 | 5.00 | 34.41 | 0.23(0.07-0.39) |
| Seychelles | Cirrhosis due to hepatitis C | 1.18 | 1.62 | 37.29 | 0.00(-0.06-0.06) |
| Seychelles | Cirrhosis due to alcohol use | 0.03 | 0.12 | 300.00 | 3.36(3.23-3.49) |
| Seychelles | Cirrhosis due to NASH | 8.70 | 21.14 | 142.99 | 2.22(2.17-2.28) |
| Seychelles | Cirrhosis due to other causes | 0.03 | 0.05 | 66.67 | 0.96(0.89-1.02) |
| Sierra Leone | Cirrhosis | 1098.59 | 1868.23 | 70.06 | -0.86(-1.00--0.72) |
| Sierra Leone | Cirrhosis due to hepatitis B | 709.20 | 1106.87 | 56.07 | -1.23(-1.46--0.99) |
| Sierra Leone | Cirrhosis due to hepatitis C | 158.96 | 223.73 | 40.75 | -1.27(-1.34--1.20) |
| Sierra Leone | Cirrhosis due to alcohol use | 8.90 | 17.34 | 94.83 | -0.45(-0.53--0.36) |
| Sierra Leone | Cirrhosis due to NASH | 200.05 | 481.19 | 140.53 | 0.40(0.30-0.50) |
| Sierra Leone | Cirrhosis due to other causes | 21.48 | 39.10 | 82.03 | -0.36(-0.41--0.30) |
| Singapore | Cirrhosis | 382.10 | 894.73 | 134.16 | 1.14(1.12-1.16) |
| Singapore | Cirrhosis due to hepatitis B | 108.12 | 184.81 | 70.93 | 0.05(-0.01-0.11) |
| Singapore | Cirrhosis due to hepatitis C | 21.07 | 31.02 | 47.22 | -0.66(-0.80--0.51) |
| Singapore | Cirrhosis due to alcohol use | 8.08 | 19.32 | 139.11 | 1.01(0.89-1.14) |
| Singapore | Cirrhosis due to NASH | 226.91 | 627.87 | 176.70 | 1.73(1.67-1.79) |
| Singapore | Cirrhosis due to other causes | 17.92 | 31.72 | 77.01 | 0.00(-0.09-0.09) |
| Slovakia | Cirrhosis | 700.27 | 867.98 | 23.95 | 0.63(0.55-0.72) |
| Slovakia | Cirrhosis due to hepatitis B | 164.34 | 151.42 | -7.86 | -0.48(-0.70--0.25) |
| Slovakia | Cirrhosis due to hepatitis C | 64.88 | 57.31 | -11.67 | -0.34(-0.41--0.27) |
| Slovakia | Cirrhosis due to alcohol use | 72.26 | 95.29 | 31.87 | 1.04(0.97-1.10) |
| Slovakia | Cirrhosis due to NASH | 371.53 | 540.65 | 45.52 | 1.17(1.10-1.24) |
| Slovakia | Cirrhosis due to other causes | 27.27 | 23.31 | -14.52 | -0.78(-0.82--0.75) |
| Slovenia | Cirrhosis | 292.09 | 393.02 | 34.55 | 1.02(0.98-1.05) |
| Slovenia | Cirrhosis due to hepatitis B | 67.00 | 66.30 | -1.04 | -0.33(-0.52--0.14) |
| Slovenia | Cirrhosis due to hepatitis C | 25.38 | 20.15 | -20.61 | -0.99(-1.05--0.94) |
| Slovenia | Cirrhosis due to alcohol use | 26.71 | 24.61 | -7.86 | -0.16(-0.27--0.05) |
| Slovenia | Cirrhosis due to NASH | 165.79 | 276.22 | 66.61 | 1.86(1.77-1.95) |
| Slovenia | Cirrhosis due to other causes | 7.21 | 5.74 | -20.39 | -0.97(-1.03--0.91) |
| Solomon Islands | Cirrhosis | 80.07 | 167.89 | 109.68 | 0.49(0.45-0.54) |
| Solomon Islands | Cirrhosis due to hepatitis B | 49.39 | 83.01 | 68.07 | -0.33(-0.38--0.28) |
| Solomon Islands | Cirrhosis due to hepatitis C | 2.81 | 4.87 | 73.31 | -0.11(-0.19--0.04) |
| Solomon Islands | Cirrhosis due to alcohol use | 0.20 | 0.59 | 195.00 | 1.85(1.77-1.94) |
| Solomon Islands | Cirrhosis due to NASH | 27.34 | 78.67 | 187.75 | 1.66(1.58-1.74) |
| Solomon Islands | Cirrhosis due to other causes | 0.33 | 0.75 | 127.27 | 0.69(0.63-0.75) |
| Somalia | Cirrhosis | 1196.42 | 2730.23 | 128.20 | -0.09(-0.12--0.06) |
| Somalia | Cirrhosis due to hepatitis B | 743.72 | 1594.05 | 114.33 | -0.29(-0.35--0.23) |
| Somalia | Cirrhosis due to hepatitis C | 101.92 | 236.40 | 131.95 | -0.20(-0.28--0.13) |
| Somalia | Cirrhosis due to alcohol use | 11.98 | 30.27 | 152.67 | 0.15(0.02-0.28) |
| Somalia | Cirrhosis due to NASH | 312.32 | 799.01 | 155.83 | 0.35(0.30-0.40) |
| Somalia | Cirrhosis due to other causes | 26.49 | 70.50 | 166.14 | 0.34(0.30-0.39) |
| South Africa | Cirrhosis | 7822.27 | 11167.70 | 42.77 | 0.00(-0.10-0.10) |
| South Africa | Cirrhosis due to hepatitis B | 4123.97 | 3674.46 | -10.90 | -1.55(-1.72--1.38) |
| South Africa | Cirrhosis due to hepatitis C | 548.64 | 659.80 | 20.26 | -0.87(-1.05--0.69) |
| South Africa | Cirrhosis due to alcohol use | 68.19 | 97.25 | 42.62 | -0.26(-0.32--0.20) |
| South Africa | Cirrhosis due to NASH | 2937.46 | 6571.18 | 123.70 | 1.48(1.41-1.54) |
| South Africa | Cirrhosis due to other causes | 144.01 | 165.01 | 14.58 | -0.78(-0.84--0.72) |
| South Korea | Cirrhosis | 7088.87 | 10614.59 | 49.74 | 0.86(0.84-0.87) |
| South Korea | Cirrhosis due to hepatitis B | 2642.50 | 3281.25 | 24.17 | 0.26(0.22-0.30) |
| South Korea | Cirrhosis due to hepatitis C | 519.54 | 535.38 | 3.05 | -0.75(-0.97--0.54) |
| South Korea | Cirrhosis due to alcohol use | 638.36 | 644.53 | 0.97 | -1.13(-1.32--0.93) |
| South Korea | Cirrhosis due to NASH | 2861.21 | 5854.26 | 104.61 | 2.05(2.00-2.09) |
| South Korea | Cirrhosis due to other causes | 427.25 | 299.16 | -29.98 | -2.01(-2.21--1.80) |
| South Sudan | Cirrhosis | 878.93 | 1407.27 | 60.11 | -0.14(-0.17--0.11) |
| South Sudan | Cirrhosis due to hepatitis B | 468.77 | 689.12 | 47.01 | -0.31(-0.42--0.19) |
| South Sudan | Cirrhosis due to hepatitis C | 80.62 | 129.05 | 60.07 | -0.33(-0.38--0.28) |
| South Sudan | Cirrhosis due to alcohol use | 10.32 | 18.80 | 82.17 | 0.17(0.05-0.29) |
| South Sudan | Cirrhosis due to NASH | 295.67 | 527.55 | 78.43 | 0.12(0.05-0.19) |
| South Sudan | Cirrhosis due to other causes | 23.55 | 42.74 | 81.49 | 0.26(0.20-0.31) |
| Spain | Cirrhosis | 5160.36 | 8302.61 | 60.89 | 1.17(1.05-1.29) |
| Spain | Cirrhosis due to hepatitis B | 396.16 | 434.06 | 9.57 | -0.05(-0.17-0.07) |
| Spain | Cirrhosis due to hepatitis C | 674.12 | 551.66 | -18.17 | -1.15(-1.22--1.09) |
| Spain | Cirrhosis due to alcohol use | 423.46 | 465.93 | 10.03 | -0.26(-0.36--0.15) |
| Spain | Cirrhosis due to NASH | 3603.88 | 6783.50 | 88.23 | 1.69(1.49-1.89) |
| Spain | Cirrhosis due to other causes | 62.73 | 67.46 | 7.54 | -0.45(-0.54--0.36) |
| Sri Lanka | Cirrhosis | 2334.37 | 3930.28 | 68.37 | 1.07(1.04-1.10) |
| Sri Lanka | Cirrhosis due to hepatitis B | 696.01 | 739.37 | 6.23 | -0.93(-1.12--0.75) |
| Sri Lanka | Cirrhosis due to hepatitis C | 82.24 | 79.33 | -3.54 | -1.38(-1.66--1.10) |
| Sri Lanka | Cirrhosis due to alcohol use | 8.83 | 20.93 | 137.03 | 1.73(1.51-1.95) |
| Sri Lanka | Cirrhosis due to NASH | 1539.83 | 3078.64 | 99.93 | 1.83(1.77-1.88) |
| Sri Lanka | Cirrhosis due to other causes | 7.47 | 12.01 | 60.78 | 0.65(0.50-0.81) |
| Sudan | Cirrhosis | 4357.79 | 8488.85 | 94.80 | -0.18(-0.24--0.11) |
| Sudan | Cirrhosis due to hepatitis B | 2344.18 | 3096.67 | 32.10 | -1.68(-1.83--1.54) |
| Sudan | Cirrhosis due to hepatitis C | 212.47 | 310.77 | 46.27 | -1.22(-1.26--1.17) |
| Sudan | Cirrhosis due to alcohol use | 9.79 | 23.61 | 141.16 | 0.62(0.45-0.79) |
| Sudan | Cirrhosis due to NASH | 1724.50 | 4915.50 | 185.04 | 1.34(1.27-1.41) |
| Sudan | Cirrhosis due to other causes | 66.85 | 142.29 | 112.85 | 0.18(0.12-0.23) |
| Suriname | Cirrhosis | 39.65 | 90.28 | 127.69 | 1.64(1.57-1.70) |
| Suriname | Cirrhosis due to hepatitis B | 5.23 | 6.84 | 30.78 | -0.63(-0.74--0.53) |
| Suriname | Cirrhosis due to hepatitis C | 3.67 | 4.76 | 29.70 | -0.60(-0.66--0.55) |
| Suriname | Cirrhosis due to alcohol use | 1.13 | 2.80 | 147.79 | 1.93(1.86-2.01) |
| Suriname | Cirrhosis due to NASH | 28.94 | 74.58 | 157.71 | 2.14(2.08-2.20) |
| Suriname | Cirrhosis due to other causes | 0.69 | 1.30 | 88.41 | 0.82(0.78-0.86) |
| Swaziland | Cirrhosis | 151.02 | 213.76 | 41.54 | -0.12(-0.26-0.03) |
| Swaziland | Cirrhosis due to hepatitis B | 83.06 | 87.20 | 4.98 | -1.35(-1.63--1.08) |
| Swaziland | Cirrhosis due to hepatitis C | 13.25 | 16.47 | 24.30 | -0.39(-0.51--0.28) |
| Swaziland | Cirrhosis due to alcohol use | 1.01 | 1.68 | 66.34 | 0.37(0.25-0.49) |
| Swaziland | Cirrhosis due to NASH | 51.03 | 104.56 | 104.90 | 1.38(1.35-1.42) |
| Swaziland | Cirrhosis due to other causes | 2.67 | 3.85 | 44.19 | 0.05(0.00-0.10) |
| Sweden | Cirrhosis | 848.94 | 1352.98 | 59.37 | 1.16(1.07-1.24) |
| Sweden | Cirrhosis due to hepatitis B | 64.40 | 80.58 | 25.12 | 0.14(0.09-0.19) |
| Sweden | Cirrhosis due to hepatitis C | 58.69 | 58.16 | -0.90 | -0.47(-0.53--0.42) |
| Sweden | Cirrhosis due to alcohol use | 51.53 | 88.27 | 71.30 | 1.35(1.23-1.47) |
| Sweden | Cirrhosis due to NASH | 646.46 | 1078.08 | 66.77 | 1.33(1.23-1.44) |
| Sweden | Cirrhosis due to other causes | 27.87 | 47.89 | 71.83 | 1.29(1.20-1.38) |
| Switzerland | Cirrhosis | 629.09 | 1011.22 | 60.74 | 1.08(1.01-1.16) |
| Switzerland | Cirrhosis due to hepatitis B | 77.63 | 87.10 | 12.20 | -0.11(-0.25-0.02) |
| Switzerland | Cirrhosis due to hepatitis C | 42.75 | 48.72 | 13.96 | -0.17(-0.25--0.08) |
| Switzerland | Cirrhosis due to alcohol use | 41.58 | 59.11 | 42.16 | 0.52(0.43-0.61) |
| Switzerland | Cirrhosis due to NASH | 453.18 | 795.42 | 75.52 | 1.41(1.33-1.48) |
| Switzerland | Cirrhosis due to other causes | 13.95 | 20.87 | 49.61 | 0.71(0.64-0.79) |
| Syria | Cirrhosis | 2011.00 | 4144.13 | 106.07 | 1.37(1.23-1.52) |
| Syria | Cirrhosis due to hepatitis B | 623.88 | 700.72 | 12.32 | -0.75(-0.98--0.52) |
| Syria | Cirrhosis due to hepatitis C | 137.86 | 165.62 | 20.14 | -0.93(-1.10--0.76) |
| Syria | Cirrhosis due to alcohol use | 4.23 | 11.56 | 173.29 | 2.14(1.73-2.55) |
| Syria | Cirrhosis due to NASH | 1205.79 | 3209.40 | 166.17 | 2.28(2.19-2.37) |
| Syria | Cirrhosis due to other causes | 39.23 | 56.83 | 44.86 | -0.13(-0.25--0.01) |
| Taiwan | Cirrhosis | 4575.35 | 7508.68 | 64.11 | 1.42(1.38-1.47) |
| Taiwan | Cirrhosis due to hepatitis B | 2002.04 | 2633.61 | 31.55 | 0.61(0.57-0.66) |
| Taiwan | Cirrhosis due to hepatitis C | 652.26 | 708.87 | 8.68 | -0.36(-0.64--0.08) |
| Taiwan | Cirrhosis due to alcohol use | 72.06 | 150.52 | 108.88 | 2.54(2.31-2.77) |
| Taiwan | Cirrhosis due to NASH | 1822.88 | 3972.31 | 117.91 | 2.56(2.48-2.63) |
| Taiwan | Cirrhosis due to other causes | 26.11 | 43.37 | 66.10 | 1.58(1.44-1.73) |
| Tajikistan | Cirrhosis | 980.37 | 1654.72 | 68.79 | -0.13(-0.18--0.07) |
| Tajikistan | Cirrhosis due to hepatitis B | 421.86 | 619.89 | 46.94 | -0.85(-0.99--0.71) |
| Tajikistan | Cirrhosis due to hepatitis C | 302.89 | 430.78 | 42.22 | -0.58(-0.62--0.54) |
| Tajikistan | Cirrhosis due to alcohol use | 15.03 | 37.81 | 151.56 | 1.57(1.46-1.68) |
| Tajikistan | Cirrhosis due to NASH | 222.71 | 535.46 | 140.43 | 1.35(1.19-1.52) |
| Tajikistan | Cirrhosis due to other causes | 17.87 | 30.77 | 72.19 | 0.09(0.06-0.12) |
| Tanzania | Cirrhosis | 3144.06 | 6168.72 | 96.20 | -0.34(-0.43--0.25) |
| Tanzania | Cirrhosis due to hepatitis B | 1670.60 | 2558.42 | 53.14 | -1.33(-1.51--1.15) |
| Tanzania | Cirrhosis due to hepatitis C | 275.90 | 475.15 | 72.22 | -0.75(-0.84--0.67) |
| Tanzania | Cirrhosis due to alcohol use | 45.66 | 108.87 | 138.44 | 0.37(0.23-0.51) |
| Tanzania | Cirrhosis due to NASH | 1051.09 | 2803.15 | 166.69 | 0.93(0.89-0.96) |
| Tanzania | Cirrhosis due to other causes | 100.81 | 223.11 | 121.32 | 0.11(0.04-0.18) |
| Thailand | Cirrhosis | 11092.69 | 21000.75 | 89.32 | 1.68(1.62-1.74) |
| Thailand | Cirrhosis due to hepatitis B | 4037.53 | 4605.77 | 14.07 | 0.12(-0.05-0.30) |
| Thailand | Cirrhosis due to hepatitis C | 1569.64 | 1711.03 | 9.01 | -0.69(-0.85--0.53) |
| Thailand | Cirrhosis due to alcohol use | 30.70 | 86.65 | 182.25 | 3.15(3.10-3.20) |
| Thailand | Cirrhosis due to NASH | 5427.65 | 14555.97 | 168.18 | 2.86(2.81-2.91) |
| Thailand | Cirrhosis due to other causes | 27.16 | 41.32 | 52.14 | 0.77(0.68-0.87) |
| The Bahamas | Cirrhosis | 27.81 | 57.70 | 107.48 | 1.28(1.25-1.31) |
| The Bahamas | Cirrhosis due to hepatitis B | 2.84 | 3.69 | 29.93 | -0.75(-0.93--0.58) |
| The Bahamas | Cirrhosis due to hepatitis C | 1.96 | 2.56 | 30.61 | -0.56(-0.67--0.44) |
| The Bahamas | Cirrhosis due to alcohol use | 0.83 | 1.76 | 112.05 | 1.25(1.17-1.34) |
| The Bahamas | Cirrhosis due to NASH | 21.75 | 48.88 | 124.74 | 1.62(1.58-1.66) |
| The Bahamas | Cirrhosis due to other causes | 0.43 | 0.81 | 88.37 | 0.89(0.85-0.94) |
| The Gambia | Cirrhosis | 185.57 | 414.38 | 123.30 | -0.05(-0.10-0.01) |
| The Gambia | Cirrhosis due to hepatitis B | 121.90 | 244.42 | 100.51 | -0.51(-0.58--0.43) |
| The Gambia | Cirrhosis due to hepatitis C | 9.06 | 17.94 | 98.01 | -0.43(-0.50--0.37) |
| The Gambia | Cirrhosis due to alcohol use | 1.43 | 3.91 | 173.43 | 0.80(0.71-0.88) |
| The Gambia | Cirrhosis due to NASH | 48.90 | 137.87 | 181.94 | 0.96(0.94-0.99) |
| The Gambia | Cirrhosis due to other causes | 4.30 | 10.24 | 138.14 | 0.19(0.12-0.25) |
| Timor-Leste | Cirrhosis | 114.30 | 196.54 | 71.95 | 0.10(0.03-0.16) |
| Timor-Leste | Cirrhosis due to hepatitis B | 45.69 | 60.34 | 32.06 | -0.92(-1.12--0.71) |
| Timor-Leste | Cirrhosis due to hepatitis C | 17.35 | 22.94 | 32.22 | -0.93(-1.04--0.83) |
| Timor-Leste | Cirrhosis due to alcohol use | 0.27 | 0.78 | 188.89 | 2.10(1.99-2.21) |
| Timor-Leste | Cirrhosis due to NASH | 50.66 | 111.63 | 120.35 | 1.08(1.01-1.14) |
| Timor-Leste | Cirrhosis due to other causes | 0.34 | 0.85 | 150.00 | 1.54(1.47-1.61) |
| Togo | Cirrhosis | 795.67 | 1527.95 | 92.03 | -0.25(-0.36--0.13) |
| Togo | Cirrhosis due to hepatitis B | 516.34 | 835.24 | 61.76 | -0.91(-1.12--0.71) |
| Togo | Cirrhosis due to hepatitis C | 116.07 | 186.83 | 60.96 | -0.83(-0.89--0.78) |
| Togo | Cirrhosis due to alcohol use | 5.24 | 13.42 | 156.11 | 0.86(0.74-0.97) |
| Togo | Cirrhosis due to NASH | 140.68 | 458.81 | 226.14 | 1.82(1.79-1.85) |
| Togo | Cirrhosis due to other causes | 17.34 | 33.65 | 94.06 | -0.21(-0.32--0.10) |
| Tonga | Cirrhosis | 24.40 | 32.43 | 32.91 | 0.87(0.84-0.91) |
| Tonga | Cirrhosis due to hepatitis B | 10.04 | 9.84 | -1.99 | -0.10(-0.19--0.01) |
| Tonga | Cirrhosis due to hepatitis C | 1.32 | 1.38 | 4.55 | -0.08(-0.12--0.04) |
| Tonga | Cirrhosis due to alcohol use | 0.07 | 0.12 | 71.43 | 1.96(1.89-2.03) |
| Tonga | Cirrhosis due to NASH | 12.87 | 20.97 | 62.94 | 1.52(1.49-1.56) |
| Tonga | Cirrhosis due to other causes | 0.11 | 0.12 | 9.09 | 0.40(0.34-0.46) |
| Trinidad and Tobago | Cirrhosis | 138.58 | 266.12 | 92.03 | 2.08(2.01-2.14) |
| Trinidad and Tobago | Cirrhosis due to hepatitis B | 12.83 | 14.03 | 9.35 | -0.41(-0.54--0.28) |
| Trinidad and Tobago | Cirrhosis due to hepatitis C | 9.62 | 9.65 | 0.31 | -0.64(-0.72--0.55) |
| Trinidad and Tobago | Cirrhosis due to alcohol use | 2.92 | 5.80 | 98.63 | 1.99(1.86-2.13) |
| Trinidad and Tobago | Cirrhosis due to NASH | 111.32 | 233.78 | 110.01 | 2.47(2.37-2.57) |
| Trinidad and Tobago | Cirrhosis due to other causes | 1.88 | 2.86 | 52.13 | 0.88(0.80-0.96) |
| Tunisia | Cirrhosis | 1308.13 | 2615.24 | 99.92 | 1.58(1.51-1.65) |
| Tunisia | Cirrhosis due to hepatitis B | 388.40 | 378.88 | -2.45 | -0.86(-1.04--0.67) |
| Tunisia | Cirrhosis due to hepatitis C | 39.62 | 74.99 | 89.27 | 1.32(1.18-1.47) |
| Tunisia | Cirrhosis due to alcohol use | 6.04 | 15.23 | 152.15 | 2.46(2.31-2.62) |
| Tunisia | Cirrhosis due to NASH | 845.07 | 2107.02 | 149.33 | 2.30(2.29-2.32) |
| Tunisia | Cirrhosis due to other causes | 29.01 | 39.12 | 34.85 | -0.09(-0.15--0.03) |
| Turkey | Cirrhosis | 9991.40 | 19738.11 | 97.55 | 1.27(1.22-1.32) |
| Turkey | Cirrhosis due to hepatitis B | 2961.09 | 2825.23 | -4.59 | -1.59(-1.75--1.43) |
| Turkey | Cirrhosis due to hepatitis C | 406.17 | 438.01 | 7.84 | -0.61(-0.79--0.43) |
| Turkey | Cirrhosis due to alcohol use | 19.58 | 46.31 | 136.52 | 1.93(1.70-2.15) |
| Turkey | Cirrhosis due to NASH | 6499.90 | 16291.75 | 150.65 | 2.19(2.11-2.27) |
| Turkey | Cirrhosis due to other causes | 104.66 | 136.81 | 30.72 | -0.35(-0.51--0.19) |
| Turkmenistan | Cirrhosis | 735.87 | 1102.17 | 49.78 | 0.36(0.31-0.40) |
| Turkmenistan | Cirrhosis due to hepatitis B | 333.15 | 390.33 | 17.16 | -0.81(-0.97--0.65) |
| Turkmenistan | Cirrhosis due to hepatitis C | 209.65 | 246.48 | 17.57 | -0.44(-0.52--0.36) |
| Turkmenistan | Cirrhosis due to alcohol use | 13.13 | 33.36 | 154.07 | 2.81(2.59-3.02) |
| Turkmenistan | Cirrhosis due to NASH | 167.46 | 414.42 | 147.47 | 2.50(2.41-2.58) |
| Turkmenistan | Cirrhosis due to other causes | 12.49 | 17.58 | 40.75 | 0.29(0.16-0.41) |
| Uganda | Cirrhosis | 2398.78 | 4406.48 | 83.70 | -0.99(-1.11--0.88) |
| Uganda | Cirrhosis due to hepatitis B | 1541.56 | 2401.74 | 55.80 | -1.69(-1.88--1.50) |
| Uganda | Cirrhosis due to hepatitis C | 239.25 | 417.86 | 74.65 | -1.18(-1.26--1.10) |
| Uganda | Cirrhosis due to alcohol use | 33.56 | 78.67 | 134.42 | 0.03(-0.05-0.11) |
| Uganda | Cirrhosis due to NASH | 516.91 | 1344.05 | 160.02 | 0.57(0.42-0.72) |
| Uganda | Cirrhosis due to other causes | 67.49 | 164.16 | 143.24 | 0.22(0.20-0.25) |
| Ukraine | Cirrhosis | 8357.79 | 9234.89 | 10.49 | 1.05(1.00-1.10) |
| Ukraine | Cirrhosis due to hepatitis B | 2314.57 | 2127.86 | -8.07 | 0.28(0.12-0.44) |
| Ukraine | Cirrhosis due to hepatitis C | 1009.07 | 1088.74 | 7.90 | 0.94(0.68-1.20) |
| Ukraine | Cirrhosis due to alcohol use | 292.19 | 462.10 | 58.15 | 2.76(2.56-2.95) |
| Ukraine | Cirrhosis due to NASH | 4581.20 | 5389.57 | 17.65 | 1.32(1.27-1.37) |
| Ukraine | Cirrhosis due to other causes | 160.75 | 166.63 | 3.66 | 0.86(0.68-1.05) |
| United Arab Emirates | Cirrhosis | 359.09 | 3365.60 | 837.26 | 2.24(2.18-2.30) |
| United Arab Emirates | Cirrhosis due to hepatitis B | 71.72 | 387.30 | 440.02 | 0.13(0.09-0.17) |
| United Arab Emirates | Cirrhosis due to hepatitis C | 11.73 | 95.18 | 711.42 | 1.52(1.20-1.85) |
| United Arab Emirates | Cirrhosis due to alcohol use | 1.22 | 11.76 | 863.93 | 1.99(1.47-2.51) |
| United Arab Emirates | Cirrhosis due to NASH | 270.23 | 2855.52 | 956.70 | 2.71(2.67-2.75) |
| United Arab Emirates | Cirrhosis due to other causes | 4.20 | 15.84 | 277.14 | -1.47(-1.66--1.29) |
| United Kingdom | Cirrhosis | 6008.55 | 9376.19 | 56.05 | 1.12(1.00-1.23) |
| United Kingdom | Cirrhosis due to hepatitis B | 625.87 | 624.80 | -0.17 | -0.30(-0.43--0.17) |
| United Kingdom | Cirrhosis due to hepatitis C | 374.25 | 392.97 | 5.00 | -0.12(-0.22--0.02) |
| United Kingdom | Cirrhosis due to alcohol use | 287.91 | 496.73 | 72.53 | 1.30(1.17-1.44) |
| United Kingdom | Cirrhosis due to NASH | 4496.85 | 7545.04 | 67.79 | 1.37(1.24-1.50) |
| United Kingdom | Cirrhosis due to other causes | 223.67 | 316.65 | 41.57 | 0.45(0.35-0.55) |
| United States | Cirrhosis | 26270.31 | 47919.01 | 82.41 | 1.34(1.28-1.41) |
| United States | Cirrhosis due to hepatitis B | 1734.41 | 2440.51 | 40.71 | 0.14(0.05-0.22) |
| United States | Cirrhosis due to hepatitis C | 2383.26 | 3400.44 | 42.68 | 0.13(0.00-0.26) |
| United States | Cirrhosis due to alcohol use | 351.94 | 581.54 | 65.24 | 0.81(0.67-0.96) |
| United States | Cirrhosis due to NASH | 21466.11 | 40973.83 | 90.88 | 1.56(1.47-1.64) |
| United States | Cirrhosis due to other causes | 334.60 | 522.69 | 56.21 | 0.66(0.47-0.84) |
| Uruguay | Cirrhosis | 345.90 | 543.63 | 57.16 | 1.45(1.39-1.52) |
| Uruguay | Cirrhosis due to hepatitis B | 16.96 | 17.00 | 0.24 | -0.34(-0.48--0.19) |
| Uruguay | Cirrhosis due to hepatitis C | 17.53 | 19.38 | 10.55 | 0.10(0.00-0.20) |
| Uruguay | Cirrhosis due to alcohol use | 10.95 | 17.11 | 56.26 | 1.21(1.15-1.27) |
| Uruguay | Cirrhosis due to NASH | 293.87 | 479.81 | 63.27 | 1.62(1.56-1.68) |
| Uruguay | Cirrhosis due to other causes | 6.59 | 10.33 | 56.75 | 1.29(1.24-1.34) |
| Uzbekistan | Cirrhosis | 4173.29 | 6723.68 | 61.11 | 0.07(0.03-0.12) |
| Uzbekistan | Cirrhosis due to hepatitis B | 1893.67 | 2371.02 | 25.21 | -0.99(-1.07--0.91) |
| Uzbekistan | Cirrhosis due to hepatitis C | 1122.08 | 1370.42 | 22.13 | -1.06(-1.22--0.90) |
| Uzbekistan | Cirrhosis due to alcohol use | 72.38 | 190.58 | 163.30 | 2.48(2.19-2.76) |
| Uzbekistan | Cirrhosis due to NASH | 1013.99 | 2683.27 | 164.62 | 2.20(2.05-2.34) |
| Uzbekistan | Cirrhosis due to other causes | 71.18 | 108.40 | 52.29 | 0.05(-0.29-0.40) |
| Vanuatu | Cirrhosis | 36.13 | 72.92 | 101.83 | 0.47(0.34-0.60) |
| Vanuatu | Cirrhosis due to hepatitis B | 20.24 | 31.55 | 55.88 | -0.33(-0.53--0.13) |
| Vanuatu | Cirrhosis due to hepatitis C | 2.64 | 4.41 | 67.05 | -0.48(-0.50--0.46) |
| Vanuatu | Cirrhosis due to alcohol use | 0.11 | 0.29 | 163.64 | 1.21(1.12-1.30) |
| Vanuatu | Cirrhosis due to NASH | 12.96 | 36.31 | 180.17 | 1.52(1.48-1.55) |
| Vanuatu | Cirrhosis due to other causes | 0.17 | 0.36 | 111.76 | 0.34(0.32-0.36) |
| Venezuela | Cirrhosis | 2567.93 | 6546.46 | 154.93 | 1.71(1.62-1.80) |
| Venezuela | Cirrhosis due to hepatitis B | 466.29 | 548.67 | 17.67 | -1.45(-1.60--1.30) |
| Venezuela | Cirrhosis due to hepatitis C | 251.88 | 377.23 | 49.77 | -0.27(-0.34--0.20) |
| Venezuela | Cirrhosis due to alcohol use | 79.43 | 233.33 | 193.76 | 1.81(1.63-2.00) |
| Venezuela | Cirrhosis due to NASH | 1740.90 | 5328.91 | 206.10 | 2.46(2.39-2.53) |
| Venezuela | Cirrhosis due to other causes | 29.43 | 58.32 | 98.17 | 0.47(0.34-0.59) |
| Vietnam | Cirrhosis | 11843.78 | 20564.88 | 73.63 | 0.81(0.68-0.94) |
| Vietnam | Cirrhosis due to hepatitis B | 5210.96 | 6416.24 | 23.13 | -0.55(-0.74--0.36) |
| Vietnam | Cirrhosis due to hepatitis C | 2302.37 | 2776.03 | 20.57 | -0.68(-0.78--0.59) |
| Vietnam | Cirrhosis due to alcohol use | 37.65 | 181.63 | 382.42 | 4.88(4.63-5.14) |
| Vietnam | Cirrhosis due to NASH | 4240.20 | 11092.69 | 161.61 | 2.49(2.41-2.57) |
| Vietnam | Cirrhosis due to other causes | 52.61 | 98.29 | 86.83 | 0.85(0.71-0.99) |
| Virgin Islands, U.S. | Cirrhosis | 13.07 | 20.46 | 56.54 | 1.80(1.72-1.87) |
| Virgin Islands, U.S. | Cirrhosis due to hepatitis B | 1.09 | 1.10 | 0.92 | 0.11(0.03-0.19) |
| Virgin Islands, U.S. | Cirrhosis due to hepatitis C | 0.82 | 0.79 | -3.66 | -0.08(-0.11--0.06) |
| Virgin Islands, U.S. | Cirrhosis due to alcohol use | 0.40 | 0.65 | 62.50 | 1.88(1.72-2.04) |
| Virgin Islands, U.S. | Cirrhosis due to NASH | 10.58 | 17.66 | 66.92 | 2.04(1.95-2.13) |
| Virgin Islands, U.S. | Cirrhosis due to other causes | 0.18 | 0.26 | 44.44 | 1.44(1.39-1.50) |
| Yemen | Cirrhosis | 2346.14 | 5620.79 | 139.58 | 0.14(0.04-0.25) |
| Yemen | Cirrhosis due to hepatitis B | 1198.55 | 1888.20 | 57.54 | -1.61(-1.78--1.45) |
| Yemen | Cirrhosis due to hepatitis C | 217.91 | 355.50 | 63.14 | -1.34(-1.41--1.27) |
| Yemen | Cirrhosis due to alcohol use | 5.25 | 15.38 | 192.95 | 1.06(0.84-1.28) |
| Yemen | Cirrhosis due to NASH | 879.26 | 3257.68 | 270.50 | 2.01(1.94-2.08) |
| Yemen | Cirrhosis due to other causes | 45.16 | 104.03 | 130.36 | 0.01(-0.08-0.10) |
| Zambia | Cirrhosis | 1071.57 | 2141.04 | 99.80 | -0.58(-0.67--0.49) |
| Zambia | Cirrhosis due to hepatitis B | 596.92 | 975.86 | 63.48 | -1.36(-1.50--1.22) |
| Zambia | Cirrhosis due to hepatitis C | 105.43 | 194.48 | 84.46 | -0.90(-0.98--0.81) |
| Zambia | Cirrhosis due to alcohol use | 14.88 | 32.56 | 118.82 | -0.27(-0.46--0.07) |
| Zambia | Cirrhosis due to NASH | 319.71 | 864.66 | 170.45 | 0.65(0.54-0.75) |
| Zambia | Cirrhosis due to other causes | 34.63 | 73.48 | 112.19 | -0.22(-0.32--0.13) |
| Zimbabwe | Cirrhosis | 2206.75 | 2910.35 | 31.88 | -0.39(-0.49--0.29) |
| Zimbabwe | Cirrhosis due to hepatitis B | 1474.96 | 1578.49 | 7.02 | -1.20(-1.35--1.06) |
| Zimbabwe | Cirrhosis due to hepatitis C | 170.64 | 241.36 | 41.44 | 0.29(0.15-0.44) |
| Zimbabwe | Cirrhosis due to alcohol use | 14.91 | 24.40 | 63.65 | 0.59(0.43-0.76) |
| Zimbabwe | Cirrhosis due to NASH | 504.52 | 1008.01 | 99.80 | 1.12(1.06-1.17) |
| Zimbabwe | Cirrhosis due to other causes | 41.73 | 58.09 | 39.20 | 0.15(0.00-0.29) |
