# Supplementary material for: The burden of liver cirrhosis and underlying etiologies: results from the global burden of disease study 2017
Source: Aging (Albany NY). 2021 Jan 12;13(1):279–300. doi: 10.18632/aging.104127 (PMC7835066; doi:10.18632/aging.104127)
Supplement: Supplementary Table 7 [file aging-13-104127-s008.docx]

**Supplementary Table 7. The prevalence cases, age-standardized prevalence, and temporal trend of liver cirrhosis.**

| Characteristics | 1990 | |  | 2017 | |  | 1990–2017 |
| --- | --- | --- | --- | --- | --- | --- | --- |
|  | Prevalence cases No. ×10^3^ (95% UI) | ASR per 100,000 No. (95% UI) |  | Prevalence cases No. ×10^3^ (95% UI) | ASR per 100,000 No. (95% UI) |  | EAPC No. (95% CI) |
| Overall | 859806.2(824380.7-897826.9) | 15938.0(15281.3-16642.7) |  | 1500585.1(1448741.6-556007.5) | 19640.0(18961.4-20365.4) |  | 0.75(0.73-0.77) |
| Sex |  |  |  |  |  |  |  |
| Male | 497119.5(475967.8-518679.3) | 18293.1(17514.7-19086.4) |  | 869833.4(840589.6-902125.6) | 22684.3(21921.7-23526.5) |  | 0.78(0.76-0.80) |
| Female | 362686.7(347656.9-378963.6) | 13547.3(12985.9-14155.3) |  | 630751.7(608217.5-654309.0) | 16572.8(15980.7-17191.7) |  | 0.71(0.68-0.74) |
| Socio-demographic index |  |  |  |  |  |  |  |
| Low | 94685.8(88465.3-100505.6) | 13575.0(12683.2-14409.4) |  | 184884.8(173903.4-195232.8) | 14333.2(13481.9-15135.4) |  | 0.17(0.15-0.19) |
| Low-middle | 162774.5(155126.4-171196.1) | 15588.4(14856.0-16394.9) |  | 306238.4(293912.5-318516.3) | 17964.2(17241.1-18684.4) |  | 0.47(0.44-0.50) |
| Middle | 274254.4(261754.5-287477.2) | 17681.1(16875.2-18533.5) |  | 465401.9(450109.7-481764.9) | 22265.4(21533.8-23048.2) |  | 0.84(0.80-0.89) |
| Middle-high | 204223.0(196299.4-212386.9) | 18365.4(17652.8-19099.6) |  | 348974.6(338232.4-360842.9) | 25154.0(24379.7-26009.5) |  | 1.13(1.09-1.18) |
| High | 118729.6(115131.7-122453.7) | 12291.3(11918.9-12676.9) |  | 187162.1(181501.7-193274.6) | 16420.4(15923.8-16956.7) |  | 1.10(1.05-1.15) |
| Etiology |  |  |  |  |  |  |  |
| Hepatitis B | 333780.7(302793.8-363301.0) | 6187.2(5612.8-6734.4) |  | 431116.3(395729.0-468718.9) | 5642.5(5179.4-6134.7) |  | -0.39(-0.46--0.34) |
| Hepatitis C | 104466.6(92754.6-118648.8) | 1936.5(1719.4-2199.4) |  | 134493.9(118558.2-153823.8) | 1760.3(1551.7-2013.3) |  | -0.39(-0.42--0.36) |
| Alcohol use | 14608.3(13711.3-15577.7) | 270.8(254.2-288.8) |  | 26041.9(24252.8-28011.2) | 340.8(317.4-366.6) |  | 0.84(0.79-0.89) |
| NASH use | 395517.8(379947.3-411747.6) | 7331.6(7043.0-7632.4) |  | 892322.8(858624.9-927954.4) | 11678.9(11237.9-12145.3) |  | 1.74(1.73-1.75) |
| other causes | 11435.6(10525.0-12332.8) | 212.0(195.1-228.6) |  | 16616.0(15165.6-17954.6) | 217.5(198.5-235.0) |  | 0.04(-0.02-0.10) |
| Region |  |  |  |  |  |  |  |
| Asia Pacific–high income | 24927.2(24150.4-25743.5) | 14362.3(13914.7-14832.6) |  | 32679.1(31683.4-33789.1) | 17472.2(16939.9-18065.7) |  | 0.74(0.72-0.76) |
| Central Asia | 13499.3(12852.9-14239.1) | 19352.0(18425.3-20412.5) |  | 18786.0(18042.2-19689.0) | 20660.9(19842.9-21654.0) |  | 0.19(0.16-0.22) |
| East Asia | 282331.3(267461.8-296431.3) | 22431.3(21249.9-23551.6) |  | 437097.0(421139.9-454180.5) | 29420.0(28346.0-30569.8) |  | 0.95(0.87-1.02) |
| South Asia | 117234.1(111749.9-122586.1) | 10573.3(10078.7-11056.0) |  | 227846.1(219321.8-237112.8) | 12781.1(12302.9-13300.9) |  | 0.73(0.68-0.78) |
| Southeast Asia | 82056.6(78101.9-85947.5) | 17580.1(16732.8-18413.7) |  | 153712.2(148324.5-159319.1) | 23272.7(22456.9-24121.6) |  | 1.10(1.06-1.14) |
| Australasia | 2175.9(2096.6-2260.3) | 10734.0(10342.8-11150.3) |  | 3984.6(3846.4-4125.6) | 14034.5(13547.6-14531.3) |  | 1.04(0.98-1.09) |
| Caribbean | 3740.2(3587.6-3899.9) | 10590.6(10158.5-11042.8) |  | 7162.3(6855.5-7473.1) | 15481.0(14817.9-16152.8) |  | 1.51(1.48-1.54) |
| Central Europe | 16411.8(15915.2-16928.1) | 13221.7(12821.6-3637.7) |  | 19332.0(18735.3-19909.0) | 16839.2(16319.4-17341.8) |  | 0.96(0.93-0.98) |
| Eastern Europe | 34660.8(33341.6-36086.0) | 15273.8(14692.5-15901.8) |  | 40422.1(39099.0-41913.1) | 19230.4(18600.9-19939.7) |  | 0.90(0.87-0.94) |
| Western Europe | 44535.2(43087.6-46012.2) | 11547.2(11171.9-11930.2) |  | 66697.6(64440.6-69061.5) | 15404.7(14883.4-15950.6) |  | 1.09(1.00-1.19) |
| Andean Latin America | 4081.7(3930.9-4247.9) | 10640.5(10247.5-11074.0) |  | 9682.7(9385.1-9984.5) | 15757.3(15273.1-16248.5) |  | 1.48(1.46-1.49) |
| Central Latin America | 22705.7(21807.9-23684.0) | 13832.7(13285.8-14428.7) |  | 50325.7(48670.7-52059.5) | 19697.8(19050.0-20376.4) |  | 1.31(1.24-1.39) |
| Southern Latin America | 4206.2(4071.5-4346.8) | 8488.9(8216.9-8772.5) |  | 7920.9(7673.4-8183.0) | 12072.9(11695.7-12472.4) |  | 1.35(1.34-1.36) |
| Tropical Latin America | 18969.0(18081.5-19864.1) | 12361.5(11783.1-12944.8) |  | 39332.2(37906.3-40869.9) | 17980.9(17329.1-18683.9) |  | 1.38(1.33-1.42) |
| North Africa and Middle East | 65692.8(63184.7-68480.2) | 19270.1(18534.4-20087.8) |  | 154371.3(149104.3-159988.0) | 25720.7(24843.2-26656.6) |  | 1.08(1.03-1.14) |
| North America–high income | 28629.0(27602.5-29744.1) | 10198.5(9832.8-10595.7) |  | 51926.9(50118.1-53900.2) | 14388.8(13887.6-14935.6) |  | 1.31(1.25-1.37) |
| Oceania | 1474.4(1374.6-1569.5) | 22833.1(21288.4-24306.7) |  | 3149.2(2875.1-3345.6) | 24988.0(22813.6-26546.2) |  | 0.40(0.34-0.45) |
| Central Sub-Saharan Africa | 11043.6(10010.8-11891.4) | 20070.5(18193.6-21611.4) |  | 21453.1(18431.5-23405.3) | 17632.2(15148.7-19236.7) |  | -0.57(-0.68--0.46) |
| Eastern Sub-Saharan Africa | 27284.0(25413.7-29317.5) | 14242.8(13266.5-15304.4) |  | 53083.1(49619.7-56551.1) | 13501.0(12620.1-14383.0) |  | -0.29(-0.33--0.24) |
| Southern Sub-Saharan Africa | 10896.7(10117.4-11680.6) | 20763.0(19278.2-22256.6) |  | 15345.9(14605.0-16118.5) | 19833.4(18875.8-20831.9) |  | -0.08(-0.16-0.00) |
| Western Sub-Saharan Africa | 43250.7(38022.3-46327.2) | 22498.8(19779.0-24099.2) |  | 86275.2(76873.3-91565.7) | 19887.5(17720.2-21107.0) |  | -0.63(-0.72--0.55) |
